# Supplementary material for: The mitochondrial long non-coding RNA lncMtloop regulates mitochondrial transcription and suppresses Alzheimer’s disease
Source: EMBO J. 2024 Oct 18;43(23):6001–31. doi: 10.1038/s44318-024-00270-7 (PMC11612450; doi:10.1038/s44318-024-00270-7)
Supplement: Supplementary file 1 — Appendix [file 44318_2024_270_MOESM1_ESM.pdf]

The mitochondrial long non-coding RNA *lncMtloop* regulates mitochondrial transcription and suppresses Alzheimer's disease

Wandi Xiong, Kaiyu Xu, Jacquelyne Ka-Li Sun, Siling Liu, Baizhen Zhao, Jie Shi, Karl Herrup, Hei-Man Chow, Lin Lu, and Jiali Li

Appendix

Appendix Table S1-10 P2-4

Appendix Figure S1-9 P5-14

Appendix Table S1. Demographics and neuropathology summary of brain specimen obtained from the University of Pittsburgh Alzheimer's Disease Research Center (ADRC) brain bank

Subject	Age	Sex	Neuropathology Diagnosis (Braak staging)	Amyloid plaques	Neurofibrillary tangles
NC-1	70	M	Unremarkable	No	No
NC-2	72	F	Unremarkable	No	No
NC-3	68	M	Unremarkable	No	No
NC-4	70	F	Unremarkable	No	No
NC-5	73	F	Unremarkable	No	No
NC-6	75	F	Unremarkable	No	No
NC-7	75	M	Unremarkable	No	No
NC-8	70	F	Unremarkable	No	No
AD-1	69	M	IV	Abundant	Abundant
AD-2	70	F	V	Abundant	Abundant
AD-3	78	F	V	Abundant	Abundant
AD-4	80	F	VI	Abundant	Abundant
AD-5	77	M	V	Abundant	Abundant
AD-6	78	M	VI	Abundant	Abundant
AD-7	73	M	IV	Abundant	Abundant
AD-8	70	F	VI	Abundant	Abundant

Appendix Table S2. siRNAs

Name	Sequence (5'-3')	
	Forward	Reverse
si_p32-1	cccaagaugucuggagauutt	aaucuccagacaucuugggtt
si_p32-2	ugaacggcacggaggcuaatt	uuagccuccgugccguucatt
si_p32-3	gagccagaacugacaucaatt	uugaugucaguucuggcuctt
si_GAPDH	uugaugacaagcuucccauucutt	agaaugggaagcuugucaucaatt
si_TFAM-1	gggaagagcagauggcugatt	ucagccaucugcucuucccttt
si_TFAM-2	acaaagaagcugugagcaatt	uugcucacagcuucuuugutt
si_TFAM-3	gguaaagagaagagaauuatt	uaauucucuucucuuuacctt

Appendix Table S3. Primers used for RT-qPCR

Name	Sequence (5'-3')	
	Forward	Reverse
AC027613.1/homo sapiens *lncMtDloop*	tcacccatcaacaaccgcta	tggggacgagaagggatttg
AC027613.1/mouse *lncMtDloop*-sense	tctcgatggtatcgggtcta	ttaggtgattgggttttgcg
AC027613.1/mouse *lncMtDloop*-antisense	tctcgatggtatcgggtcta	ttaggtgattgggttttgcg
mouse β-actin	ggctgtattcccctccatcg	ccagttggtaacaatgccatgt
Human β-actin	catgtacgttgctatccaggc	ctccttaatgtcacgcacgat
Human COXIV	tgcctgtaaatggctgtcctc	cttcctccctctctactcaccat
mouse p32	cacacggaaggagacaaggc	aatttagcctccgtgccgtt
mouse COXIV	atgctttccccacttacgct	tcattggtgcccctgttcat

mouse mtDNA	cctatcacccttgccatcat	gaggctgttgcttgtgtgac
mouse nDNA	atggaaagcctgccatcatg	tccttgttgttcagcatcac
mouse TFAM	tcgcatcccctcgtctatca	ttctggtagctccctccaca
mouse ATP6	acgcctaatcaacaaccgact	tccgtccttttggtgtgtga
mouse ATP8	ttcccactagcaccttcacca	ttgttggggtaatgaatgaggca
mouse Cytb	acctcctatcagccatccca	agcgaagaatcgggtcaagg
mouse COXI	ctacccacctctagccggaa	tgttatggctgggggtttca
mouse COXII	cctggtgaactacgactgct	atttagtcggcctgggatgg
mouse COXIII	attaacccttggcctgctca	aataggagtgtggtggccttg
mouse ND1	tcctatccacgcttccgcta	tgtatggtggtatccccgct
mouse ND2	cacaatatccagcaccaacct	gctgttgcttgtgtgacgaa
mouse ND3	gcggatttgaccctacaagc	tggtagtggaagtagaagggca
mouse ND4	tgggggaaccaaactgaacg	agcgtctaaggtgtgtgttgt
mouse ND5	ccaccaaccaacattccaatcc	tggatgggaaaaaccccagt
mouse ND6	gttagagggagggattggggt	acaaagatcacccagctacca
mouse lncND5	gtaggatgaagccaatattgtgc	atgaggcataaatgaaatataag
mouse 12S rRNA	gttaatgtagcttaataac	gatttcacgttgtgagtct
mouse 7S RNA	ataatttatttatcccactagc	aagcttagggagagctgggttg

Appendix Table S4. Antibodies used in this study

Antibody	Company	Catalog number	Dilution
Mouse monoclonal anti-6E10	BioLegend	Cat# 803001; RRID: AB_2564653	1:300 IF
Mouse monoclonal anti-GC1q R	Abcam	Cat# ab24733; RRID: AB_448269	1:1000 WB, 1:300 IF
Mouse monoclonal anti-COX IV	Abcam	Cat# ab33985; RRID: AB_879754	1:1000 WB
Mouse monoclonal anti-ATP5a	Abcam	Cat# ab14748; RRID: AB_301447	1:1000 WB, 1:300 IF
Rabbit polyclonal anti-TOMM20	Abcam	Cat# ab186735;	1:300 IF
Rabbit polyclonal anti-GFP	Santa Cruz	Cat# sc-8334; RRID: AB_641123	1:300 IF
Rabbit polyclonal anti-LC3B	Abcam	Cat# ab51520; RRID: AB_881429	1:3000 WB
Rabbit polyclonal anti-ULK1	Sigma-Aldrich	Cat# A7481; RRID: AB_1840703	1:1000 WB
Rabbit polyclonal anti-PINK1	Abcam	Cat# ab23707; RRID: AB_447627	1:1000 WB
Rabbit polyclonal anti-parkin	Cell Signaling Technology	Cat# 2132; RRID: AB_10693040	1:1000 WB
Rabbit polyclonal anti-β-actin	Abcam	Cat# ab8227; RRID: AB_2305186	1:3000 WB
Rabbit monoclonal anti-Tppp	Abcam	Cat# ab92305; RRID: AB_2050408	1:1000 WB
Rabbit monoclonal anti-Aly/Ref	Abcam	Cat# ab202894;	1:1000 WB
Rabbit polyclonal anti-MAP2	Abcam	Cat# ab32454; RRID: AB_776174	1:300 IF
Mouse monoclonal anti-ATP5D	Abcam	Cat# ab174438;	1:1000 WB
Rabbit monoclonal anti-syntaxin	Abcam	Cat# ab188583;	1:1000 WB
Mouse monoclonal anti-GFAP	BioLegend	Cat# 837201; AB_2565371	1:300 IF
Rabbit polyclonal anti-Iba1	Wako	Cat# 019-19741; AB_839504	1:300 IF
Rabbit polyclonal anti-TFAM	Abcam	Cat# ab252432	1:500 WB, 1:200 IF
Mouse monoclonal anti-PNPASE	Santa Cruz	Cat# sc-271479	1:500 WB
Total OXPHOS rodent	Abcam	Cat# ab110413	1:1000 WB
Rabbit polyclonal anti-VDAC1	Abcam	Cat# ab15895	1:1000 WB
Mouse monoclonal anti-Drp1	Abcam	Cat# ab56788	1:1000 WB
Rabbit monoclonal anti-Opa1	Abcam	Cat# ab157457	1:1000 WB

Appendix Table S5. Probes used for RNAscope

Name	Sequence (5'-3')
RNAscope probe for Mmu-Mt-AC027613.1 (mouse)	Target Region: 2 - 678
RNAscope probe for Mm-Mt-AC027613.1 (human)	Target Region: 2 - 937

Appendix Table S6. Primers for T7 templates amplification for northern blot probes recognizing noncoding RNAs

Name	Sequence (5'-3')	
	Forward	Reverse
mouse *lncMtDloop*	tatataccatgaatattatc	ccatcgagatgtcttatttaaggggaacgtatgggcga
human lncMtDloop	ataccaacaaacctacccac	catcgtgatgtcttatttaaggggaacgtgtgggctatt
mouse lncND5	gtaggatgaagccaatattgtgcc	cctttttgaaaaagtcatggaggccatg
mouse 7S RNA	ataatttatttatcccactag	aagcttajgggagagctgggttgtttgggttgtg
mouse 12S rRNA	gttaatgtagcttaataacaaagc	ctttgctttttctactttttagattgttctttc
β-actin	caaggccaaccgcgagaaga	atcttcatgaggtagtcagtcaggtcccggccagccaggtcca

Appendix Table S7. Primers used for RIP

Name	Sequence (5'-3')	
	Forward	Reverse
mouse *lncMtDloop*	tctcgatggtatcgggtcta	cgcaaaacccaatcacctaa
12S rRNA	cggcgtaaaacgtgtcaact	agtaccgccaagtcctttga
7S RNA	agtccggcttacagctaaca	gagggtgaatacgtaggcttga

Appendix Table S8. Primers used for ChIP

Name	Sequence (5'-3')	
	Forward	Reverse
mouse *lncmtdloop*	gcatgaaaggacagcacaca	taggtgattgggttttgcgg

Appendix Table S9. Primers used for EMSA

Name	Sequence (5'-3')	
	Forward	Reverse
mouse *lncMtDloop*	aaactctaatcacactctattac gcaataaatattaacaa	ttgttaatatttattgcgtaataga gtgtgattagagttt
Antisense of lncMtDloop	ttgttaatatttattgcgtaataga gtgtgattagagttt	aaactctaatcacactctattac gcaataaatattaacaa

Appendix Table S10. Soft and algorithms

**Soft and algorithms**	**Websites**
RNAfold WebServer	http://rna.tbi.univie.ac.at/cgi-bin/RNAfold.cgi
Coding Potential Assessment Tool	http://lilab.research.bcm.edu/cpat/index.php
Coding Potential Calculator	http://cpc.cbi.pku.edu.cn/
catRAPID	http://service.tartaglialab.com/page/catrapid.
ImageJ	http://imagej.nih.gov/ij/
Clampex 10.7	N/A
GraphPad Prism	https://www.graphpad.com/
Leica software package	N/A

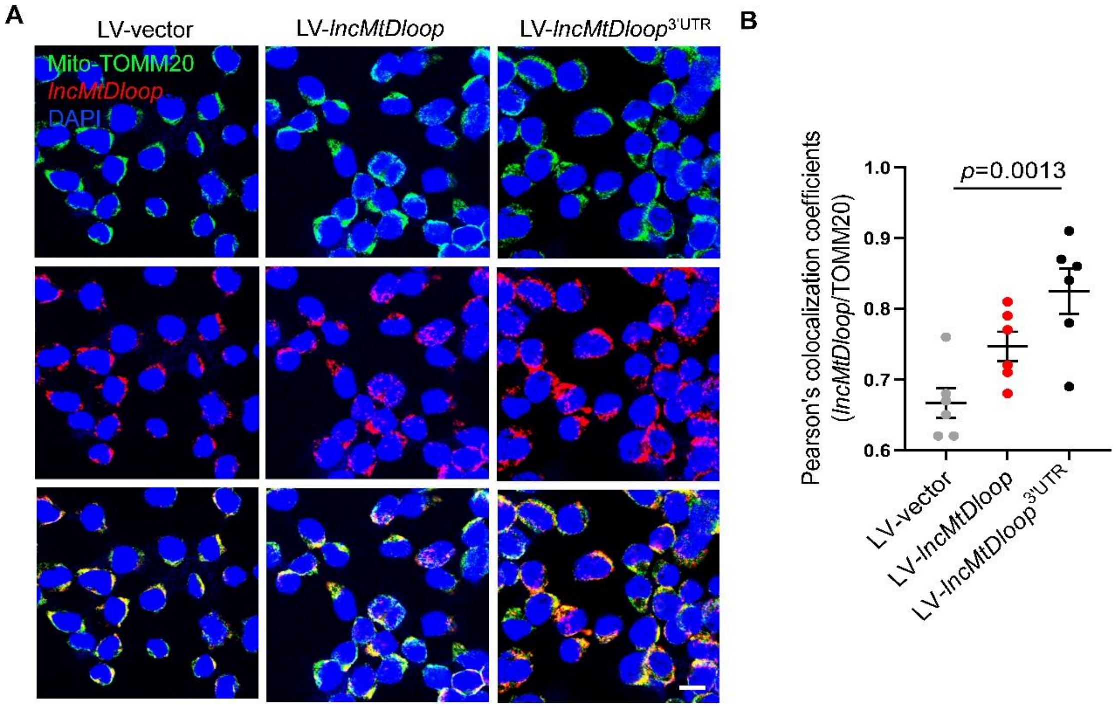

Appendix Fig S1, related to Fig 2. Facilitation of *lncMtDloop* import into mitochondria by *MRPS12* 3'UTR

A. Representative images depicting the co-localization of *lncMtDloop* (red) and TOMM20 (green) visualized using RNAscope ISH and immunostaining in N2a cells. Scale bars, 10 µm.

B. Quantification of colocalization as observed in panel A). Error bars indicate the mean ± SEM, with n = 6 regions of interest (ROI) per group, one-way ANOVA with Dunnett's multiple comparison test.

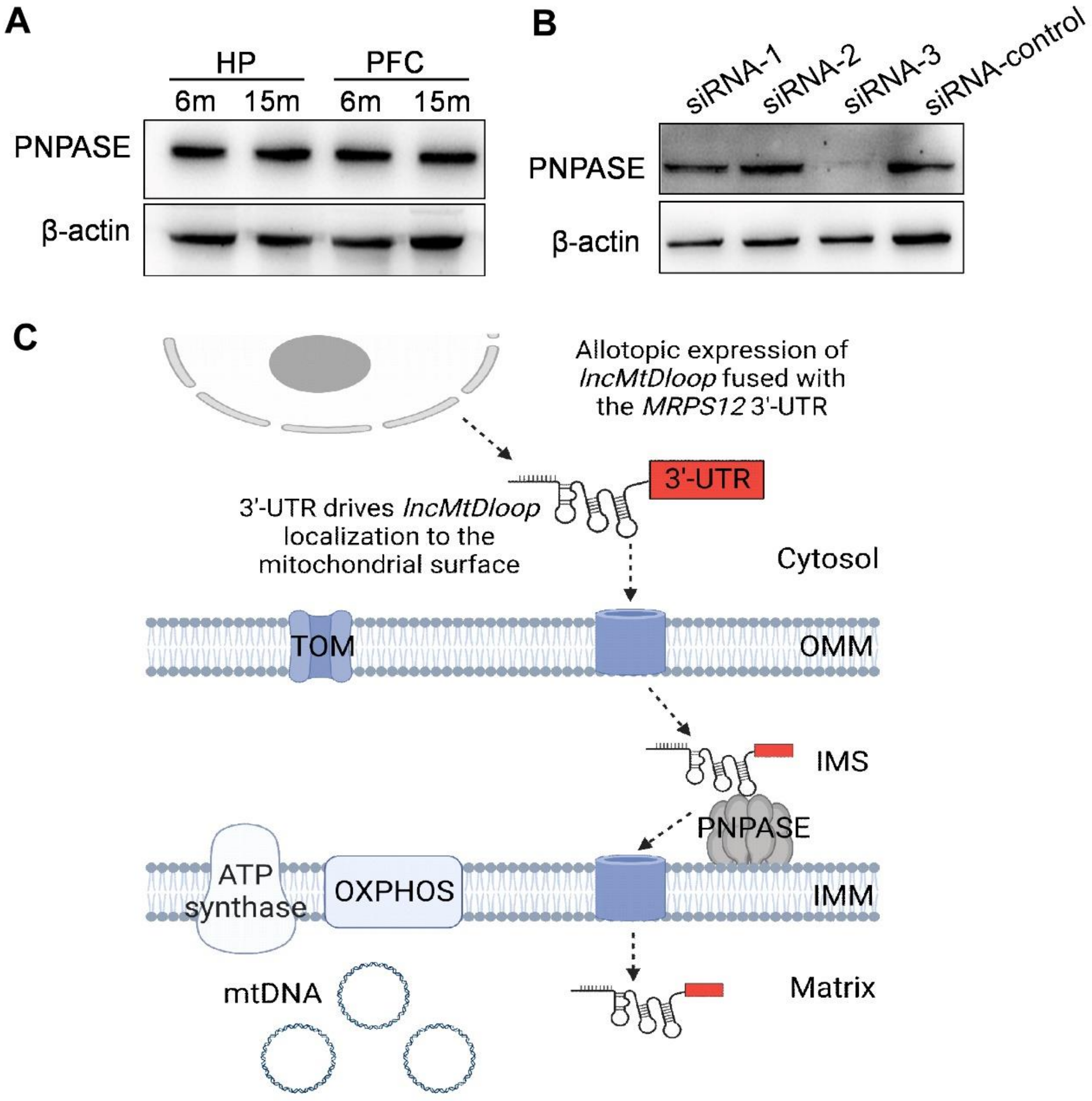

Appendix Fig S2, related to Fig 2. Facilitation of *lncMtDloop* import into mitochondria by PNPASE.

A Western blot analysis showcasing the steady-state expression of PNPASE protein in 6-month (6M) and 15-month (15M) samples.

B Knockdown of PNPASE in N2a cells through the use of three siRNAs.

C PNPASE's role in facilitating the import of alternatively expressed *lncMtDloop* into the mitochondrial matrix by binding to specific stem-loop structures within the RNAs.

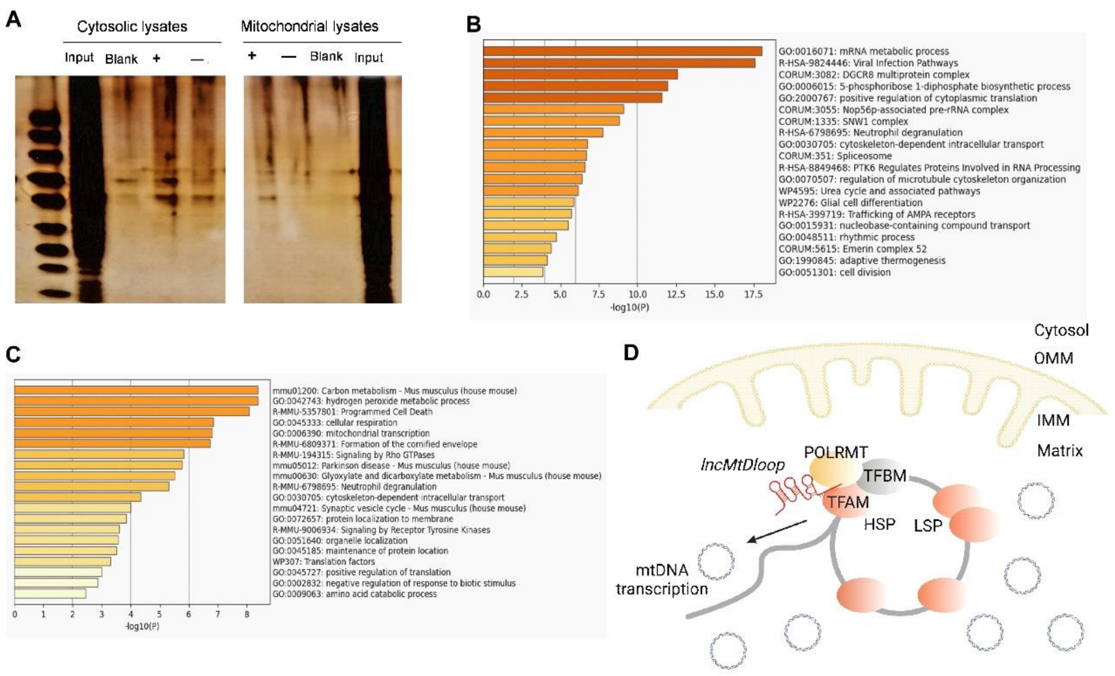

Appendix Fig S3, related to Fig 3. Identification of *lncMtDloop* molecular binding partners.

A Silver-stained gel presenting *lncMtDloop* RNA pull-down assays conducted using mitochondrial and cytosolic lysate preparations. Distinct bands were excised for subsequent mass spectrum analysis. "+" denotes *lncMtDloop* probe; "-" signifies anti-*lncMtDloop* control.

B Enrichment analysis results for Gene Ontology (GO) and Kyoto Encyclopedia of Genes and Genomes (KEGG) pathways in the cytosolic fraction. These proteins were subjected to GO and KEGG pathway enrichment analysis by Metascape.

C Enrichment analysis results for GO and KEGG pathways in the mitochondrial fraction. These proteins were subjected to GO and KEGG pathway enrichment analysis by Metascape.

D Schematic representation depicting the regulatory impact of *lncMtDloop* on TFAM, highlighting its mode of interaction.

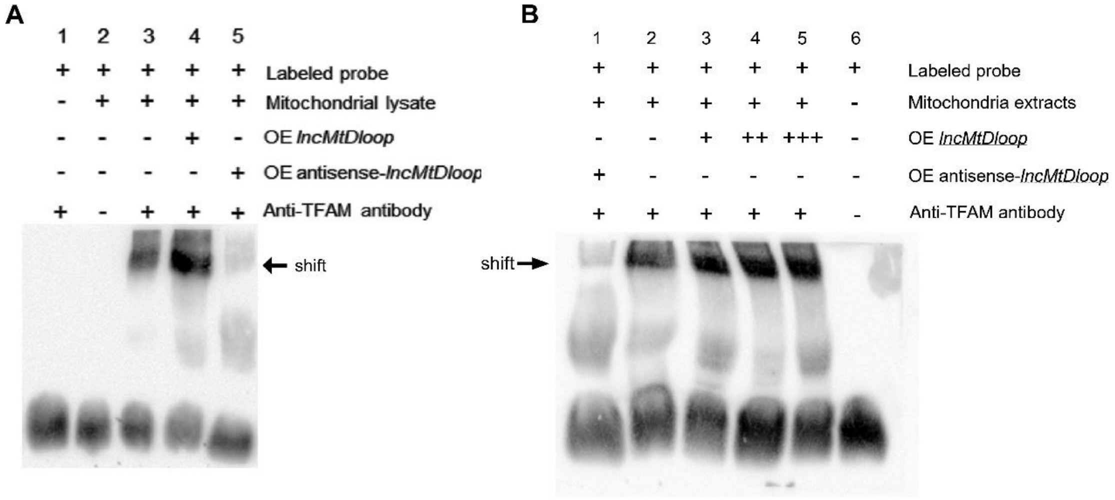

Appendix Fig S4, related to Fig 3. EMSA identifies that TFAM interacts with *lncMtDloop*.

A. Mitochondrial lysates from N2a cells overexpressing (OE) *lncMtDloop* or its antisense were incubated with labeled probes, followed by EMSA assays. The arrow indicates the shifted bands.

B. Mitochondrial lysates from N2a cells with dose-dependent overexpression (OE) of *lncMtDloop* were incubated with labeled probes, followed by EMSA assays. The arrow indicates the shifted bands.

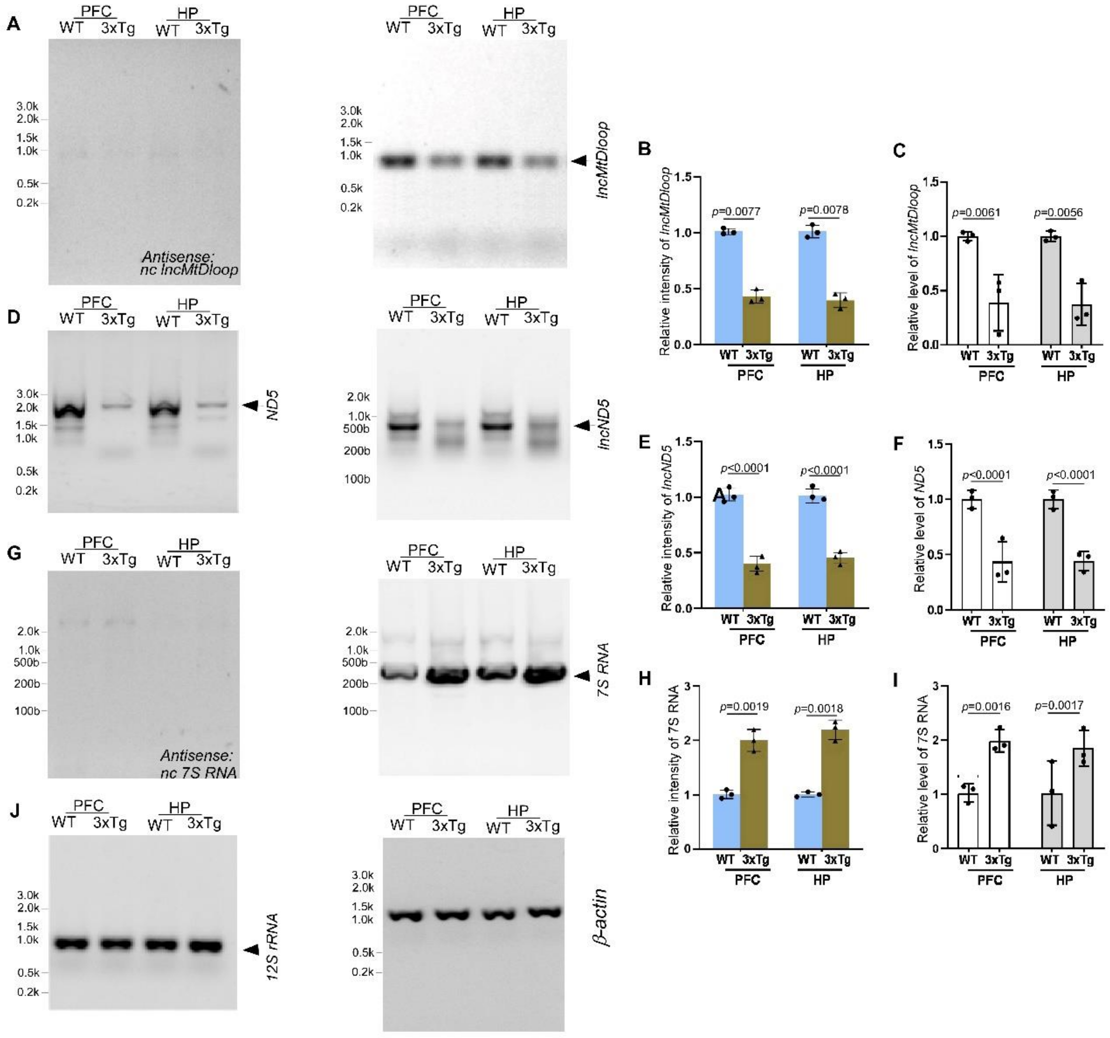

Appendix Fig S5, related to Fig 4. Distinct mitochondrial noncoding RNAs show differential patterns of their expression in the brains of AD mice

A. RNA isolated from PFC and hippocampal tissues of 12-month-old wild-type and 3xTg mouse brains were analyzed by Northern blotting. The analysis focused on *lncMtDloop* and its complementary antisense RNA.

B. Quantification of northern blot signal intensities shown in A), assessed using Image J. Error bars denote mean ± SEM, with n = 3 mice per genotype. n.s., no significance, unpaired *t*-test.

C. RT-qPCR validation of *lncMtDloop* expression. Total RNAs were extracted from fresh PFC and hippocampal tissues of 12-month-old wild-type (WT) and 3xTg mice. Error bars represent mean ± SEM, with n = 3 mice per group. n.s., no significance, unpaired *t*-test.

D. RNA isolated from PFC and hippocampal tissues of 12-month-old wild-type and

3xTg mouse brains were analyzed by Northern blotting. The analysis focused on mitochondrial *lncND5* and its complementary coding RNA.

E. Quantification of northern blot signal intensities shown in D), assessed using Image J. Error bars denote mean ± SEM, with n = 3 mice per genotype. n.s., no significance, unpaired *t*-test.

F. RT-qPCR validation of *lncND5* expression. Total RNAs were extracted from fresh PFC and hippocampal tissues of 12-month-old wild-type (WT) and 3xTg mice. Error bars represent mean ± SEM, with n = 3 mice per group. n.s., no significance, unpaired *t*-test.

G. RNA isolated from PFC and hippocampal tissues of 12-month-old wild-type and 3xTg mouse brains were analyzed by Northern blotting. The analysis focused on 7S RNA and its complementary antisense RNA.

H. Quantification of northern blot signal intensities shown in G), assessed using Image J. Error bars denote mean ± SEM, with n = 3 mice per genotype. n.s., no significance, unpaired *t*-test.

I. RT-qPCR validation of 7S RNA expression. Total RNAs were extracted from fresh PFC and hippocampal tissues of 12-month-old wild-type (WT) and 3xTg mice. Error bars represent mean ± SEM, with n = 3 mice per group. n.s., no significance, unpaired *t*-test.

J. RNA isolated from PFC and hippocampal tissues of 12-month-old wild-type and 3xTg mouse brains were analyzed by Northern blotting. 12S rRNA and β-actin were used as a control.

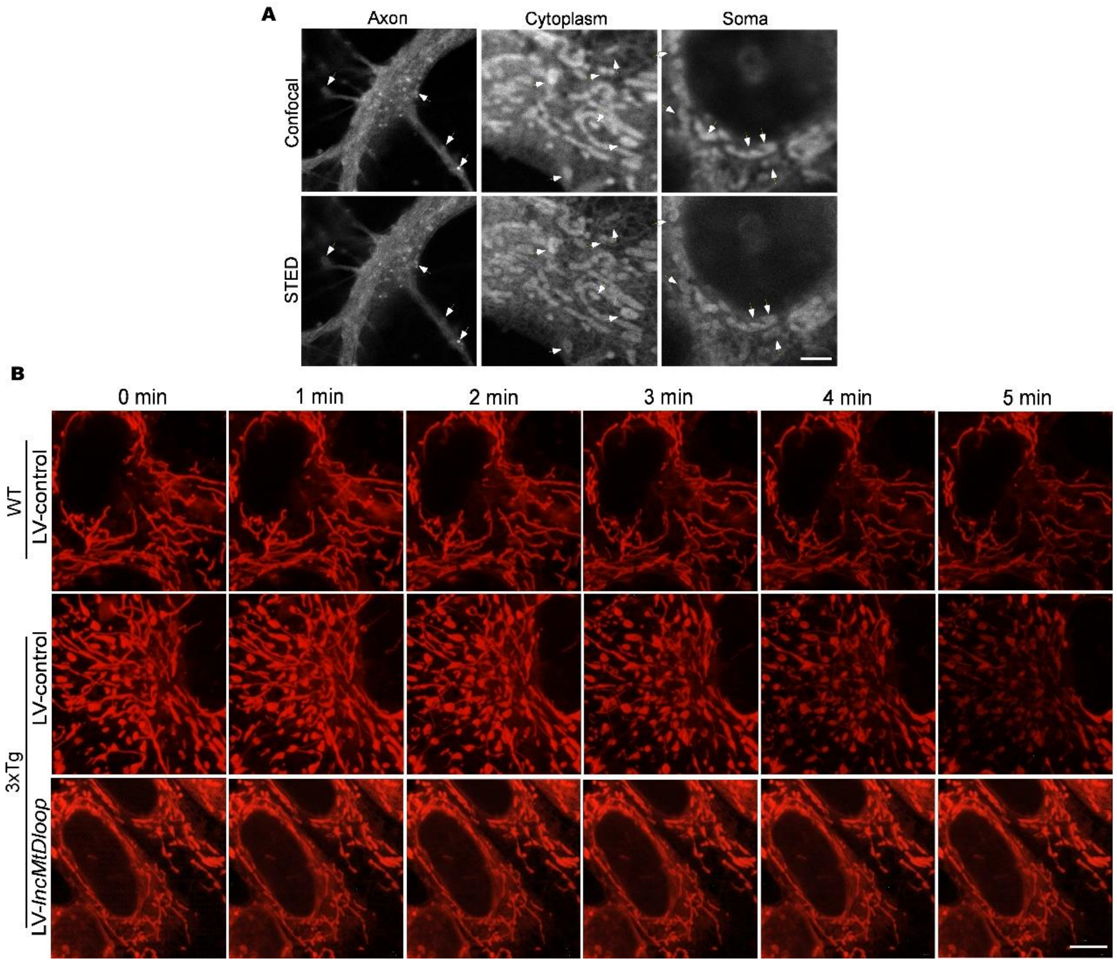

Appendix Fig S6, related to Fig 5. Facilitation of *lncMtDloop* import into mitochondria by *MRPS12* 3'UTR

A. Confocal and STED images were acquired under consistent imaging conditions, providing a clear view of the axon, cytoplasm, and soma within living neurons. Scale bars are set at 5 μm for reference. White arrows highlight key neuronal cell structures for easy identification.

B. Time-lapse STED imaging showcasing mitochondrial dynamics using MitoESq-635 staining. Scale bars, 5 μm.

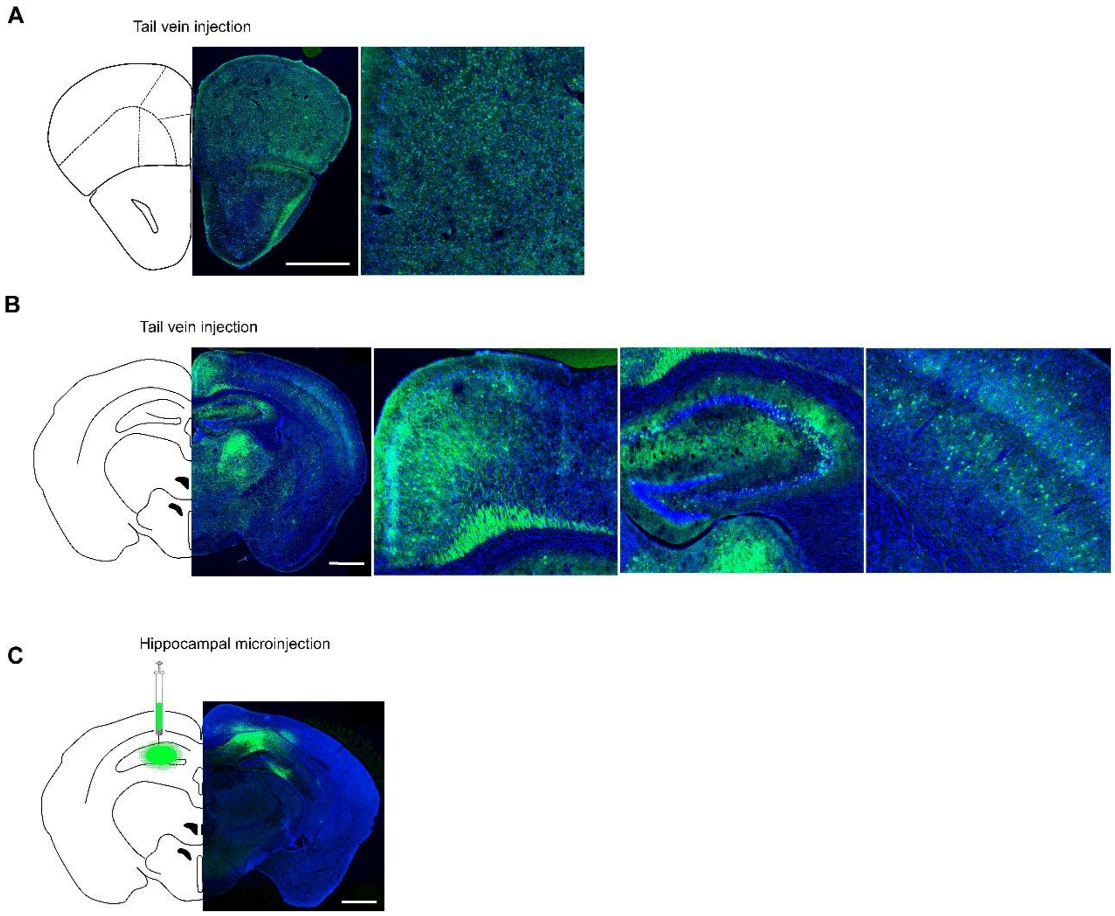

Appendix Fig S7, related to Fig 6 and 7. Whole-brain fluorescence images of AAV9- and AAV-PHP.eB-*lncMtDloop* expression at 60 Days, related to Fig 6 and 7.

A. and B. Intravenous (IV) administration of AAV-PHP.eB-*lncMtDloop* viral particles carrying the eGFP tag via the lateral tail veins of mice. Representative fluorescence images along with DAPI staining in the prefrontal cortex (PFC) A) and hippocampus B) regions. Scale bars indicate 1mm.

C. Injection of AAV9 viral particles carrying the eGFP tag *lncMtDloop* into the hippocampus of mice. Whole-brain fluorescence images depicting the distribution of the injected particles. Scale bars indicate 1mm.

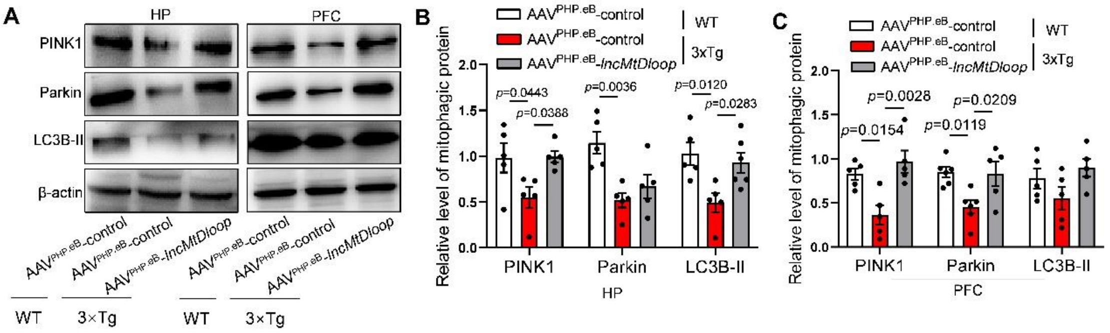

Appendix Fig S8, related to Fig 6. Enhancement of mitophagy activation in the brains of 3xTg mice through reinstated *lncMtDloop* expression

A. Western blot analysis depicting the levels of PINK1, Parkin, and LC3B-II in hippocampal tissues from 12-month-old wild type and 3xTg mice w/o restoration of *lncMtDloop*.

B. and C. The relative intensities of PINK1, Parkin, and LC3B-II signals, as presented in a), are graphically represented. Error bars indicate mean ± SEM, with n = 5-6 animals per group, one-way ANOVA with Dunnett's multiple comparisons test.

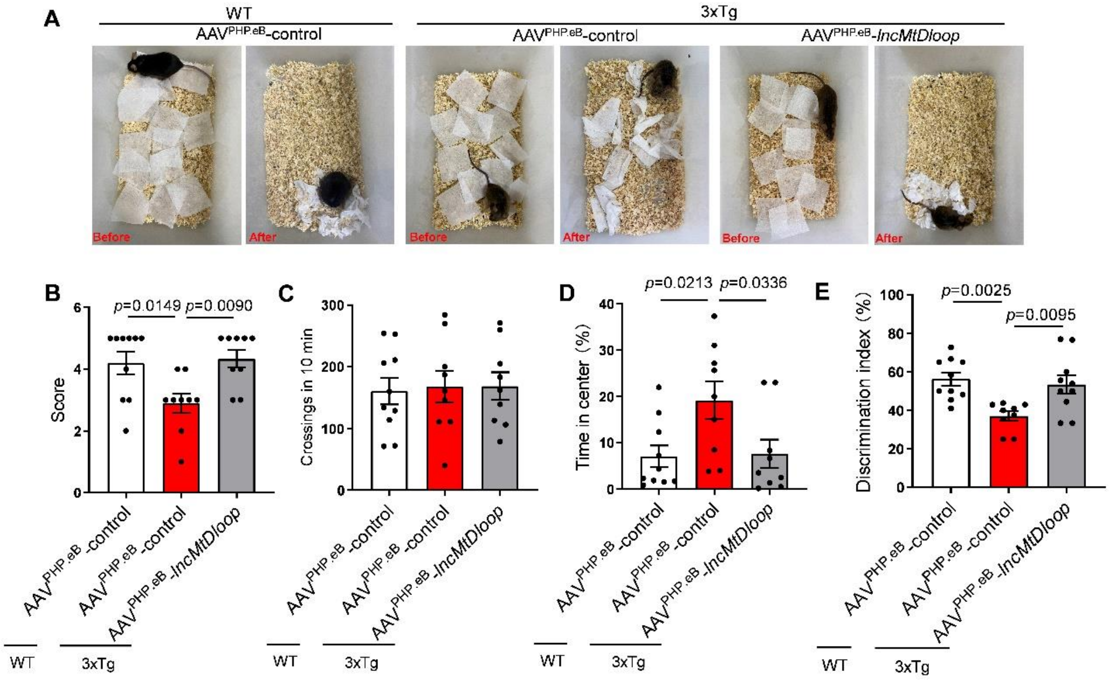

Appendix Fig S9, related to Fig 7. Alleviation of behavioral deficits in 3xTg mice through the global restoration of *lncMtDloop* expression

A. The impact of reinstated *lncMtDloop* expression on 3×Tg mice was assessed through the nest test. Error bars represent mean ± SEM, with n = 9-10 animals per group. **P*<0.05, one-way ANOVA with Dunnett's multiple comparisons test.

B. and C. Performance of wild type and 3xTg mice w/o restoration of *lncMtDloop* showing open field activities including crossings and time in the center in 10 minutes. Bars indicate mean ± SEM, n = 9-10 animals per group, one-way ANOVA with Dunnett's multiple comparisons test.

D. Performance of wild type and 3xTg mice w/o restoration of *lncMtDloop* showing discrimination index of NOR test. Bars indicate mean ± SEM, n = 9-10 animals per group, one-way ANOVA with Dunnett's multiple comparisons test.
