# Supplementary material for: The mitochondrial long non-coding RNA lncMtloop regulates mitochondrial transcription and suppresses Alzheimer’s disease
Source: EMBO J. 2024 Oct 18;43(23):6001–31. doi: 10.1038/s44318-024-00270-7 (PMC11612450; doi:10.1038/s44318-024-00270-7)
Supplement: Supplementary file 3 — Movie EV1 [file 44318_2024_270_MOESM3_ESM.zip › Movie EV1/Movie EV1.docx]

**Movie EV1: Imaging video showcasing mitochondrial dynamics using MitoESq-635 staining in wild-type primary hippocampal neurons**

This video presents the dynamic behavior of mitochondria in wild-type primary hippocampal neurons at 14 days *in vitro* (DIV 14), following the expression of a control AAV vector. The mitochondria were labeled using MitoESq-635, a specific mitochondrial stain. The video captures mitochondrial movement and morphological changes over a 5-minute recording period, providing insights into the mitochondrial dynamics under normal physiological conditions.
