# Supplementary material for: The mitochondrial long non-coding RNA lncMtloop regulates mitochondrial transcription and suppresses Alzheimer’s disease
Source: EMBO J. 2024 Oct 18;43(23):6001–31. doi: 10.1038/s44318-024-00270-7 (PMC11612450; doi:10.1038/s44318-024-00270-7)
Supplement: Supplementary file 4 — Movie EV2 [file 44318_2024_270_MOESM4_ESM.zip › Movie EV2/Movie EV2.docx]

**Movie EV2: Imaging video showcasing mitochondrial dynamics using MitoESq-635 staining in 3xTg primary hippocampal neurons**

This video illustrates mitochondrial dynamics in primary hippocampal neurons derived from 3xTg mice at 14 days in vitro (DIV 14), following the expression of a control AAV vector. The mitochondria were stained with MitoESq-635 to visualize their movement and morphology. The 3xTg mouse model, which carries three mutations linked to Alzheimer's disease, provides insights into the alterations in mitochondrial function under these pathological conditions. The 5-minute recording captures the changes in mitochondrial behavior in these neurons.
