# Supplementary material for: The mitochondrial long non-coding RNA lncMtloop regulates mitochondrial transcription and suppresses Alzheimer’s disease
Source: EMBO J. 2024 Oct 18;43(23):6001–31. doi: 10.1038/s44318-024-00270-7 (PMC11612450; doi:10.1038/s44318-024-00270-7)
Supplement: Supplementary file 5 — Movie EV3 [file 44318_2024_270_MOESM5_ESM.zip › Movie EV3/Movie EV3.docx]

**Movie EV3: Imaging video showcasing mitochondrial dynamics using MitoESq-635 staining in 3xTg primary hippocampal neurons with the restoration of *lncMtDloop***

This video shows mitochondrial dynamics in DIV 14 primary hippocampal neurons from 3xTg mice following the restoration of *lncMtDloop*. The mitochondria, stained with MitoESq-635, exhibit changes in their movement and morphology over a 5-minute recording period, highlighting the impact of *lncMtDloo*p restoration on mitochondrial function in the context of the 3xTg Alzheimer's disease model.
