# Supplementary figures and images for: The mitochondrial long non-coding RNA lncMtloop regulates mitochondrial transcription and suppresses Alzheimer’s disease (part 1 of 2)
Source: EMBO J. 2024 Oct 18;43(23):6001–31. doi: 10.1038/s44318-024-00270-7 (PMC11612450; doi:10.1038/s44318-024-00270-7)

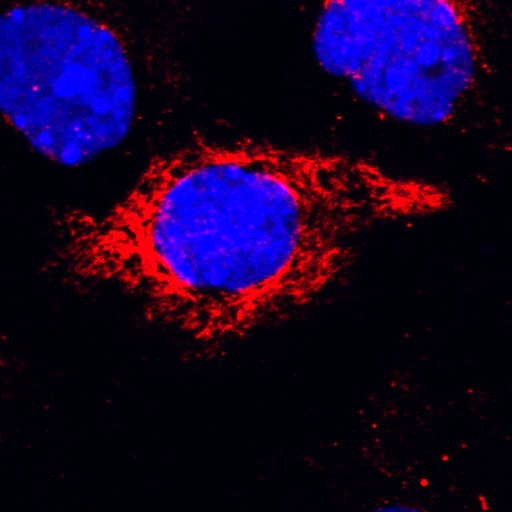

Supplement: Supplementary file 6 — Source data Fig. 1 [file 44318_2024_270_MOESM6_ESM.zip › Fig 1/1B/IF of ATP5a.tif]

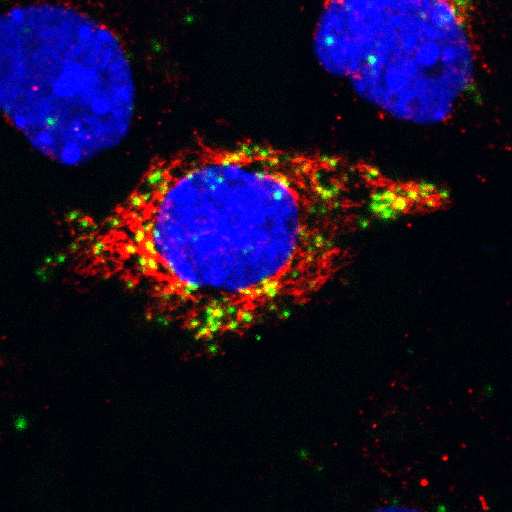

Supplement: Supplementary file 6 — Source data Fig. 1 [file 44318_2024_270_MOESM6_ESM.zip › Fig 1/1B/Merge.tif]

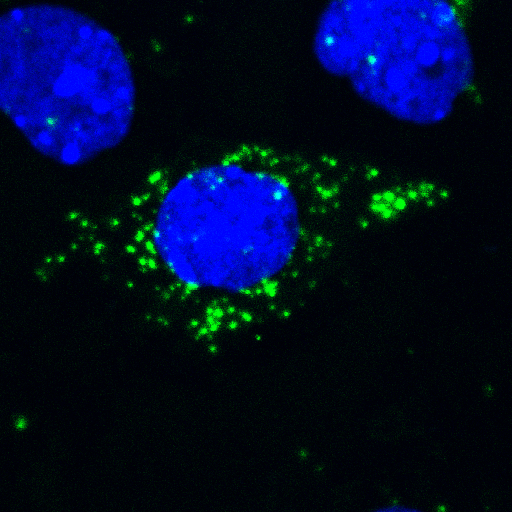

Supplement: Supplementary file 6 — Source data Fig. 1 [file 44318_2024_270_MOESM6_ESM.zip › Fig 1/1B/RNAscope of lncMtDloop.tif]

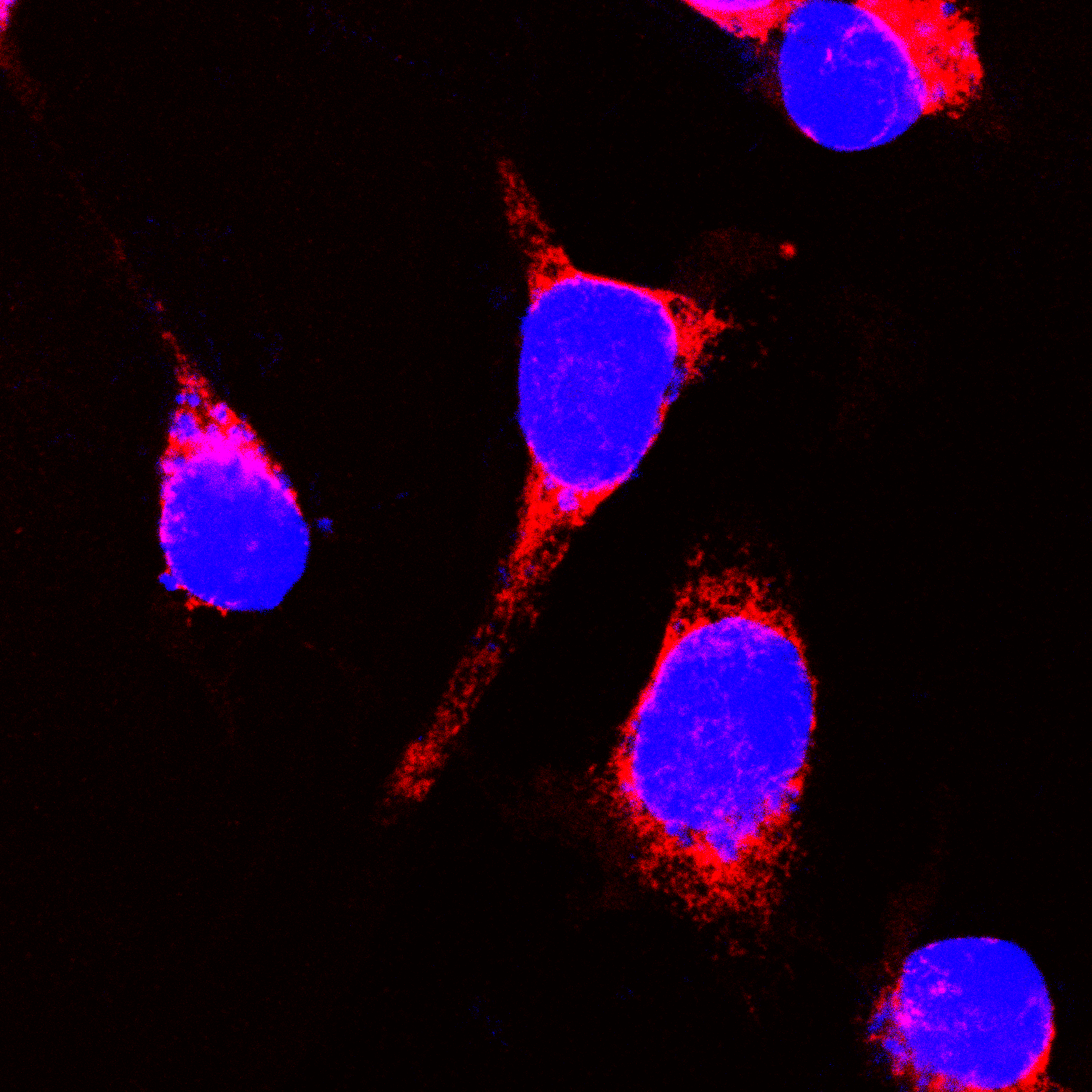

Supplement: Supplementary file 6 — Source data Fig. 1 [file 44318_2024_270_MOESM6_ESM.zip › Fig 1/1D/COS7-ATPa.tif]

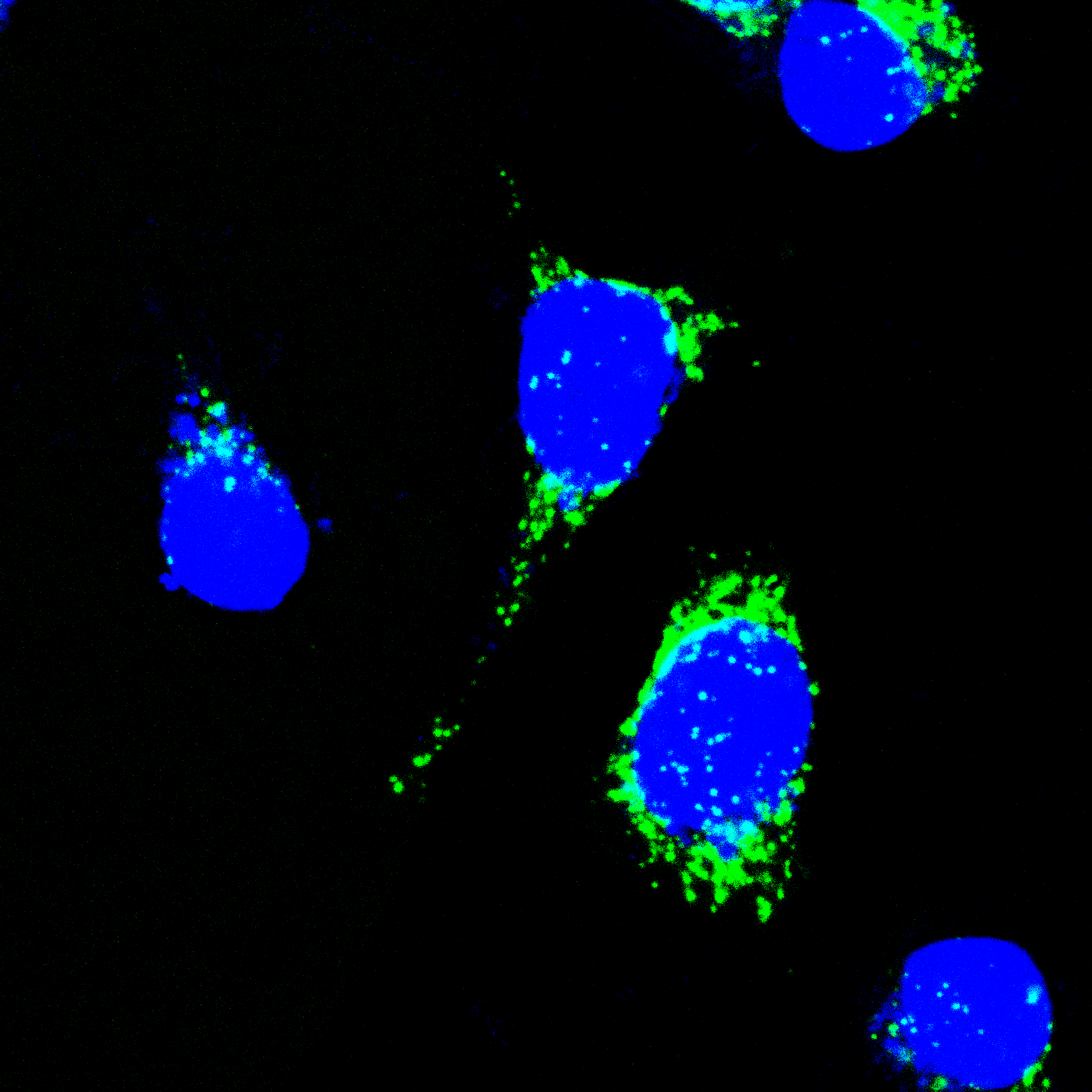

Supplement: Supplementary file 6 — Source data Fig. 1 [file 44318_2024_270_MOESM6_ESM.zip › Fig 1/1D/COS7-lncMtDloop.tif]

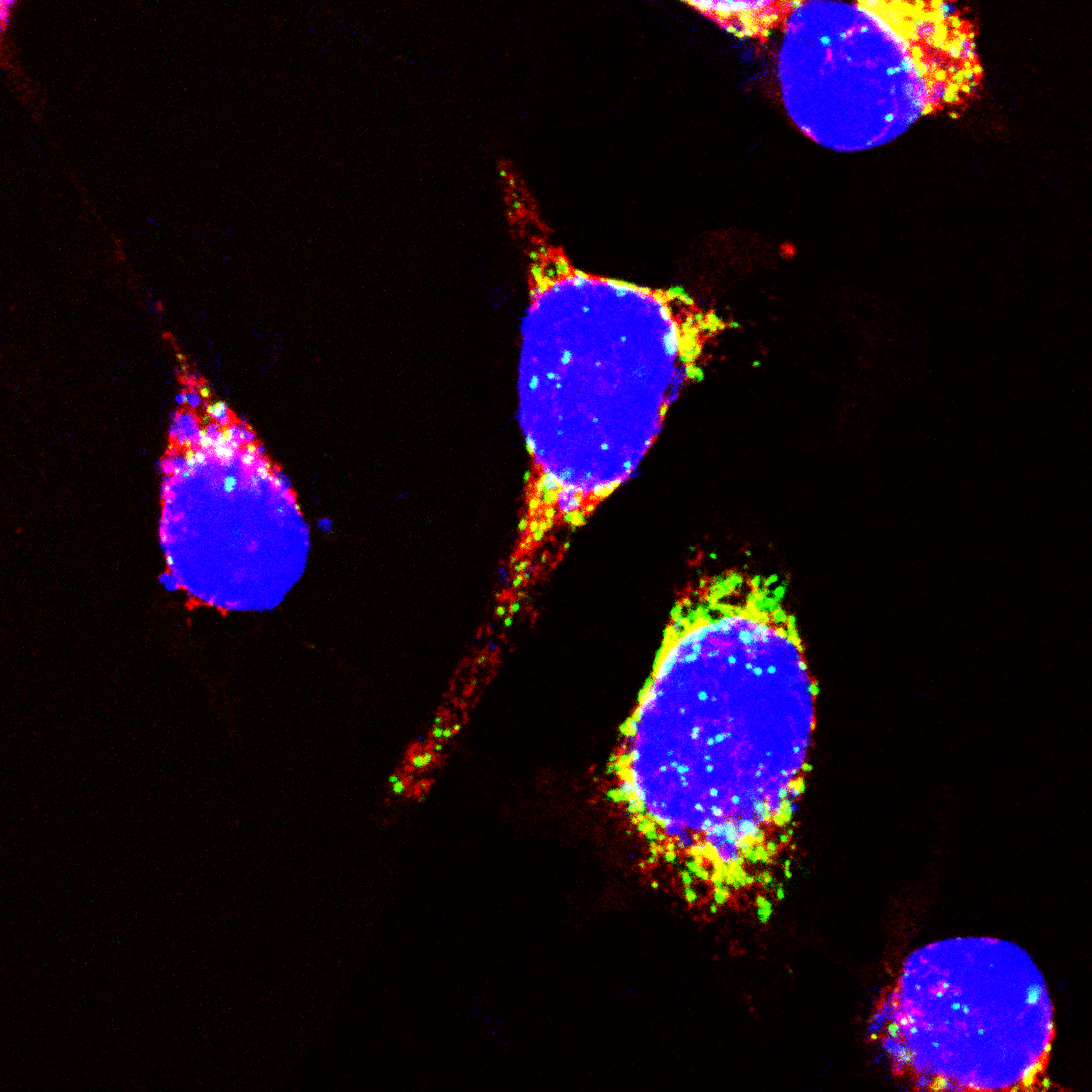

Supplement: Supplementary file 6 — Source data Fig. 1 [file 44318_2024_270_MOESM6_ESM.zip › Fig 1/1D/COS7-Merge.tif]

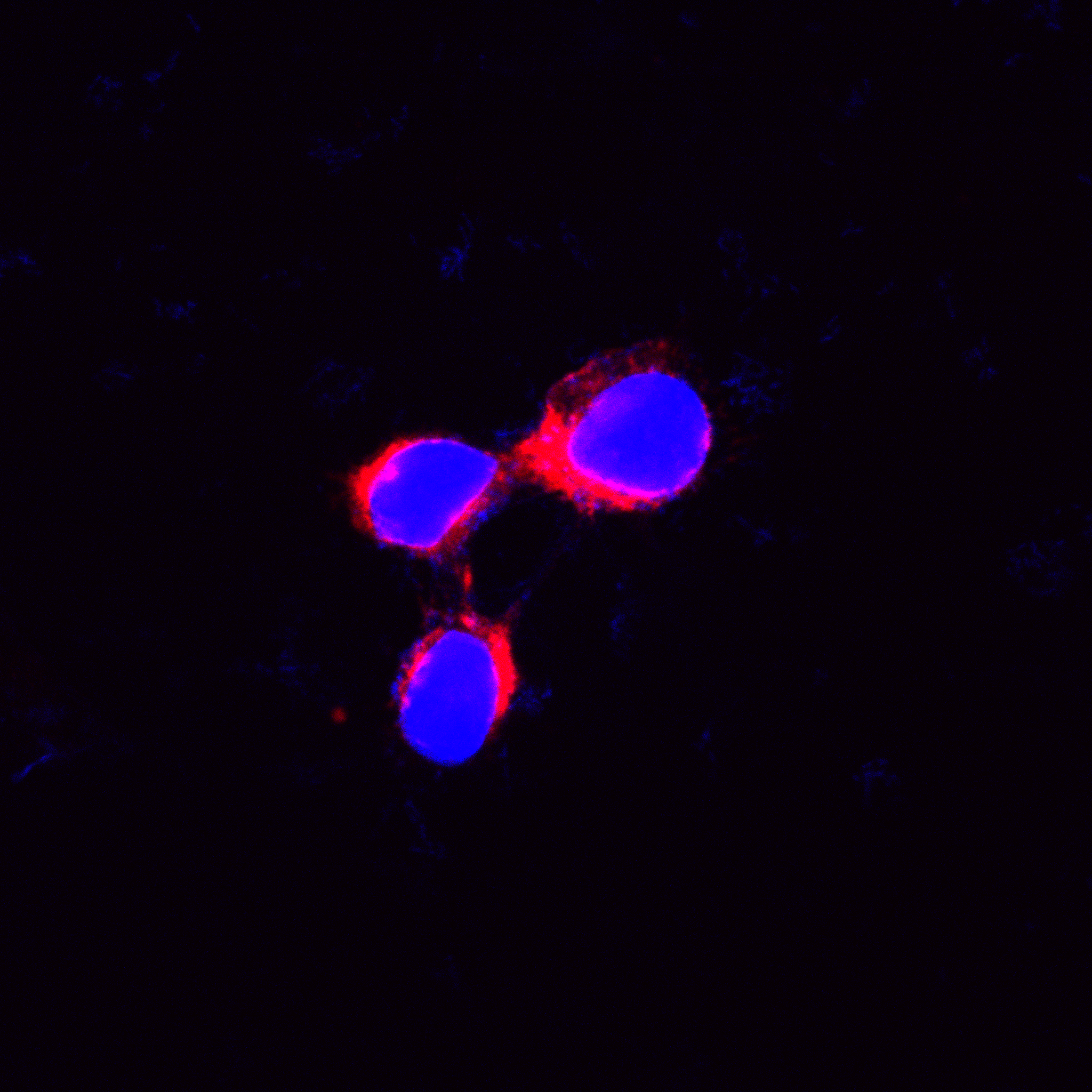

Supplement: Supplementary file 6 — Source data Fig. 1 [file 44318_2024_270_MOESM6_ESM.zip › Fig 1/1D/N2A-ATP5a.tif]

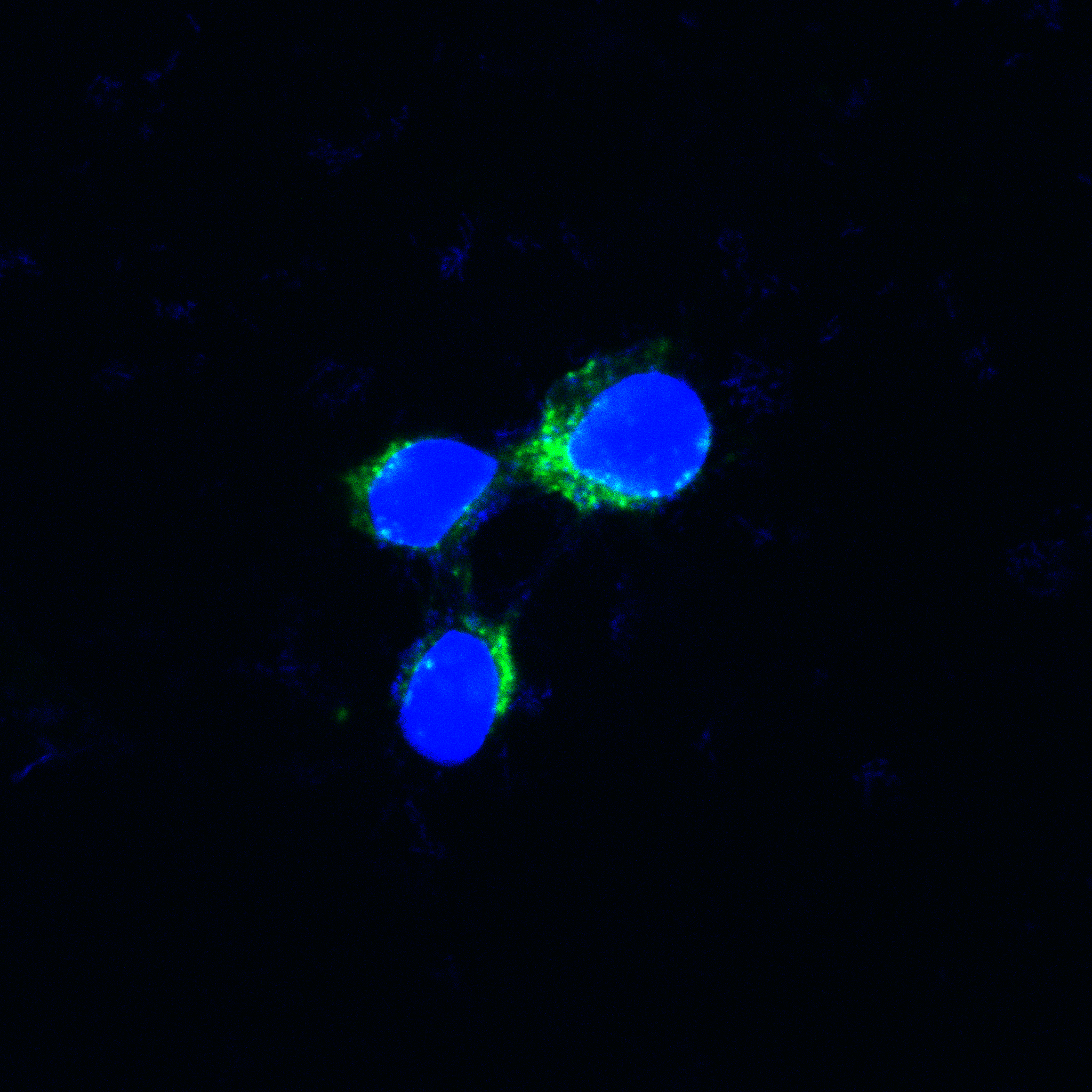

Supplement: Supplementary file 6 — Source data Fig. 1 [file 44318_2024_270_MOESM6_ESM.zip › Fig 1/1D/N2A-lncMtDloop.tif]

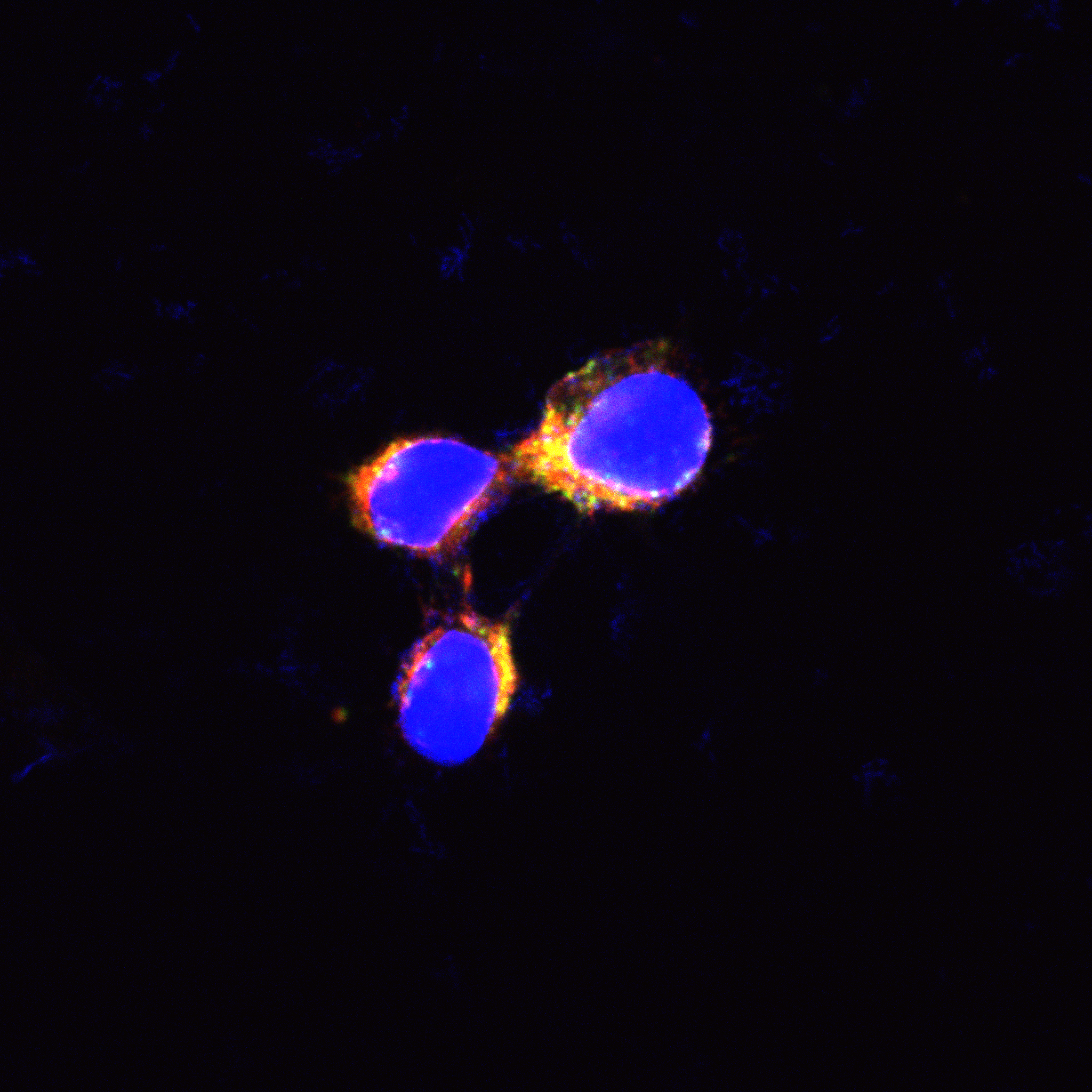

Supplement: Supplementary file 6 — Source data Fig. 1 [file 44318_2024_270_MOESM6_ESM.zip › Fig 1/1D/N2A-Merge.tif]

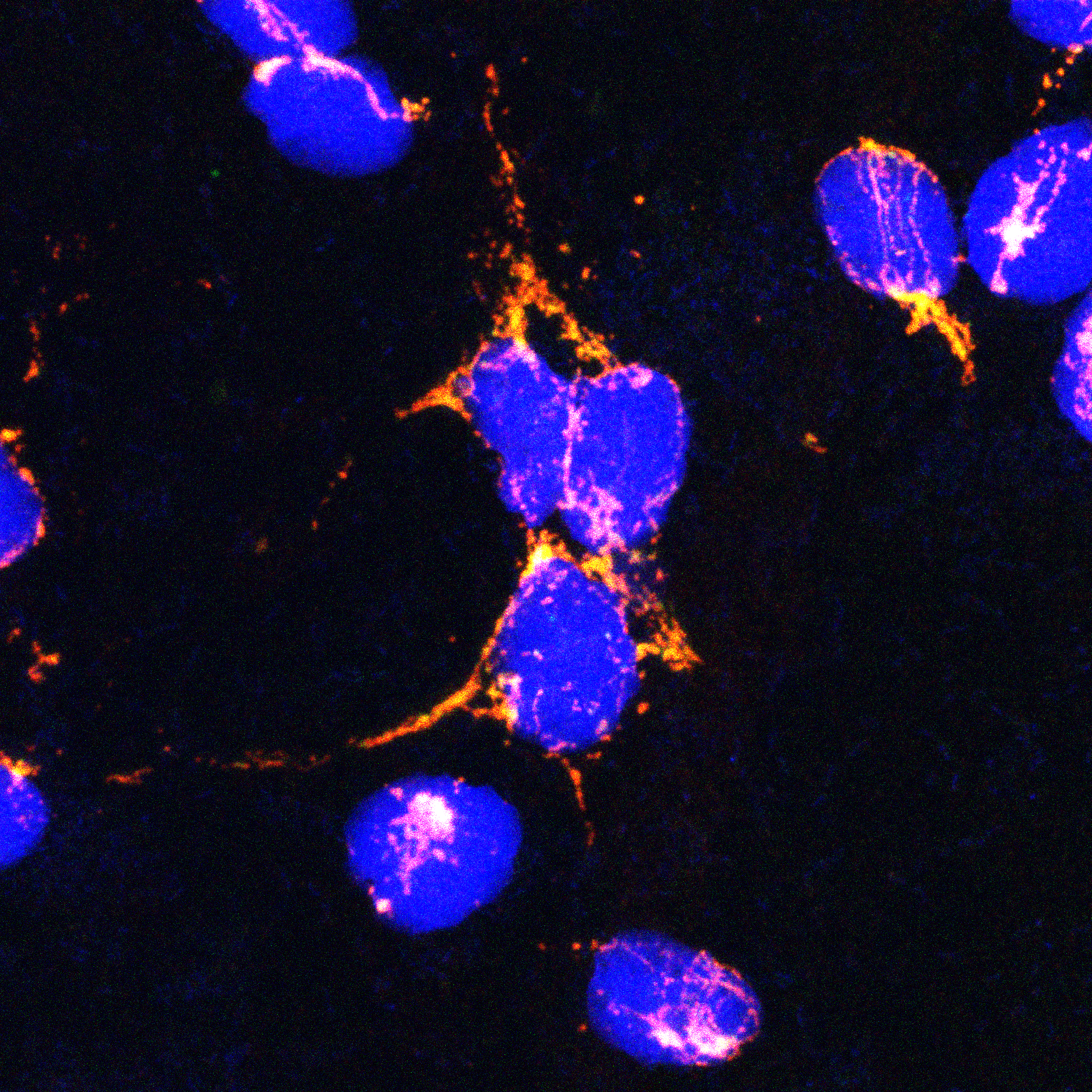

Supplement: Supplementary file 6 — Source data Fig. 1 [file 44318_2024_270_MOESM6_ESM.zip › Fig 1/1D/sy5y-co7_Series034.tif]

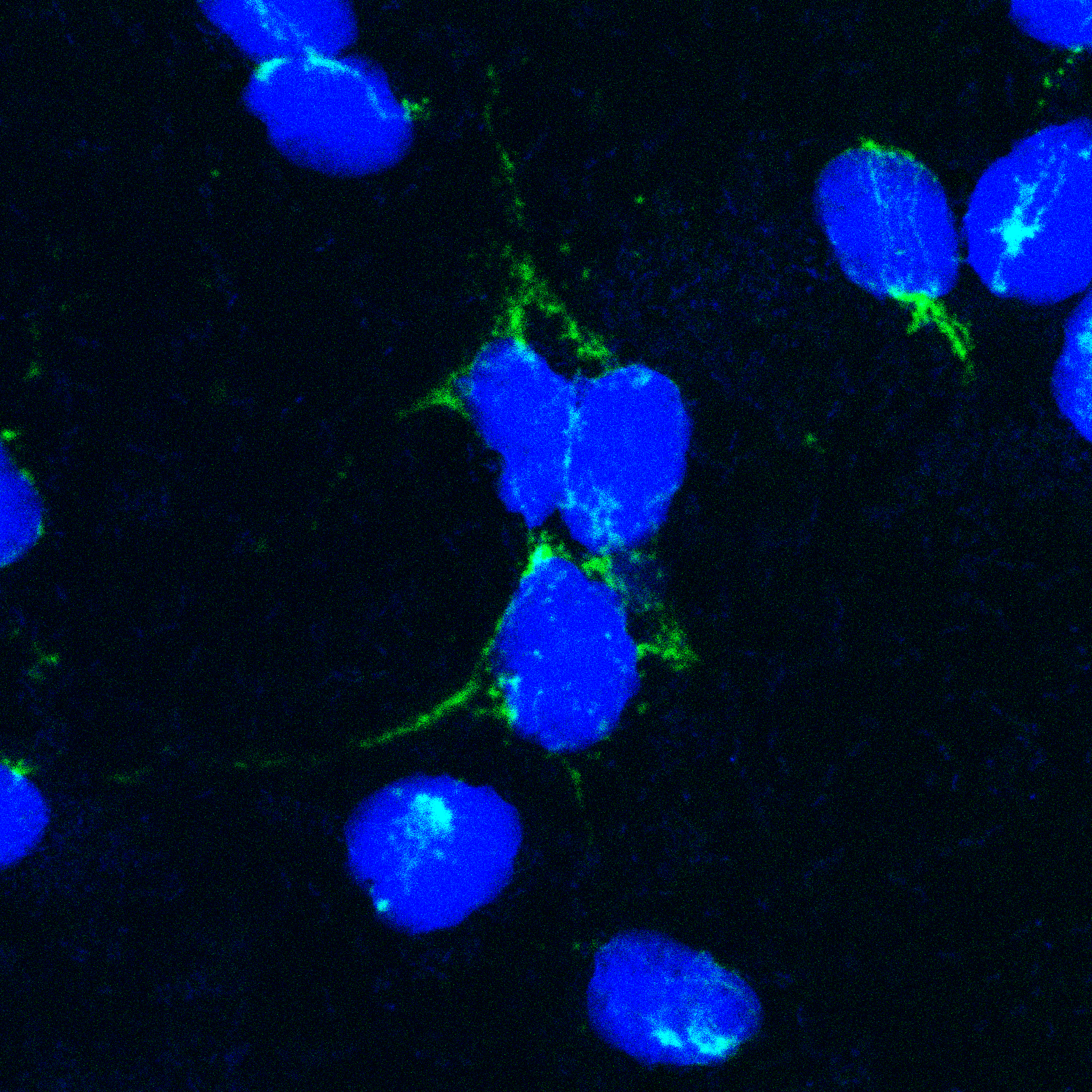

Supplement: Supplementary file 6 — Source data Fig. 1 [file 44318_2024_270_MOESM6_ESM.zip › Fig 1/1D/sy5y-lncMtDloop.tif]

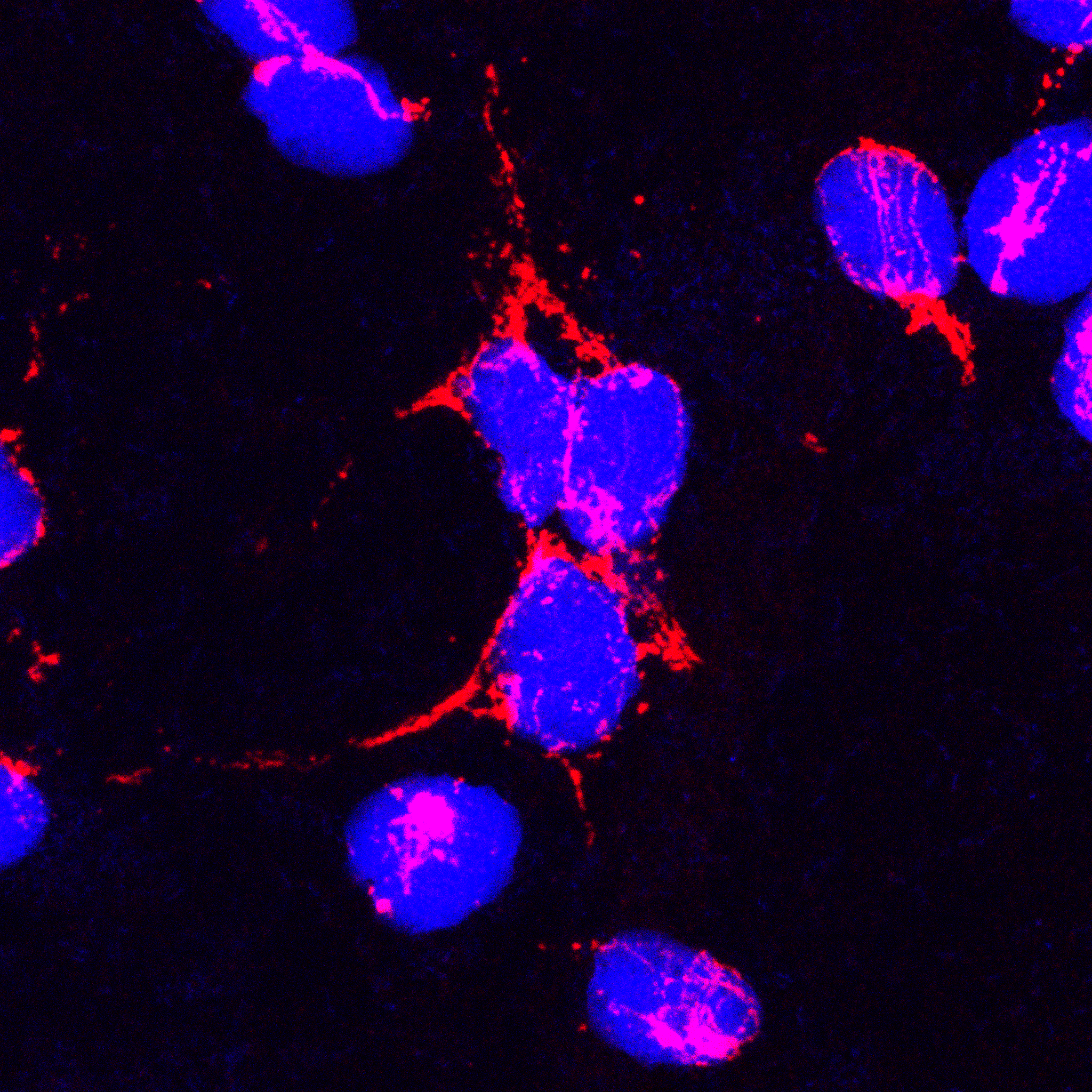

Supplement: Supplementary file 6 — Source data Fig. 1 [file 44318_2024_270_MOESM6_ESM.zip › Fig 1/1D/sy5y-Merge.tif]

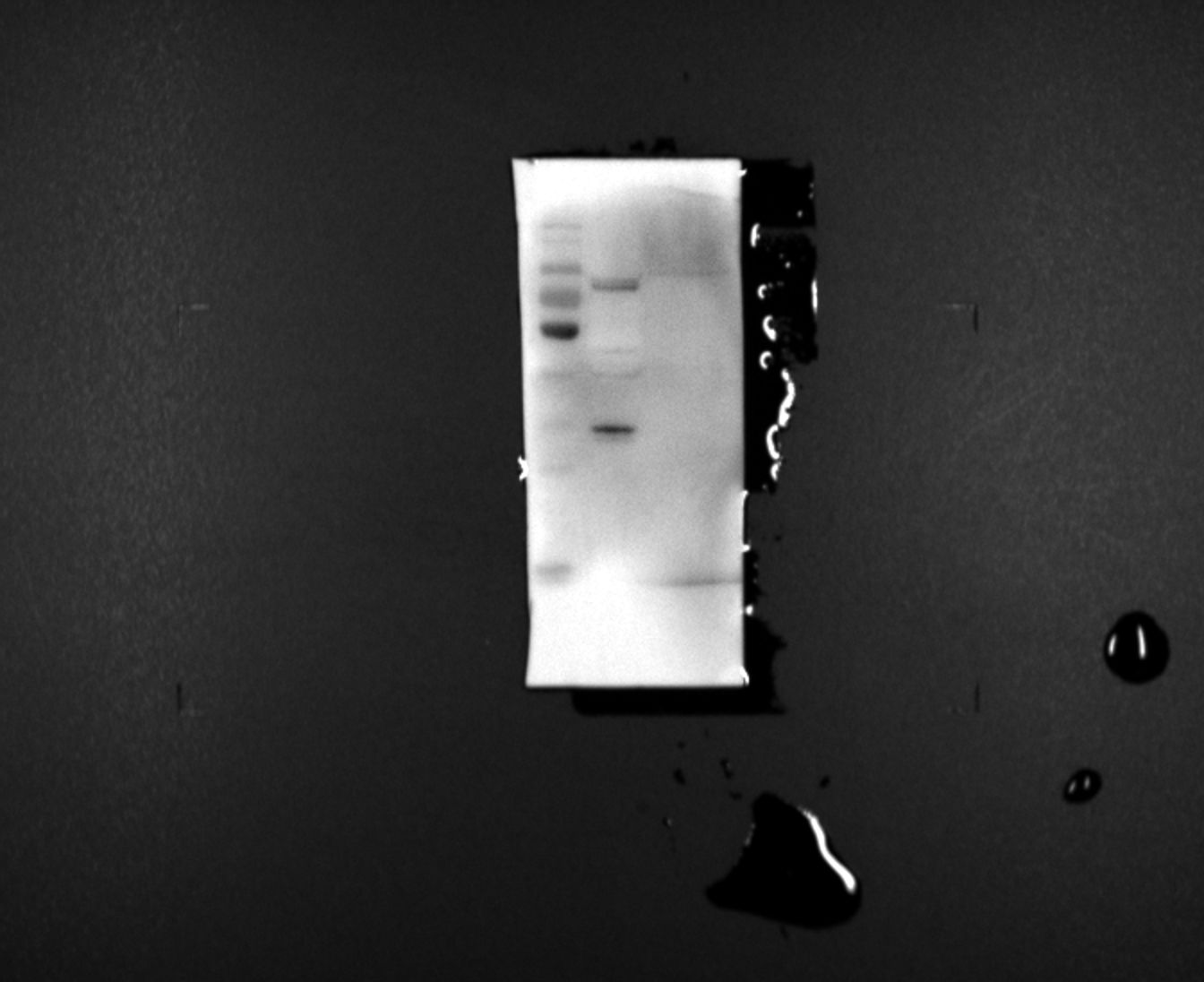

Supplement: Supplementary file 7 — Source data Fig. 2 [file 44318_2024_270_MOESM7_ESM.zip › Fig 2/Figure 2E/RNA pull-dowm-VDAC1.Tif]

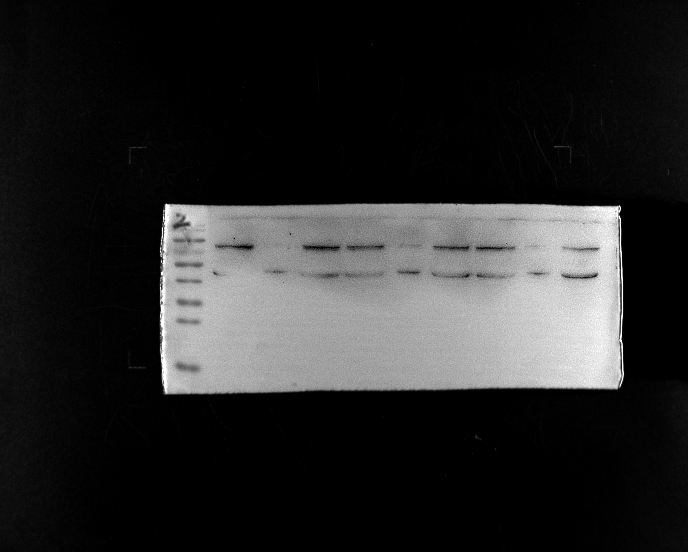

Supplement: Supplementary file 7 — Source data Fig. 2 [file 44318_2024_270_MOESM7_ESM.zip › Fig 2/Figure 2E/RNA pull-down-PNPASE.tif]

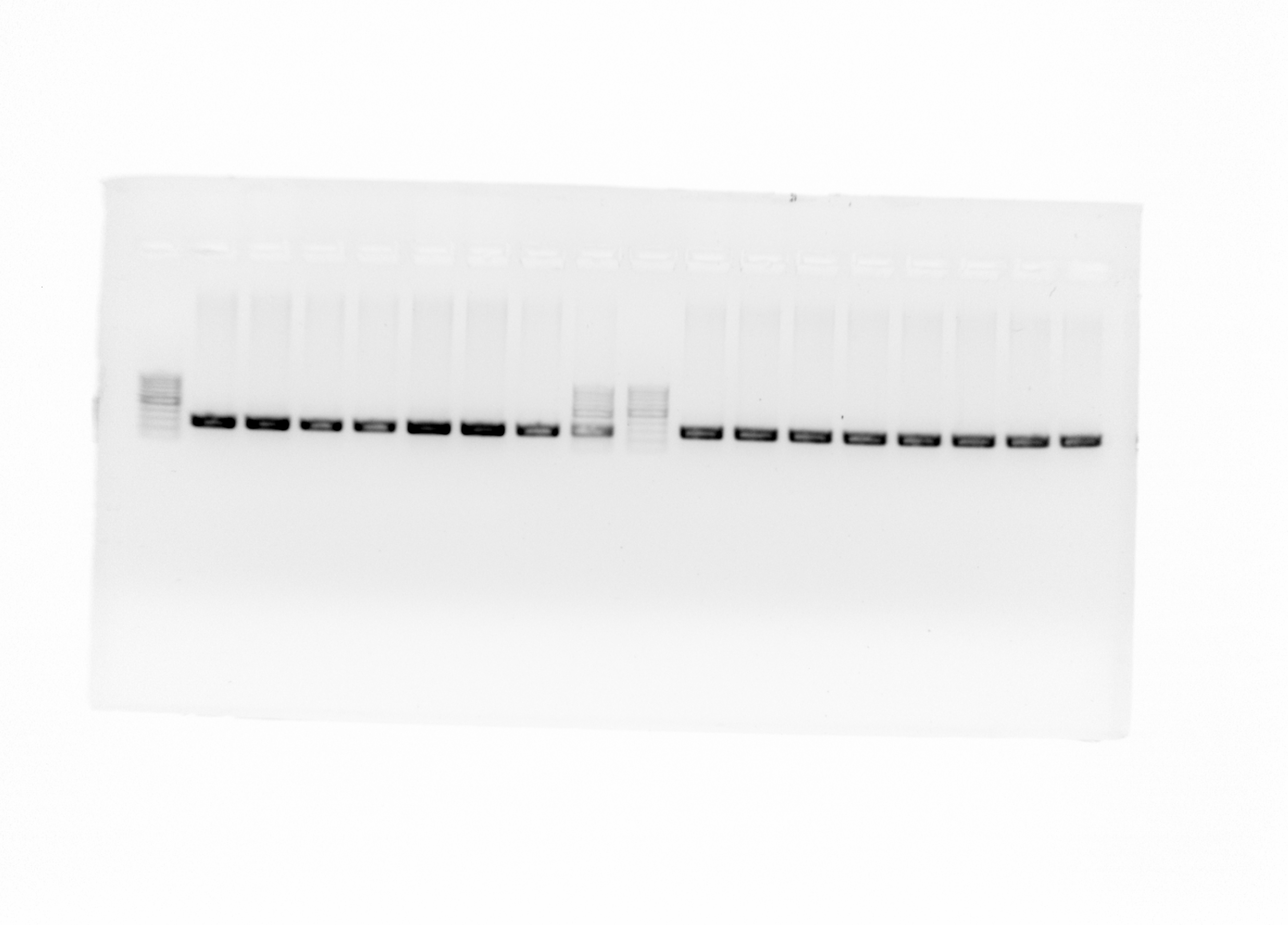

Supplement: Supplementary file 7 — Source data Fig. 2 [file 44318_2024_270_MOESM7_ESM.zip › Fig 2/Figure 2G/Northern blot-down lane.tif]

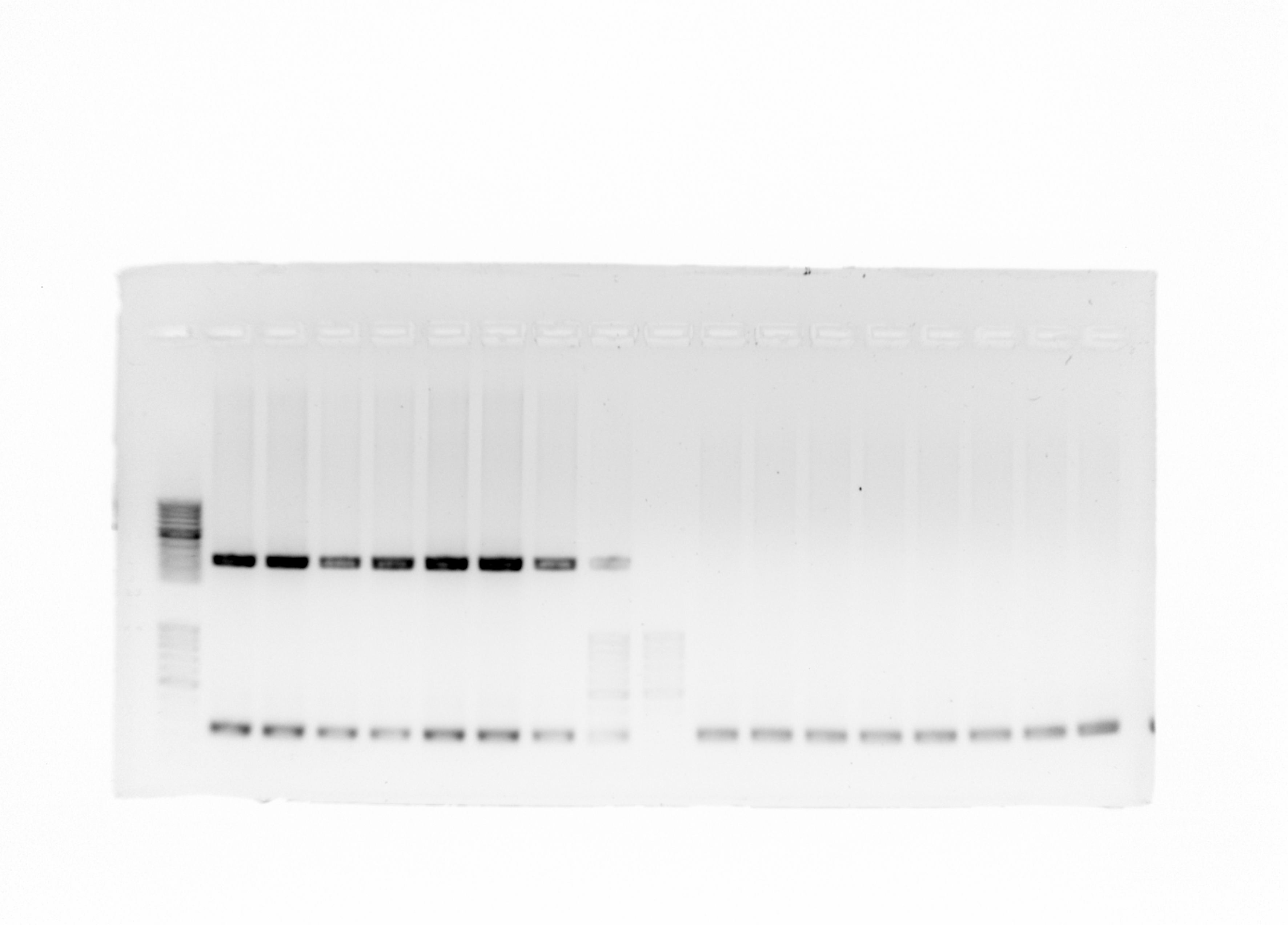

Supplement: Supplementary file 7 — Source data Fig. 2 [file 44318_2024_270_MOESM7_ESM.zip › Fig 2/Figure 2G/Northern blot-up lane.tif]

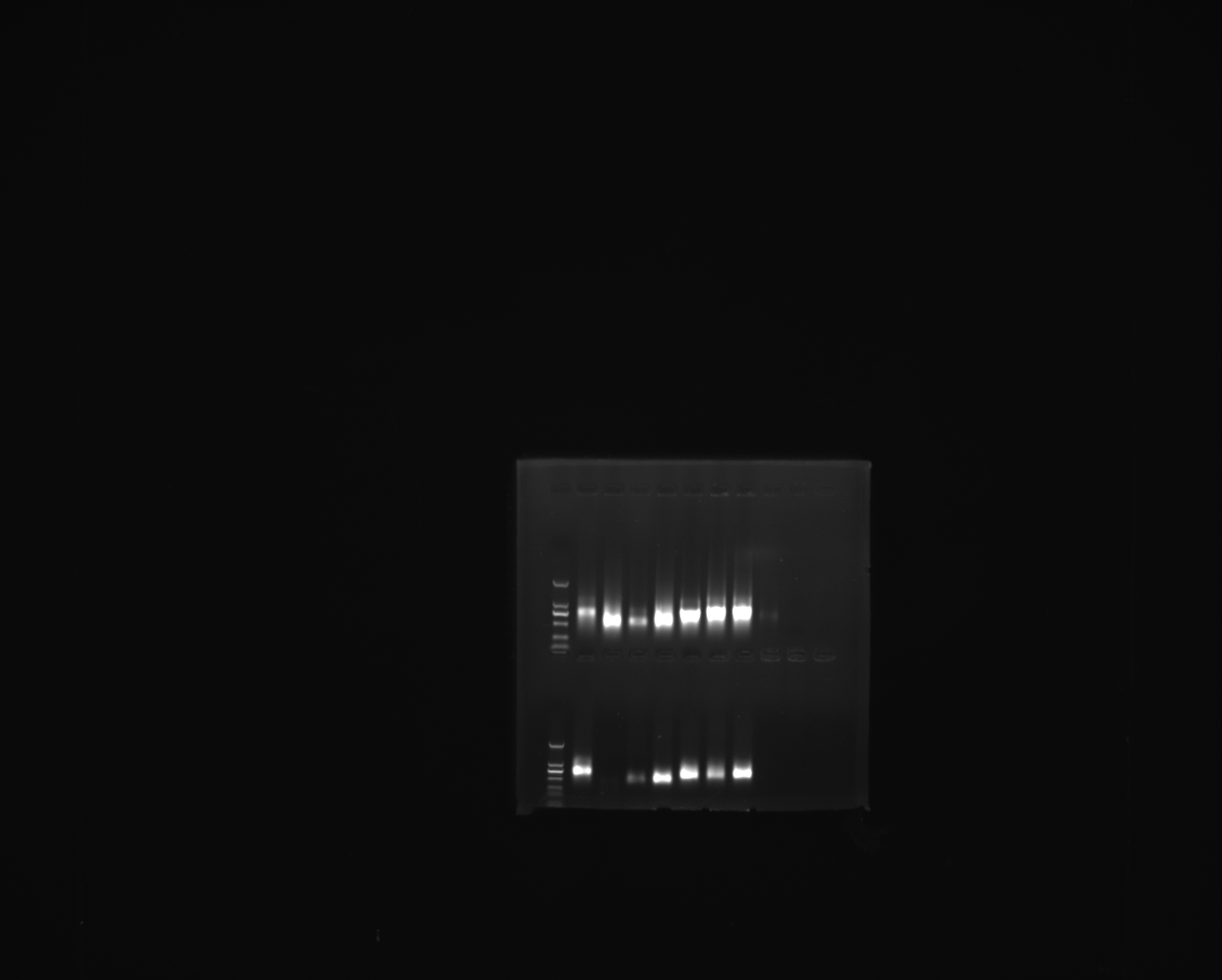

Supplement: Supplementary file 7 — Source data Fig. 2 [file 44318_2024_270_MOESM7_ESM.zip › Fig 2/Figure 2K/In vitro transcription of lncMtDloop.tif]

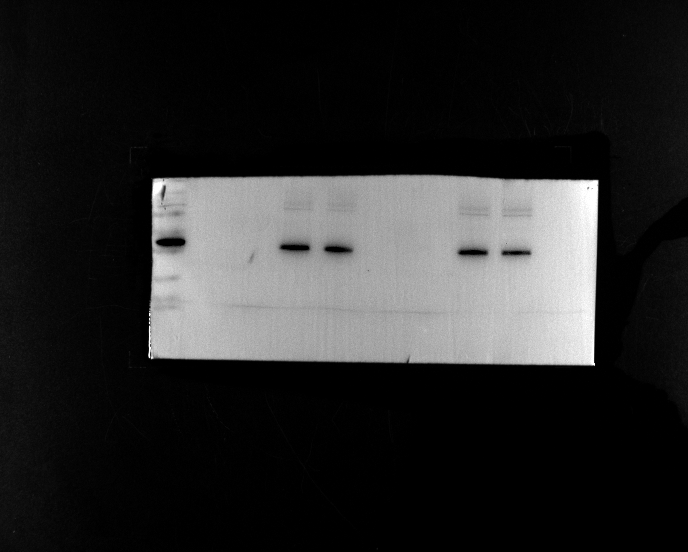

Supplement: Supplementary file 7 — Source data Fig. 2 [file 44318_2024_270_MOESM7_ESM.zip › Fig 2/Figure 2K/RNA pull down of truncted lncMtDloop.tif]

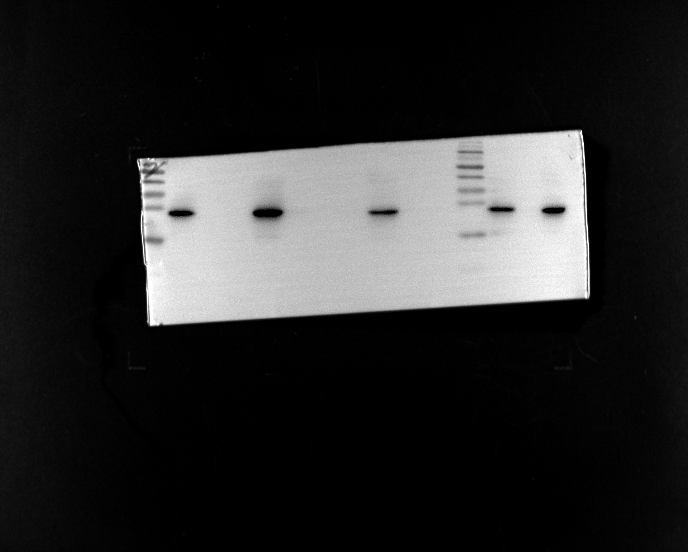

Supplement: Supplementary file 8 — Source data Fig. 3 [file 44318_2024_270_MOESM8_ESM.zip › Fig 3/3D/RNA pull down of TFAM.tif]

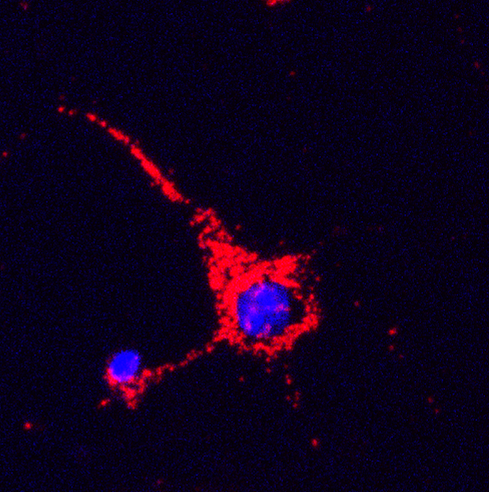

Supplement: Supplementary file 8 — Source data Fig. 3 [file 44318_2024_270_MOESM8_ESM.zip › Fig 3/3F/IF of TFAM.tif]

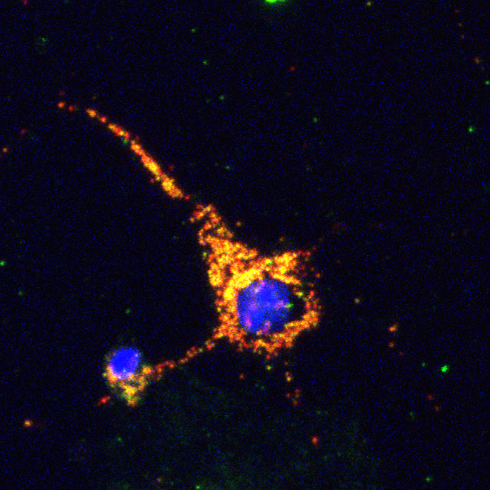

Supplement: Supplementary file 8 — Source data Fig. 3 [file 44318_2024_270_MOESM8_ESM.zip › Fig 3/3F/Merge.tif]

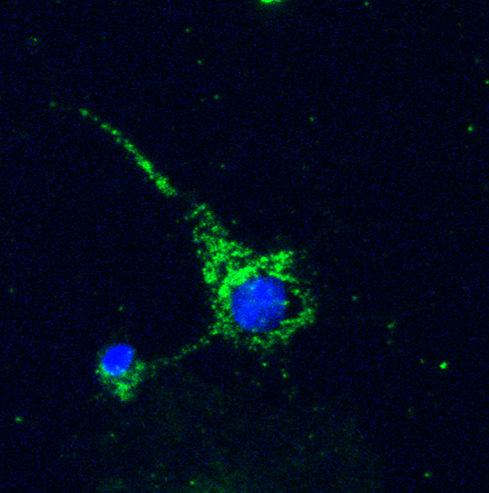

Supplement: Supplementary file 8 — Source data Fig. 3 [file 44318_2024_270_MOESM8_ESM.zip › Fig 3/3F/RNAscope of lncMtDloop.tif]

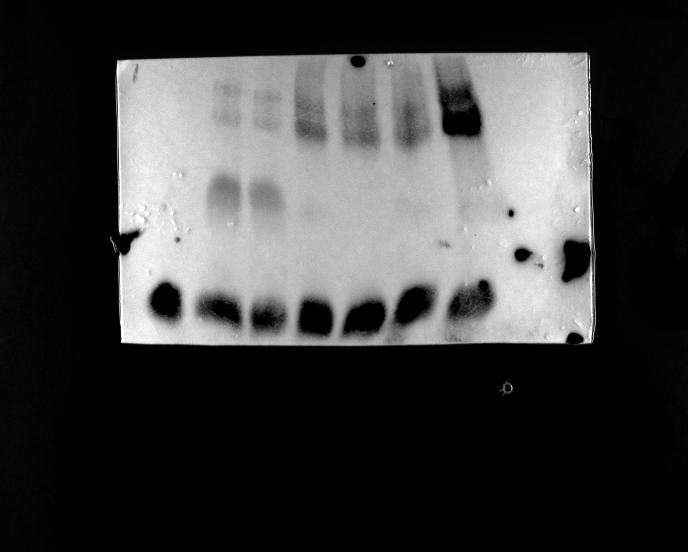

Supplement: Supplementary file 8 — Source data Fig. 3 [file 44318_2024_270_MOESM8_ESM.zip › Fig 3/3M/EMSA-TFAM.tif]

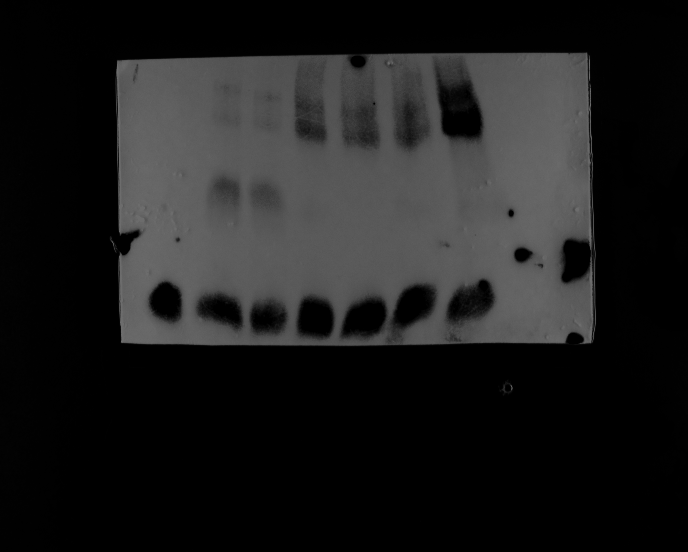

Supplement: Supplementary file 8 — Source data Fig. 3 [file 44318_2024_270_MOESM8_ESM.zip › Fig 3/3M/EMSA-TFAM1.tif]

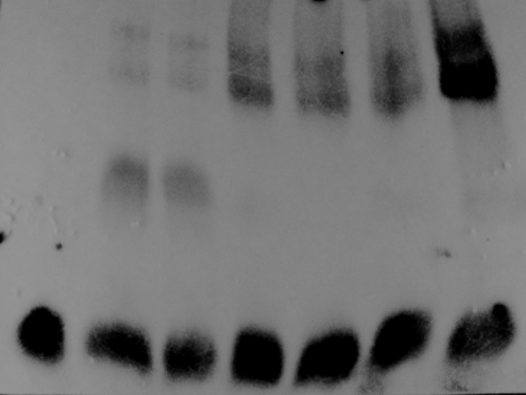

Supplement: Supplementary file 8 — Source data Fig. 3 [file 44318_2024_270_MOESM8_ESM.zip › Fig 3/3M/═╝╞1⁄410.tif]

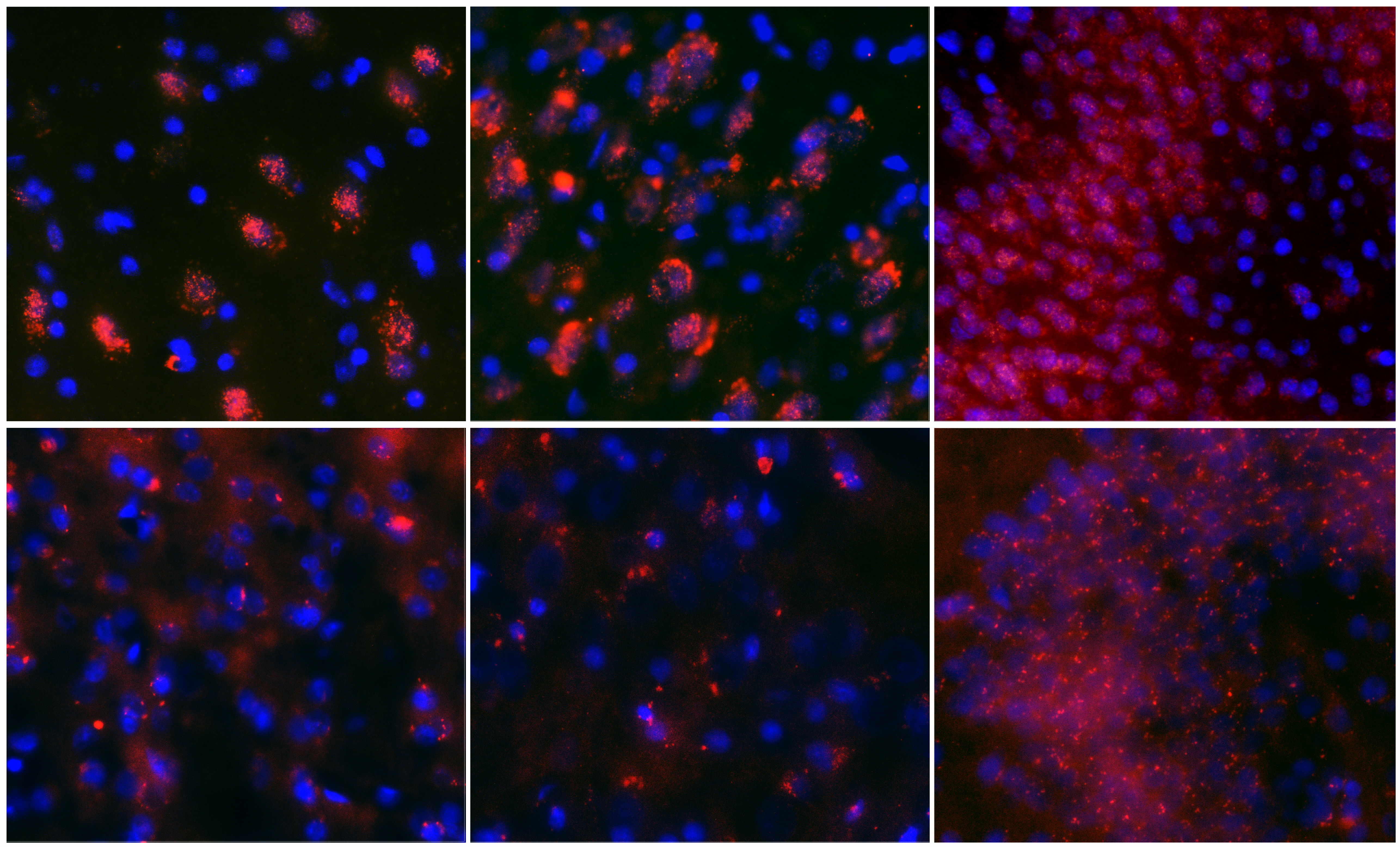

Supplement: Supplementary file 9 — Source data Fig. 4 [file 44318_2024_270_MOESM9_ESM.zip › Fig 4/Figure 4A/lncMtDloop_human.tif]

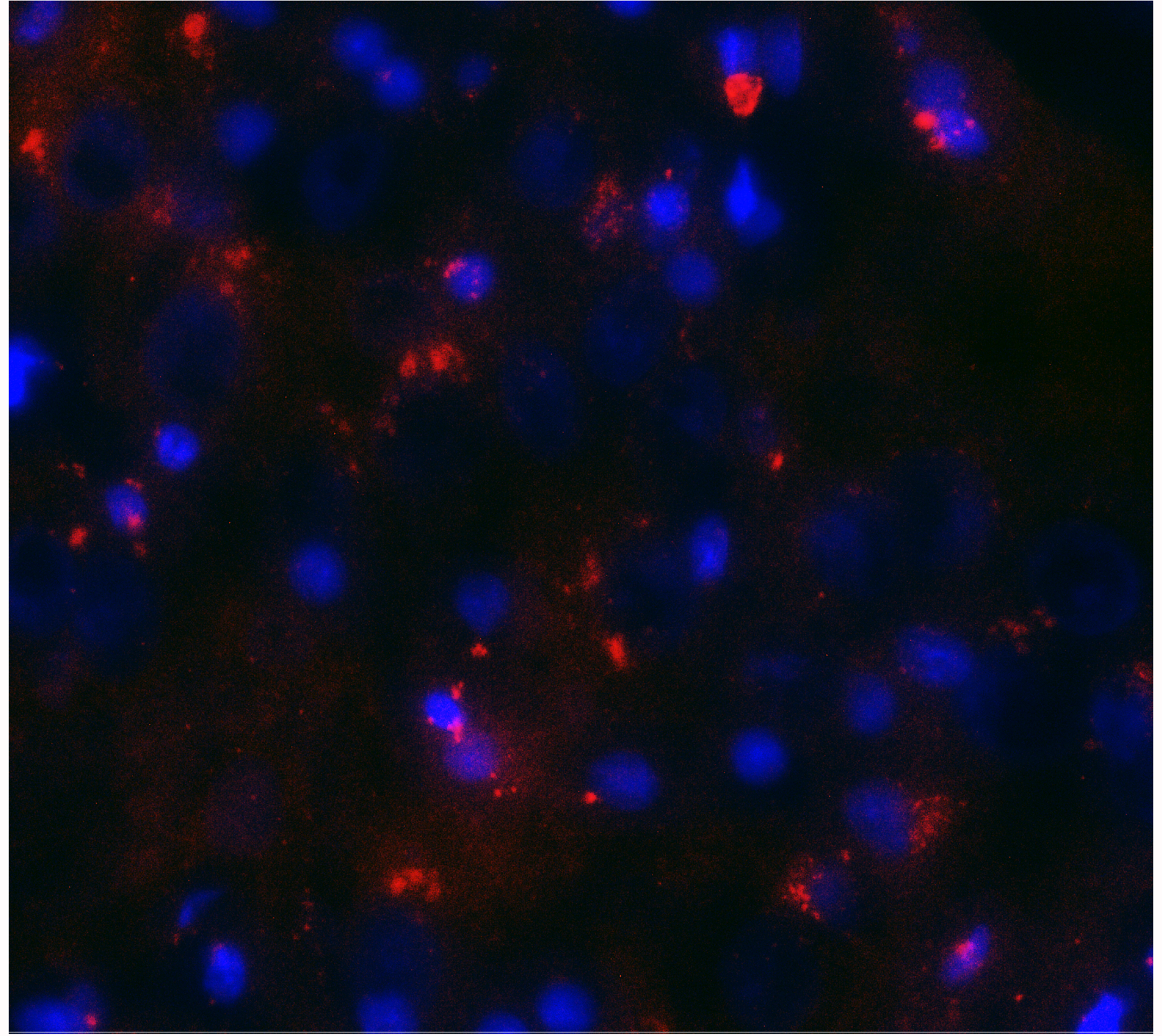

Supplement: Supplementary file 9 — Source data Fig. 4 [file 44318_2024_270_MOESM9_ESM.zip › Fig 4/Figure 4A/lncMtDloop_human_AD_CA1.tif]

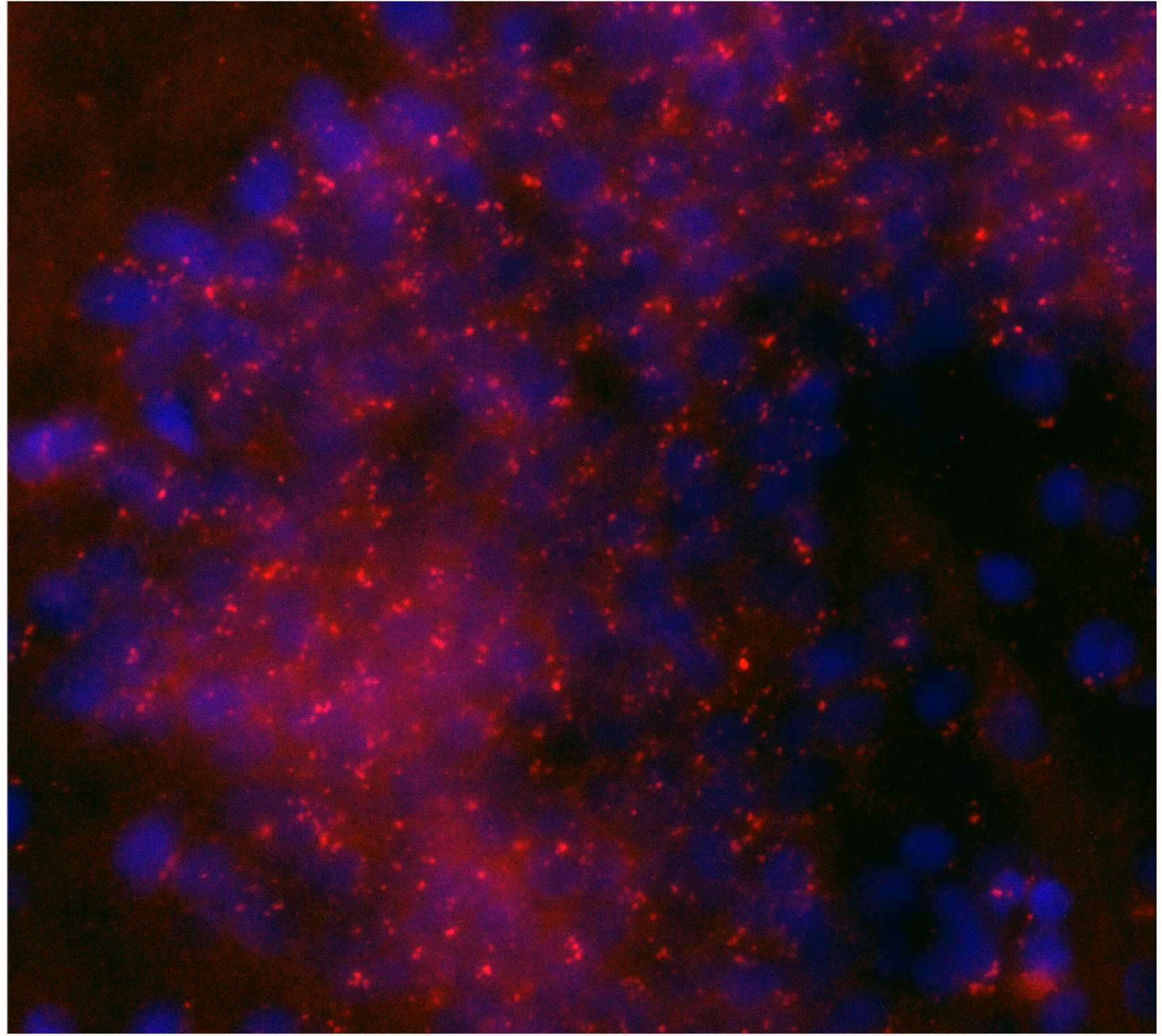

Supplement: Supplementary file 9 — Source data Fig. 4 [file 44318_2024_270_MOESM9_ESM.zip › Fig 4/Figure 4A/lncMtDloop_human_AD_DG.tif]

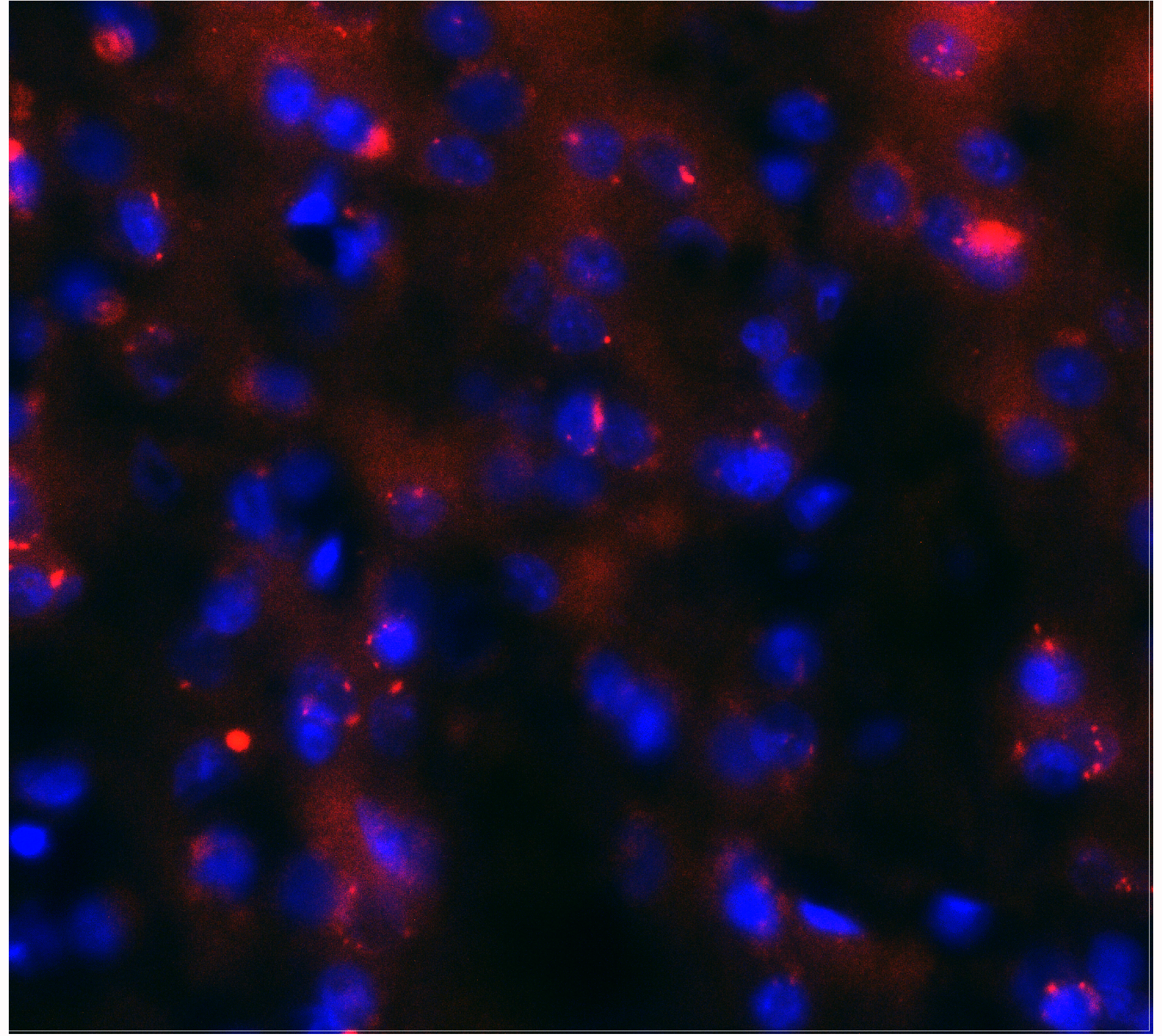

Supplement: Supplementary file 9 — Source data Fig. 4 [file 44318_2024_270_MOESM9_ESM.zip › Fig 4/Figure 4A/lncMtDloop_human_AD_PFC.tif]

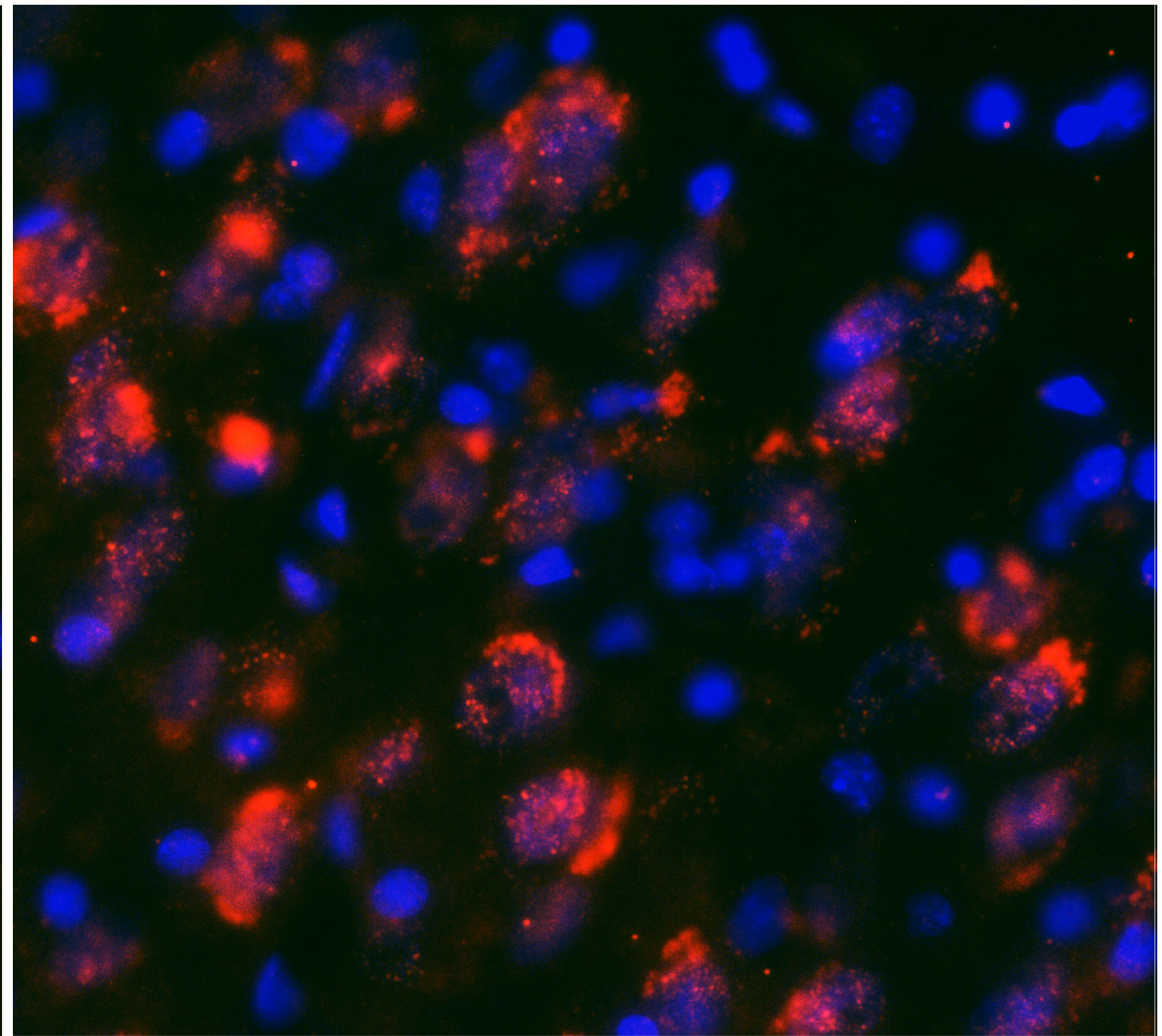

Supplement: Supplementary file 9 — Source data Fig. 4 [file 44318_2024_270_MOESM9_ESM.zip › Fig 4/Figure 4A/lncMtDloop_human_control_CA1.tif]

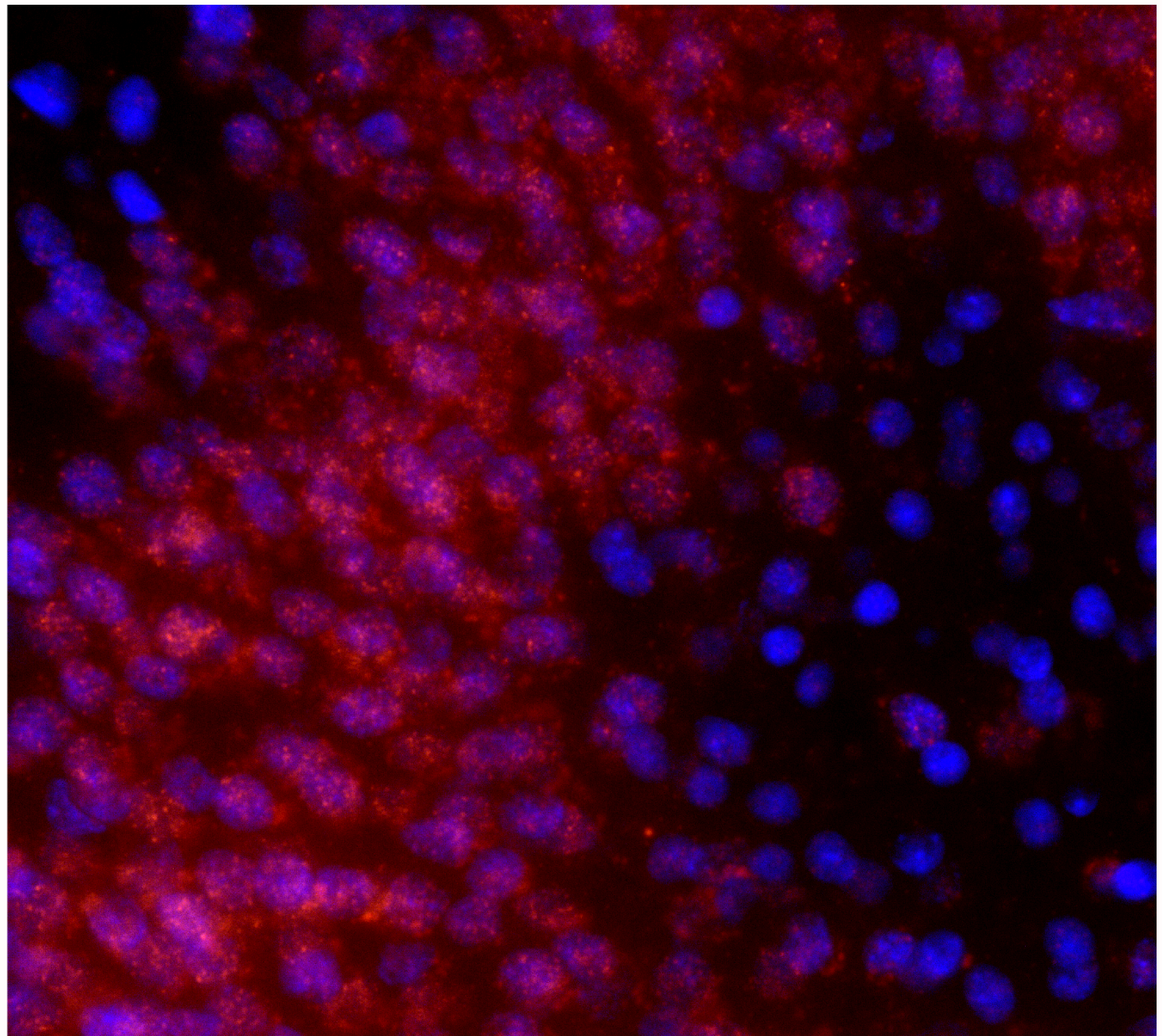

Supplement: Supplementary file 9 — Source data Fig. 4 [file 44318_2024_270_MOESM9_ESM.zip › Fig 4/Figure 4A/lncMtDloop_human_control_DG.tif]

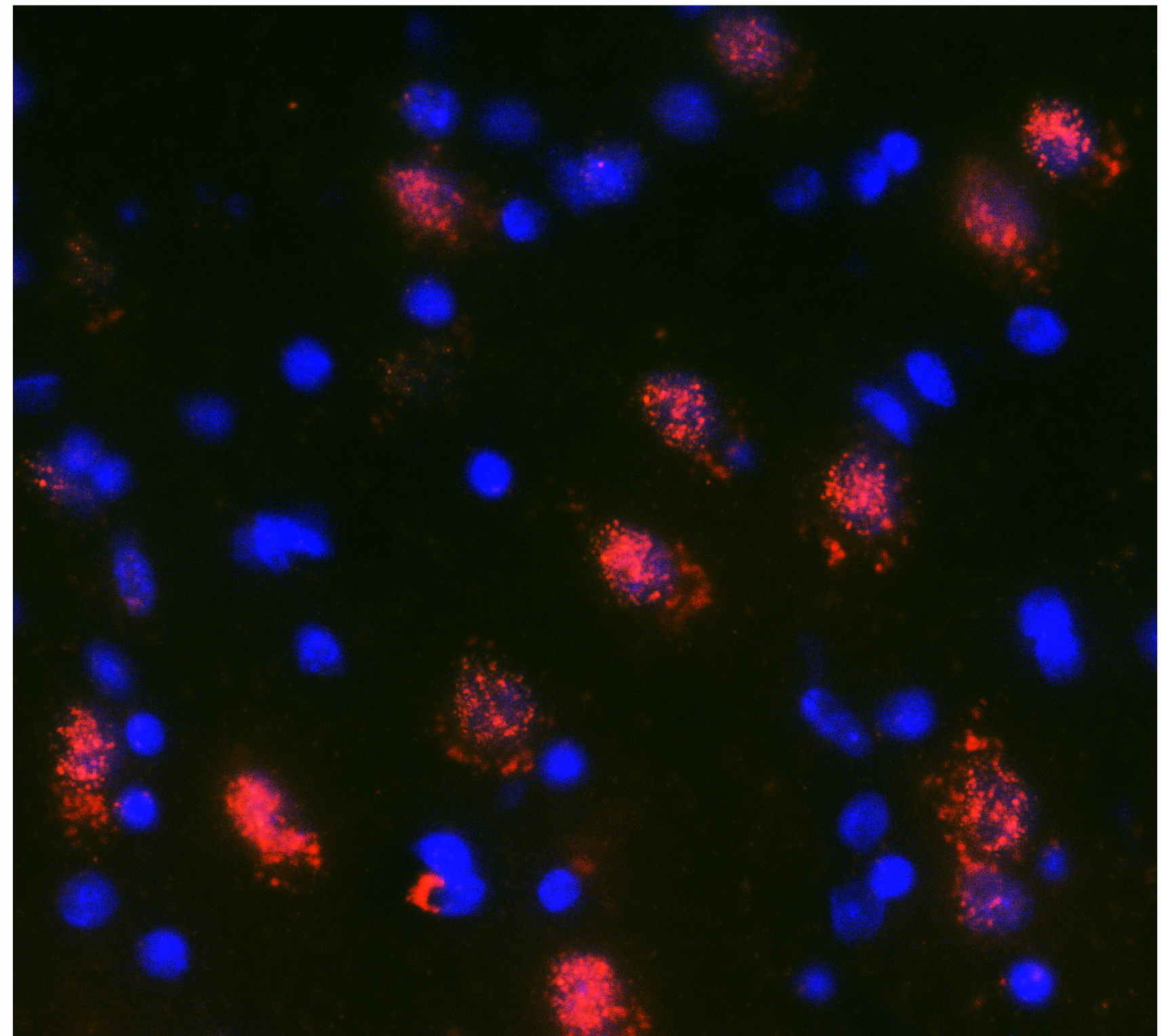

Supplement: Supplementary file 9 — Source data Fig. 4 [file 44318_2024_270_MOESM9_ESM.zip › Fig 4/Figure 4A/lncMtDloop_human_control_PFC.tif]

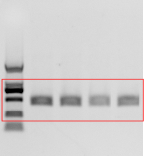

Supplement: Supplementary file 9 — Source data Fig. 4 [file 44318_2024_270_MOESM9_ESM.zip › Fig 4/Figure 4C/Actin_nb-human.tif]

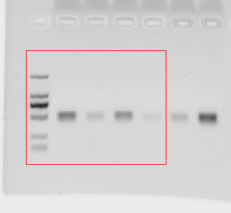

Supplement: Supplementary file 9 — Source data Fig. 4 [file 44318_2024_270_MOESM9_ESM.zip › Fig 4/Figure 4C/lncMtDloop_northern blot_human.tif]

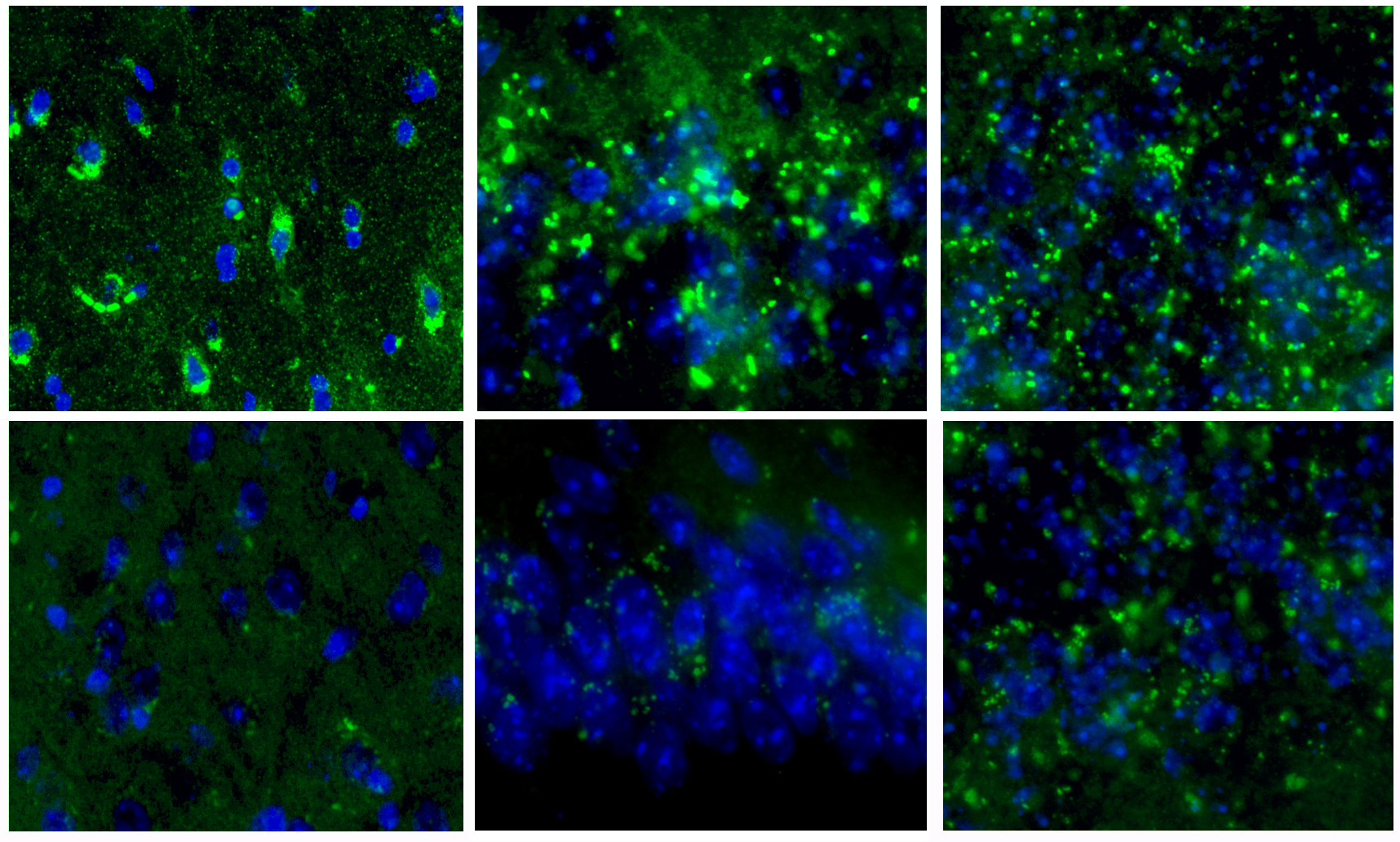

Supplement: Supplementary file 9 — Source data Fig. 4 [file 44318_2024_270_MOESM9_ESM.zip › Fig 4/Figure 4F/Fig 4F.tif]

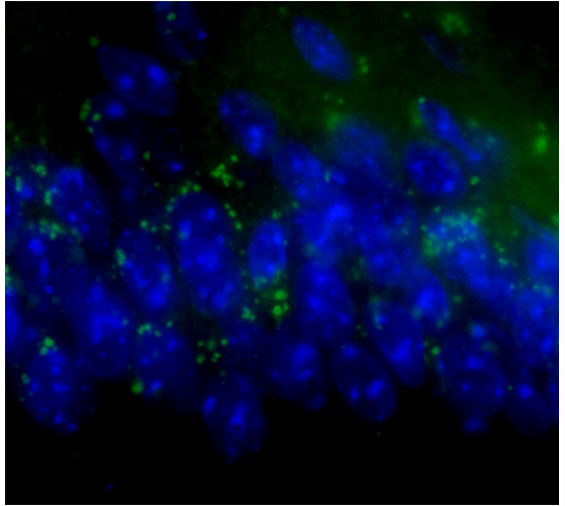

Supplement: Supplementary file 9 — Source data Fig. 4 [file 44318_2024_270_MOESM9_ESM.zip › Fig 4/Figure 4F/lncMtDloop_mouse_3xTg_CA1.tif]

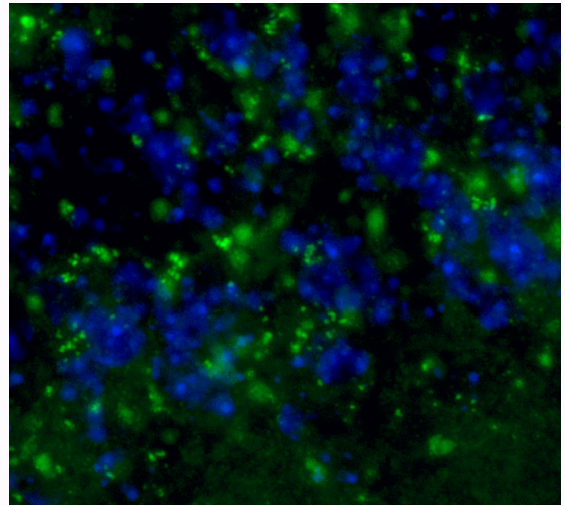

Supplement: Supplementary file 9 — Source data Fig. 4 [file 44318_2024_270_MOESM9_ESM.zip › Fig 4/Figure 4F/lncMtDloop_mouse_3xTg_DG.tif]

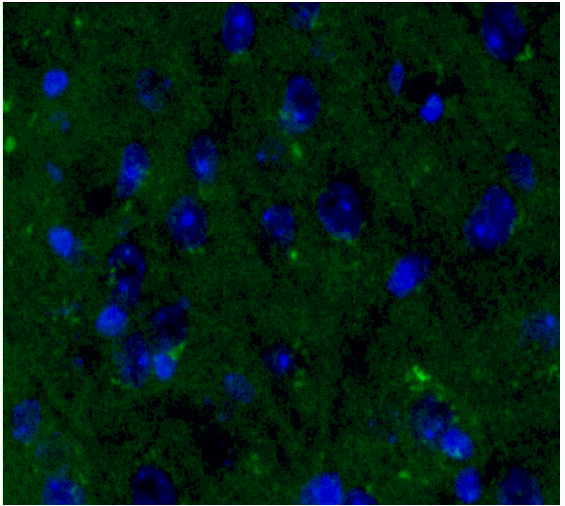

Supplement: Supplementary file 9 — Source data Fig. 4 [file 44318_2024_270_MOESM9_ESM.zip › Fig 4/Figure 4F/lncMtDloop_mouse_3xTg_PFC.tif]

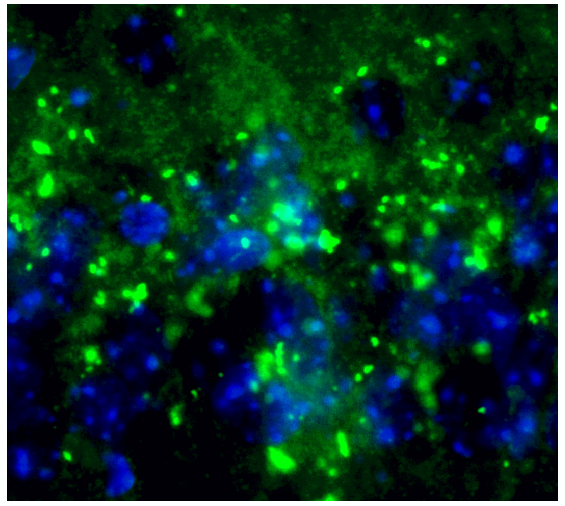

Supplement: Supplementary file 9 — Source data Fig. 4 [file 44318_2024_270_MOESM9_ESM.zip › Fig 4/Figure 4F/lncMtDloop_mouse_WT_CA1.tif]

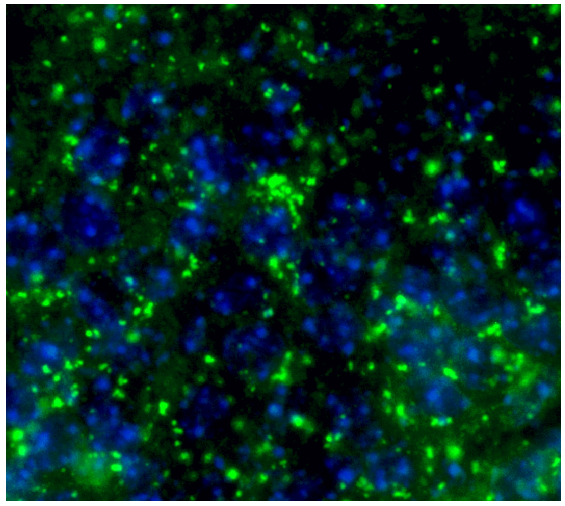

Supplement: Supplementary file 9 — Source data Fig. 4 [file 44318_2024_270_MOESM9_ESM.zip › Fig 4/Figure 4F/lncMtDloop_mouse_WT_DG.tif]

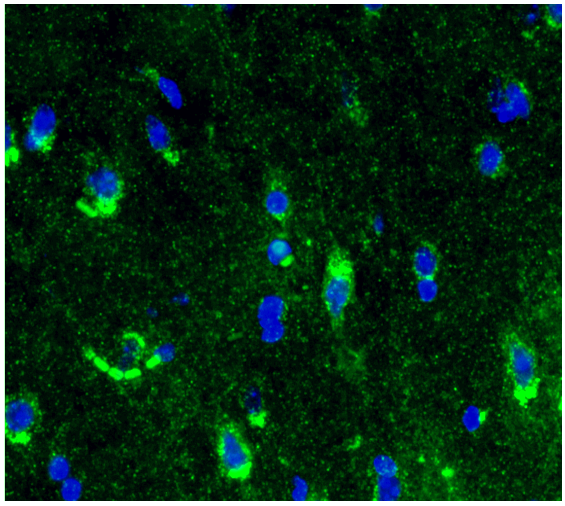

Supplement: Supplementary file 9 — Source data Fig. 4 [file 44318_2024_270_MOESM9_ESM.zip › Fig 4/Figure 4F/lncMtDloop_mouse_WT_PFC.tif]

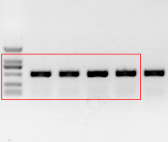

Supplement: Supplementary file 9 — Source data Fig. 4 [file 44318_2024_270_MOESM9_ESM.zip › Fig 4/Figure 4H/Actin_nb_mouse.tif]

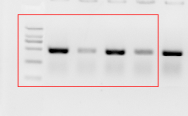

Supplement: Supplementary file 9 — Source data Fig. 4 [file 44318_2024_270_MOESM9_ESM.zip › Fig 4/Figure 4H/lncMtDloop_nb-mouse.tif]

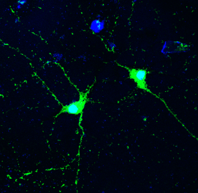

Supplement: Supplementary file 9 — Source data Fig. 4 [file 44318_2024_270_MOESM9_ESM.zip › Fig 4/Figure 4K/IF-MAP2-3xTg.png]

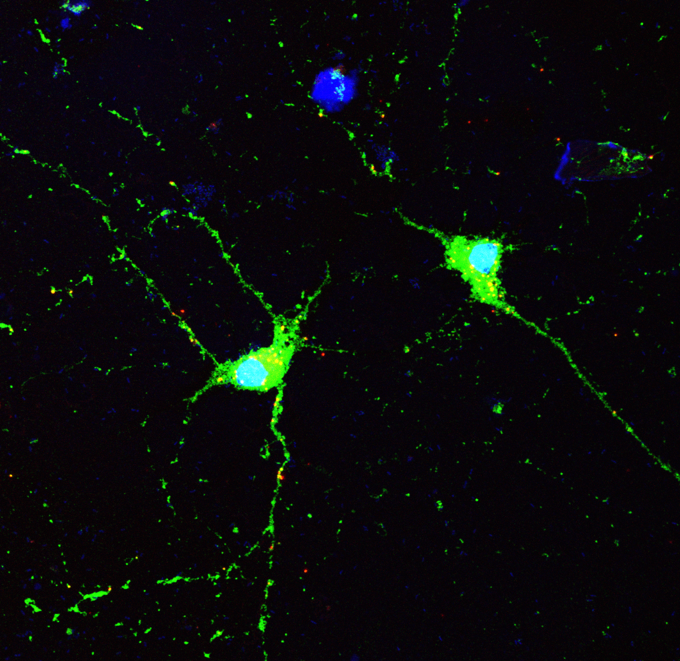

Supplement: Supplementary file 9 — Source data Fig. 4 [file 44318_2024_270_MOESM9_ESM.zip › Fig 4/Figure 4K/IF-MAP2-3xTg-Merge.tif]

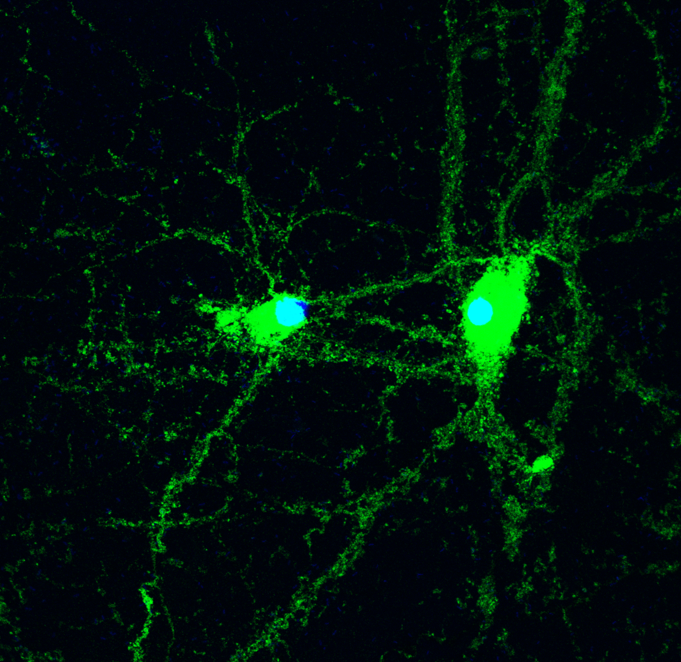

Supplement: Supplementary file 9 — Source data Fig. 4 [file 44318_2024_270_MOESM9_ESM.zip › Fig 4/Figure 4K/IF-MAP2-WT.tif]

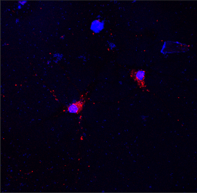

Supplement: Supplementary file 9 — Source data Fig. 4 [file 44318_2024_270_MOESM9_ESM.zip › Fig 4/Figure 4K/RNAscope-lncMtDloop-3xTg.png]

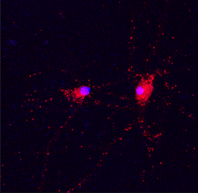

Supplement: Supplementary file 9 — Source data Fig. 4 [file 44318_2024_270_MOESM9_ESM.zip › Fig 4/Figure 4K/RNAscope-lncMtDloop-WT.png]

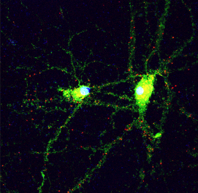

Supplement: Supplementary file 9 — Source data Fig. 4 [file 44318_2024_270_MOESM9_ESM.zip › Fig 4/Figure 4K/RNAscope-lncMtDloop-WT-MERGE.png]

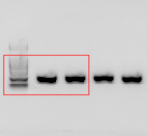

Supplement: Supplementary file 9 — Source data Fig. 4 [file 44318_2024_270_MOESM9_ESM.zip › Fig 4/Figure 4M/Actin_nb_m.tif]

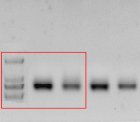

Supplement: Supplementary file 9 — Source data Fig. 4 [file 44318_2024_270_MOESM9_ESM.zip › Fig 4/Figure 4M/lncMtDloop_northern blot_m.tif]

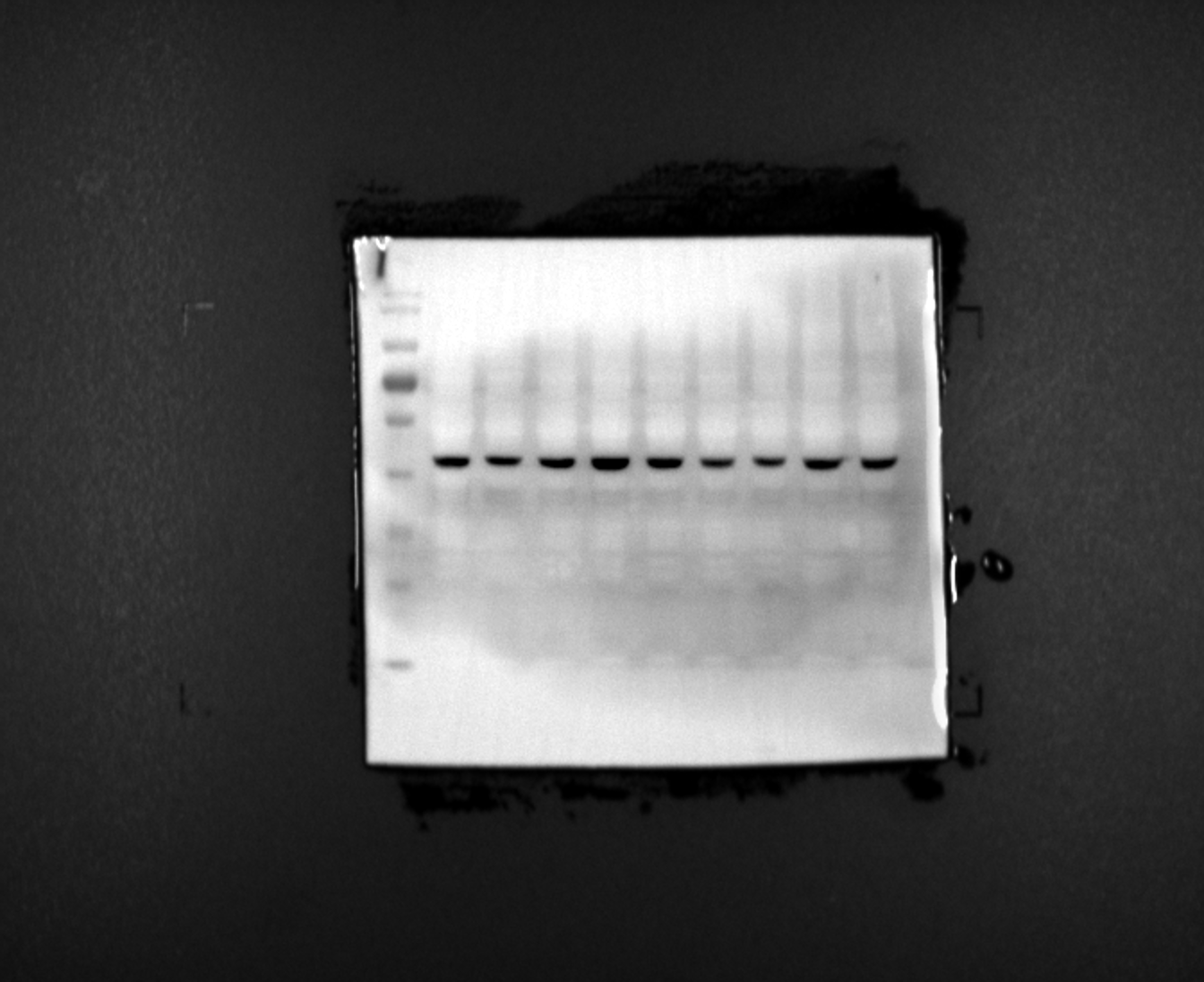

Supplement: Supplementary file 10 — Source data Fig. 5 [file 44318_2024_270_MOESM10_ESM.zip › Fig 5/5G/Western blot-actin.Tif]

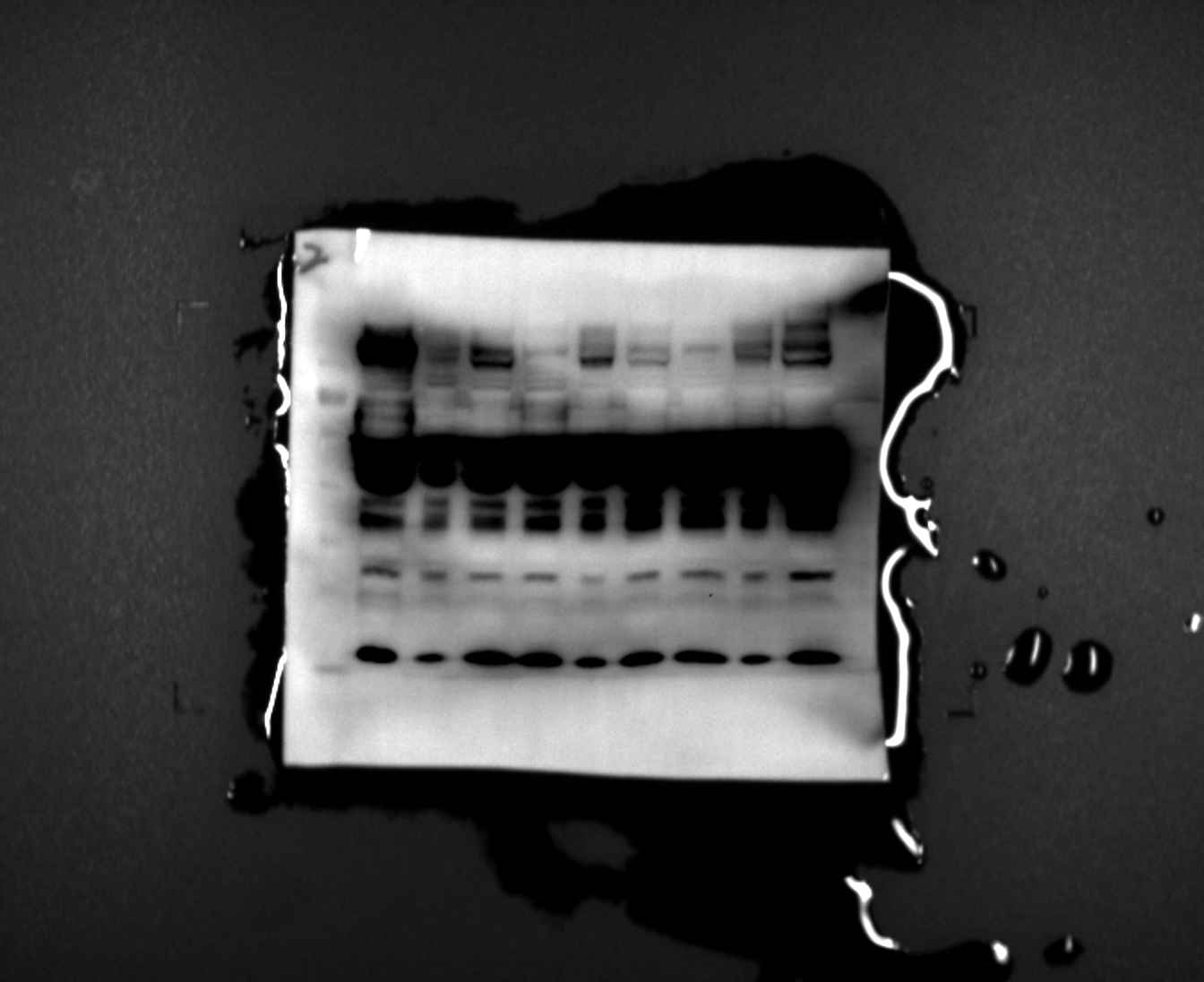

Supplement: Supplementary file 10 — Source data Fig. 5 [file 44318_2024_270_MOESM10_ESM.zip › Fig 5/5G/Western blot-complex IV, II,I.Tif]

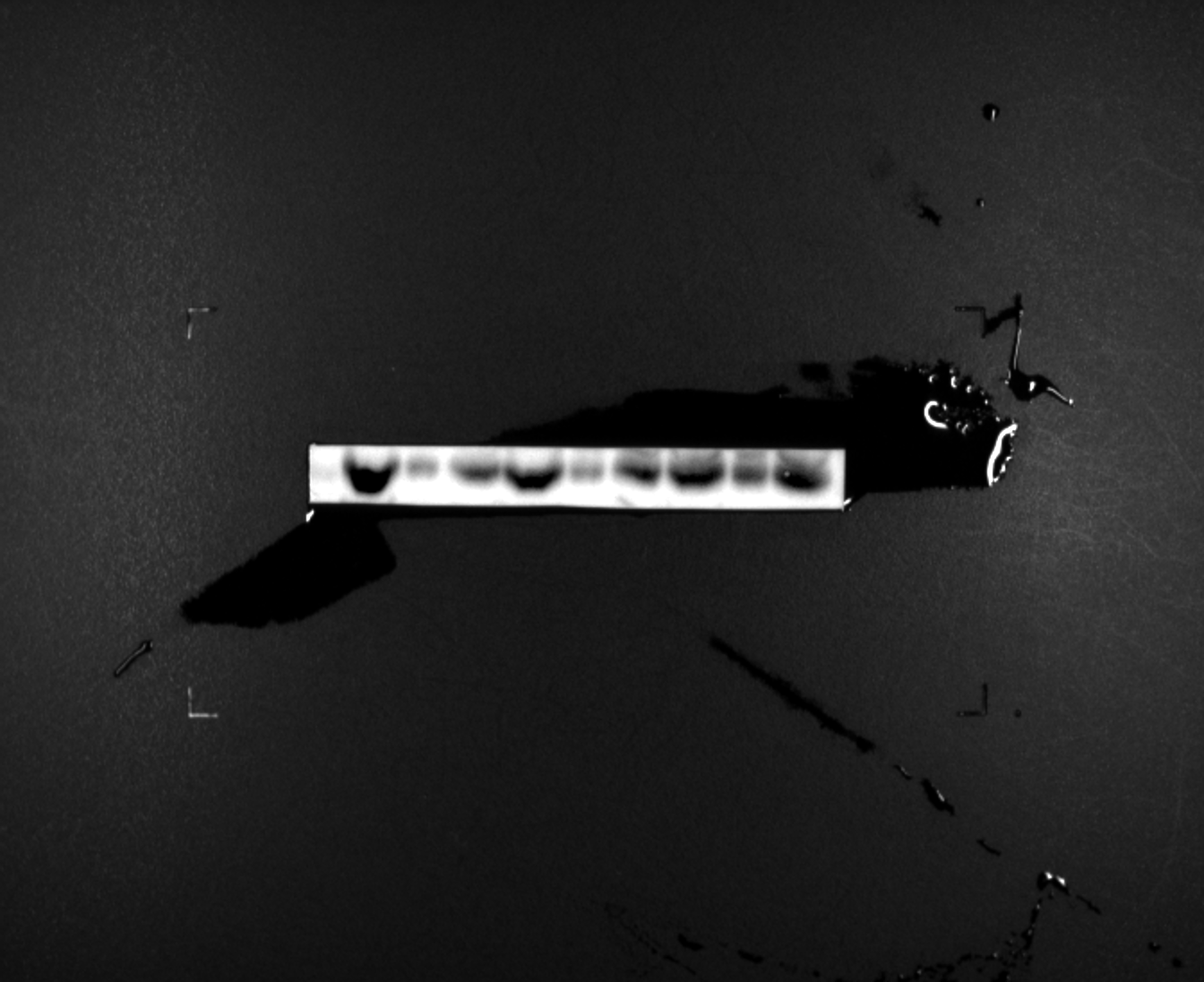

Supplement: Supplementary file 10 — Source data Fig. 5 [file 44318_2024_270_MOESM10_ESM.zip › Fig 5/5G/Western blot-complex IV.Tif]

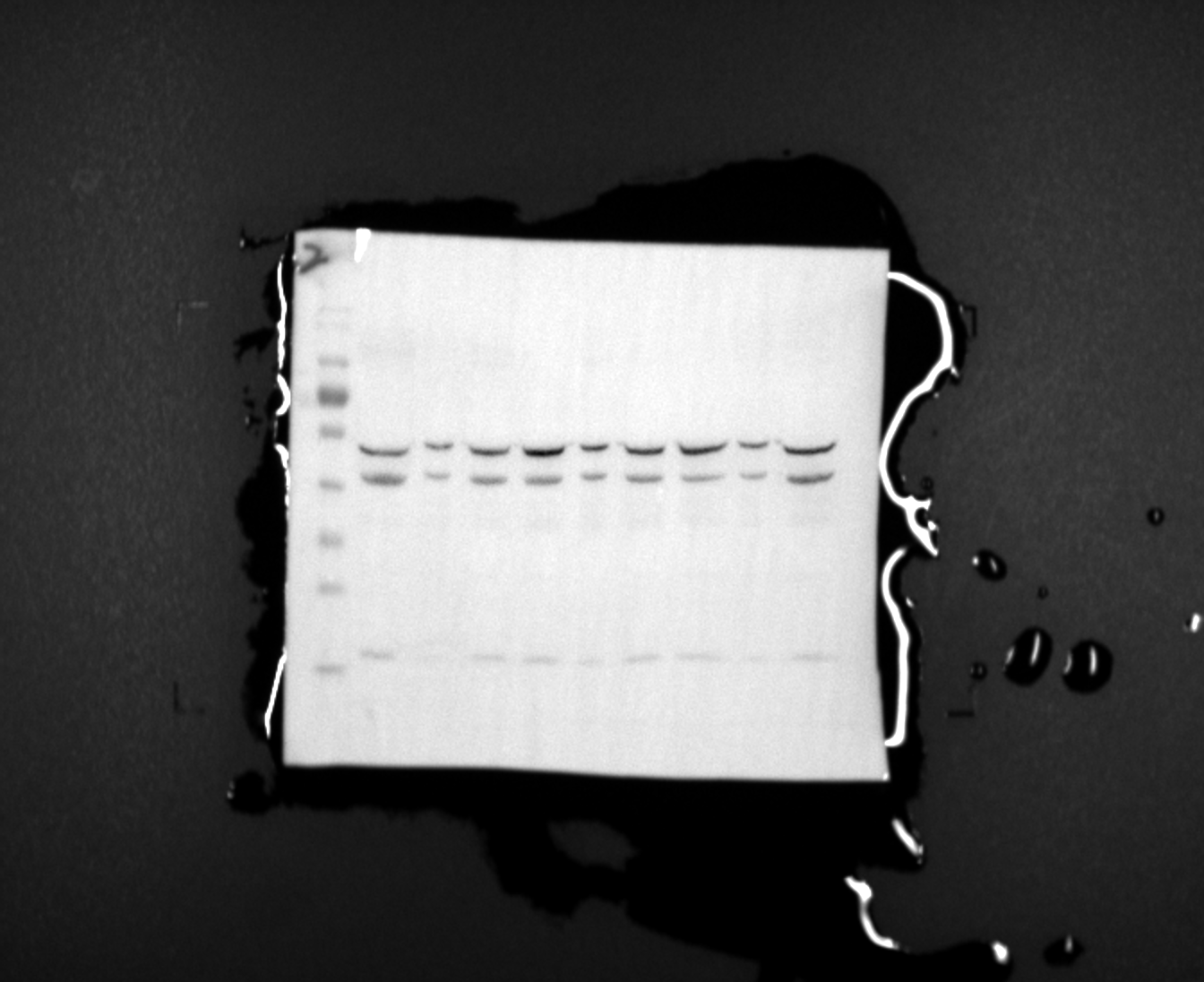

Supplement: Supplementary file 10 — Source data Fig. 5 [file 44318_2024_270_MOESM10_ESM.zip › Fig 5/5G/Western blot-complex V,III.Tif]

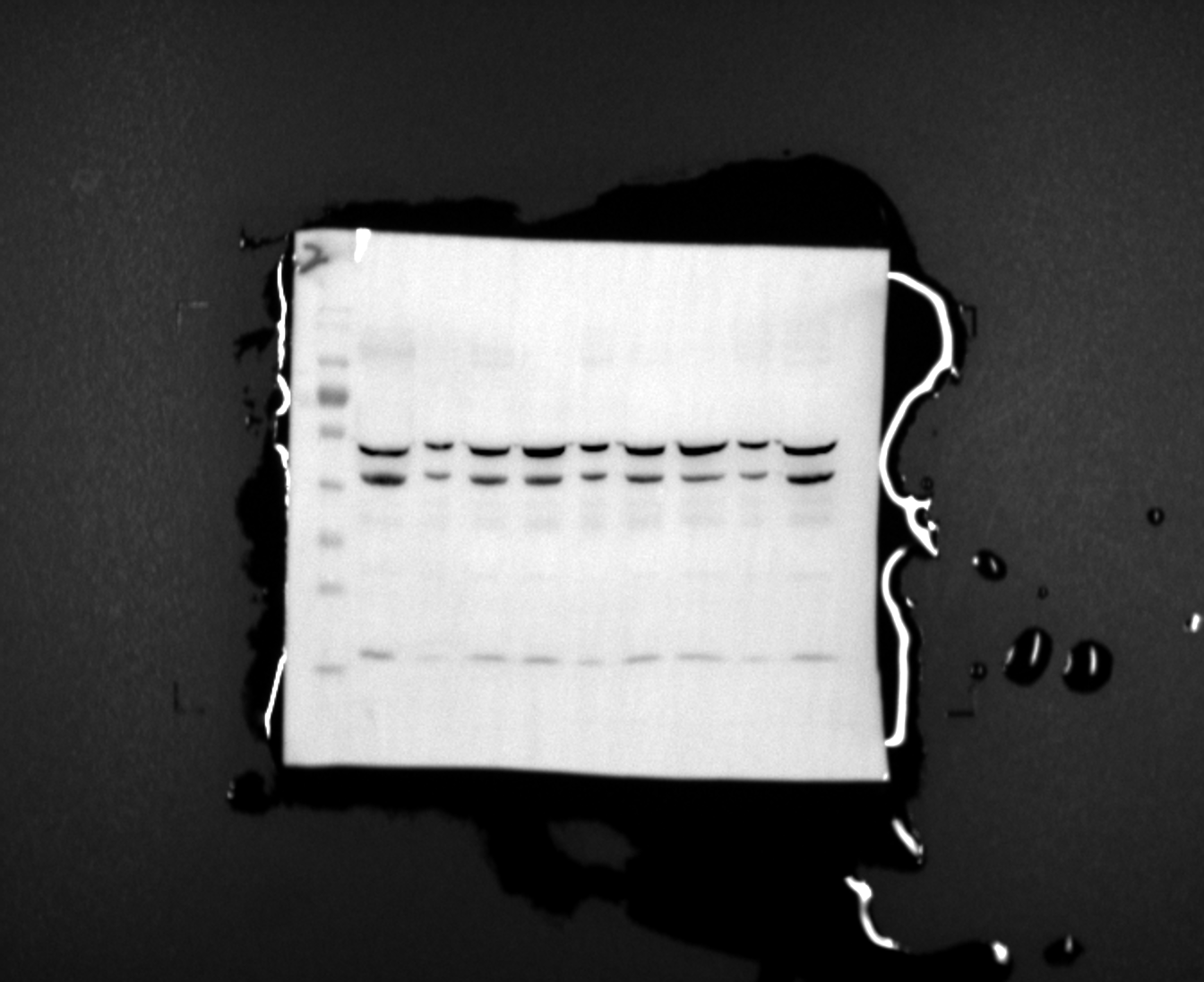

Supplement: Supplementary file 10 — Source data Fig. 5 [file 44318_2024_270_MOESM10_ESM.zip › Fig 5/5G/Western blot-complex.Tif]

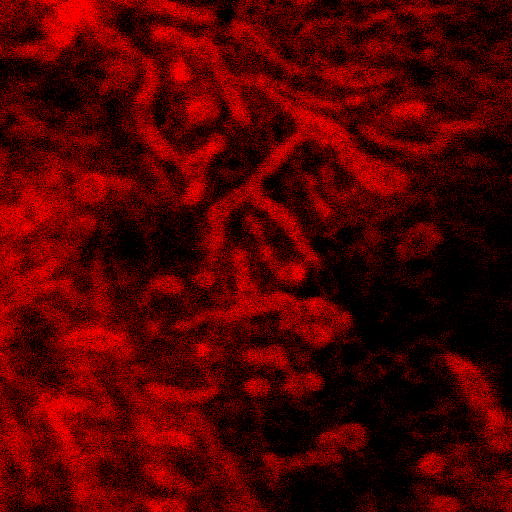

Supplement: Supplementary file 10 — Source data Fig. 5 [file 44318_2024_270_MOESM10_ESM.zip › Fig 5/5I/STED-3xTg-LV-control.tif]

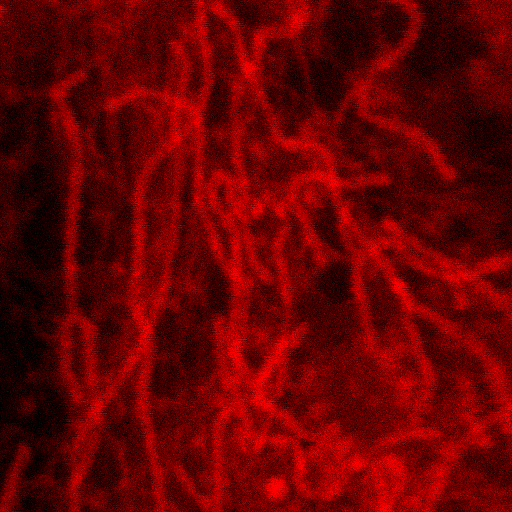

Supplement: Supplementary file 10 — Source data Fig. 5 [file 44318_2024_270_MOESM10_ESM.zip › Fig 5/5I/STED-3xTg-LV-lncMtDloop.tif]

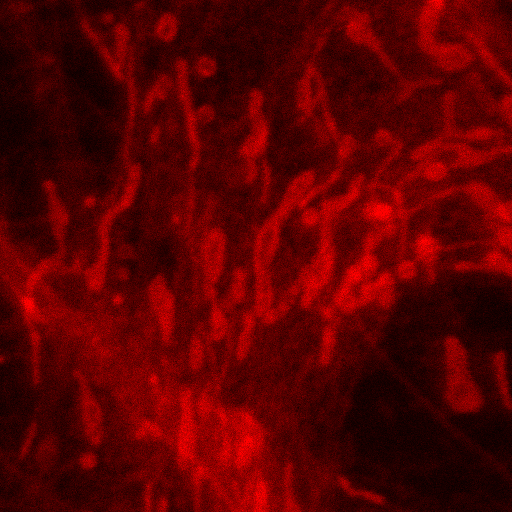

Supplement: Supplementary file 10 — Source data Fig. 5 [file 44318_2024_270_MOESM10_ESM.zip › Fig 5/5I/STED-3xTg-NC.tif]

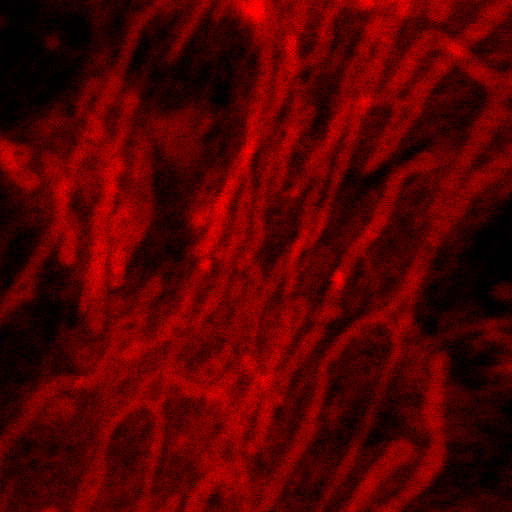

Supplement: Supplementary file 10 — Source data Fig. 5 [file 44318_2024_270_MOESM10_ESM.zip › Fig 5/5I/STED-WT-Lv-lncMtDloop.tif]

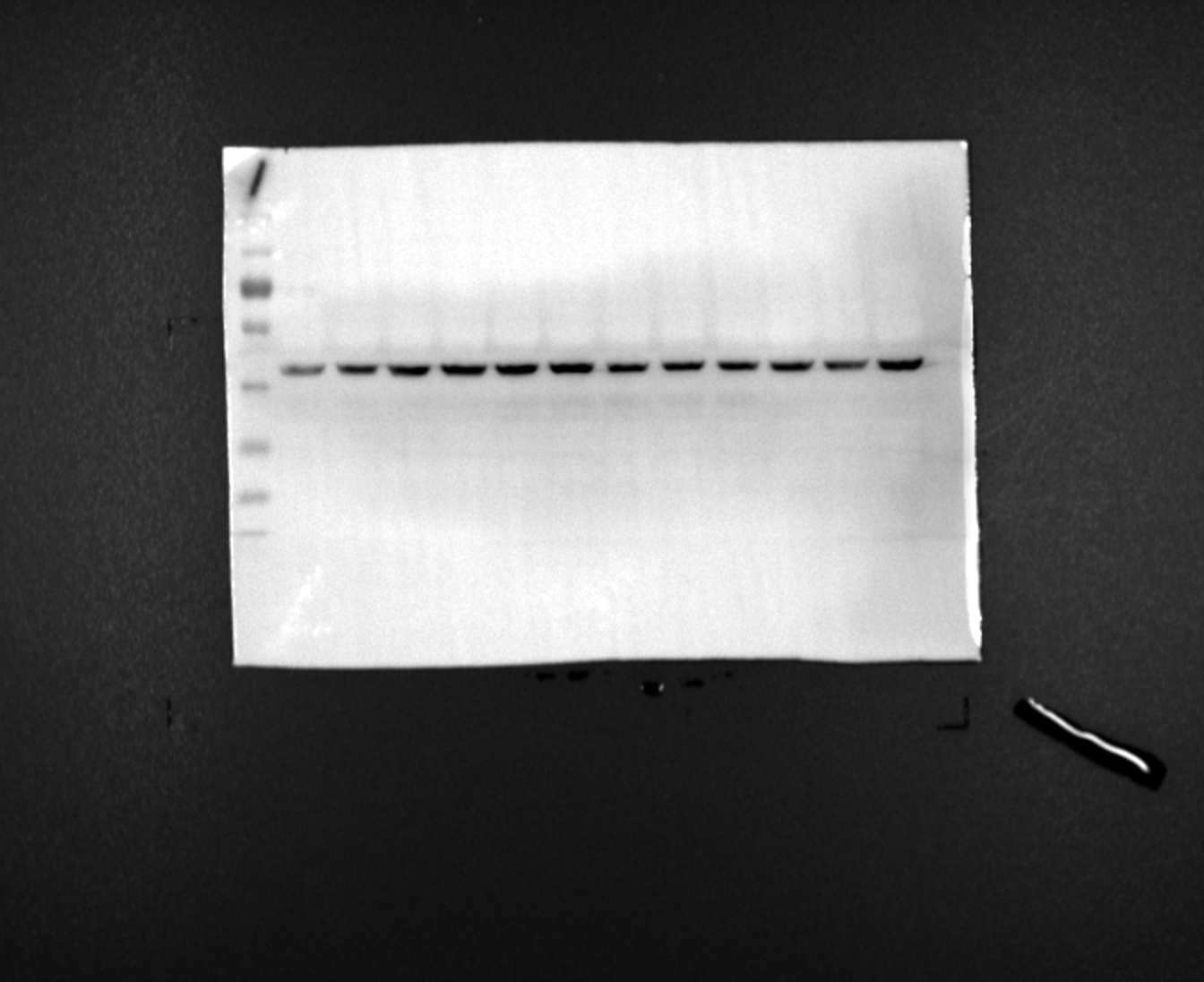

Supplement: Supplementary file 10 — Source data Fig. 5 [file 44318_2024_270_MOESM10_ESM.zip › Fig 5/5N/Western blot-actin.Tif]

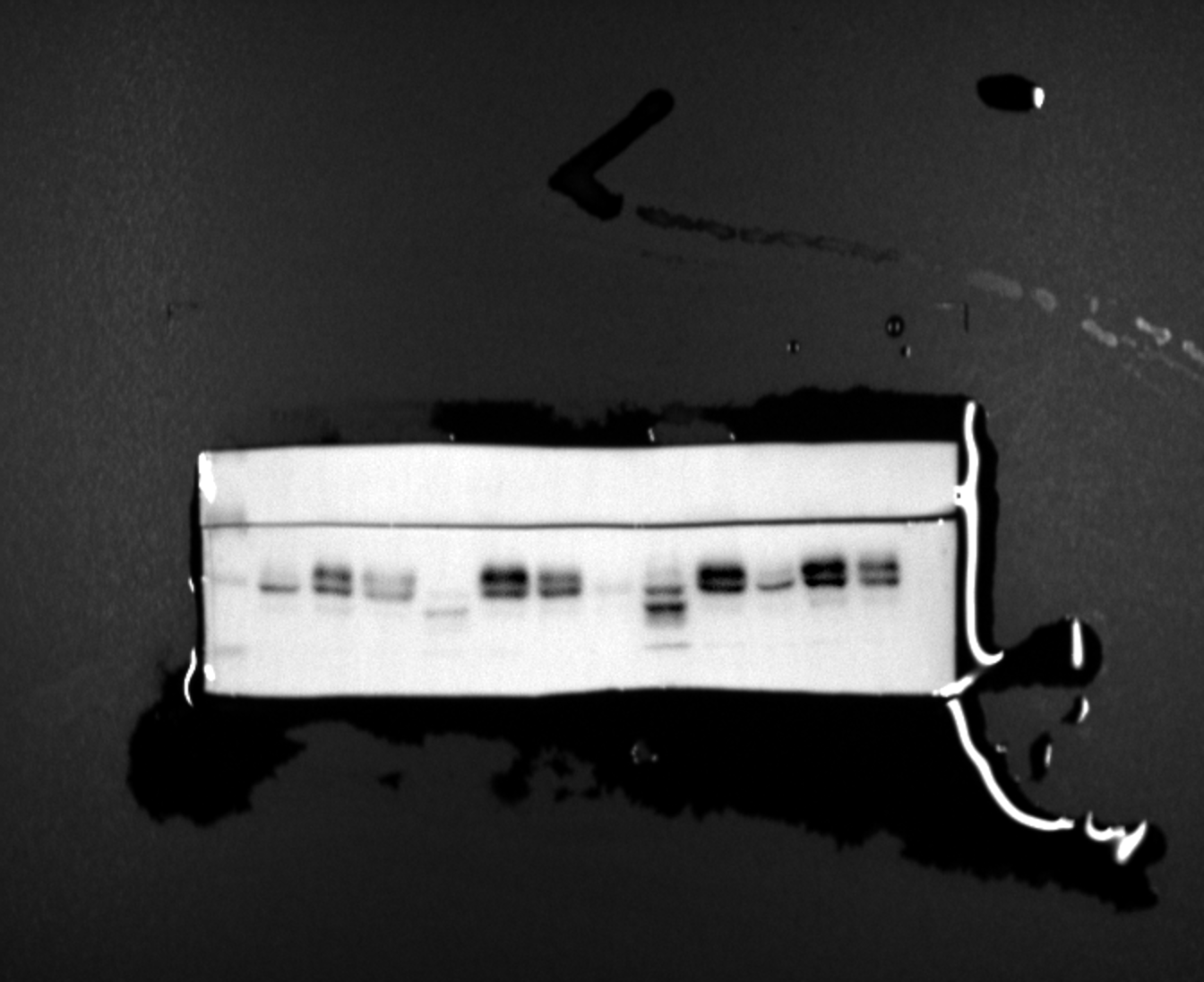

Supplement: Supplementary file 10 — Source data Fig. 5 [file 44318_2024_270_MOESM10_ESM.zip › Fig 5/5N/Western blot-Drp1.Tif]

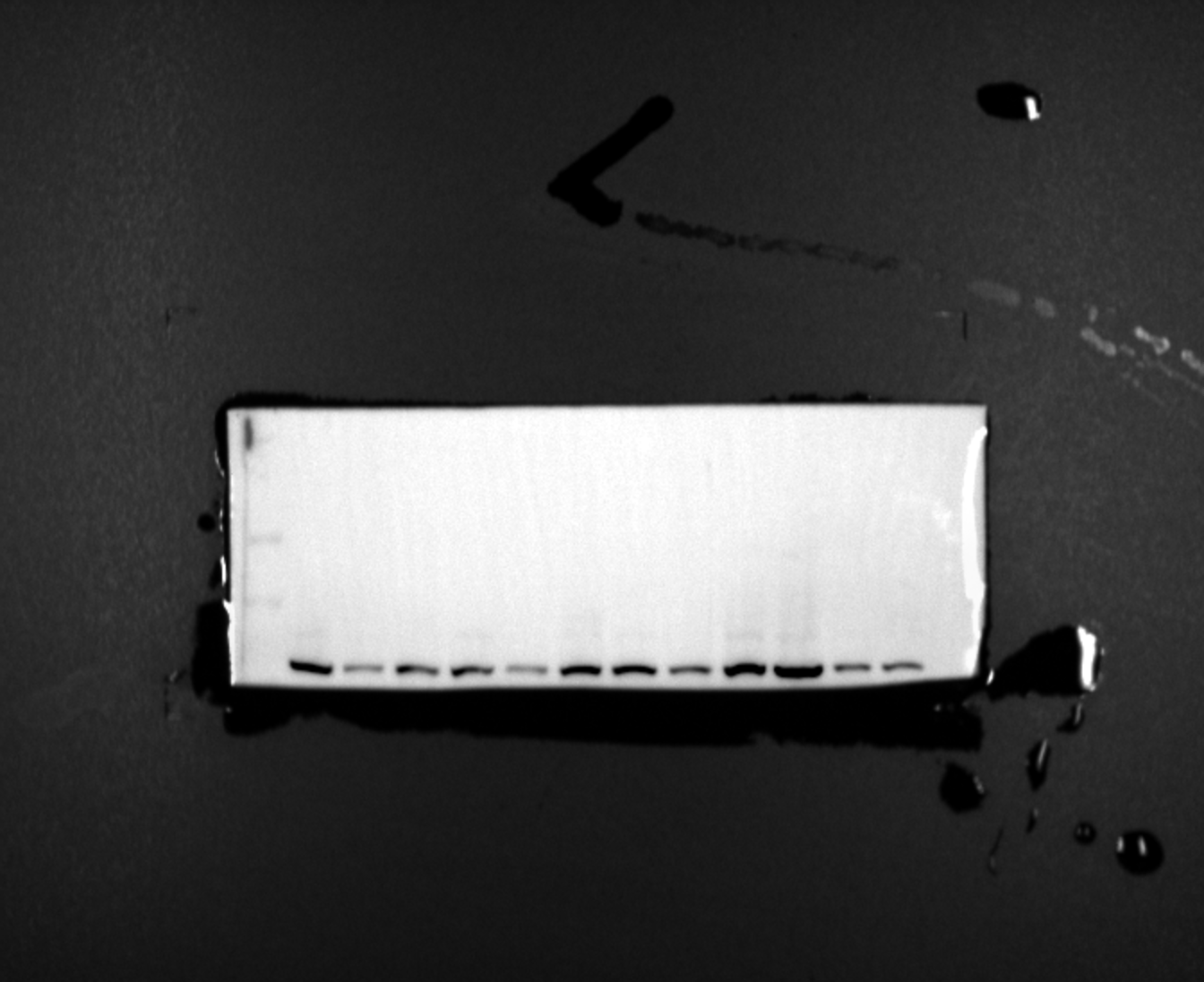

Supplement: Supplementary file 10 — Source data Fig. 5 [file 44318_2024_270_MOESM10_ESM.zip › Fig 5/5N/Western blot-Opa1.Tif]

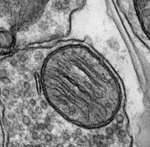

Supplement: Supplementary file 11 — Source data Fig. 6 [file 44318_2024_270_MOESM11_ESM.zip › Fig 6/6B/Class I.tif]

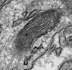

Supplement: Supplementary file 11 — Source data Fig. 6 [file 44318_2024_270_MOESM11_ESM.zip › Fig 6/6B/Class II.tif]

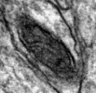

Supplement: Supplementary file 11 — Source data Fig. 6 [file 44318_2024_270_MOESM11_ESM.zip › Fig 6/6B/Class III.tif]

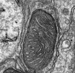

Supplement: Supplementary file 11 — Source data Fig. 6 [file 44318_2024_270_MOESM11_ESM.zip › Fig 6/6C/Class A.tif]

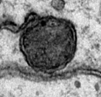

Supplement: Supplementary file 11 — Source data Fig. 6 [file 44318_2024_270_MOESM11_ESM.zip › Fig 6/6C/Class B.tif]

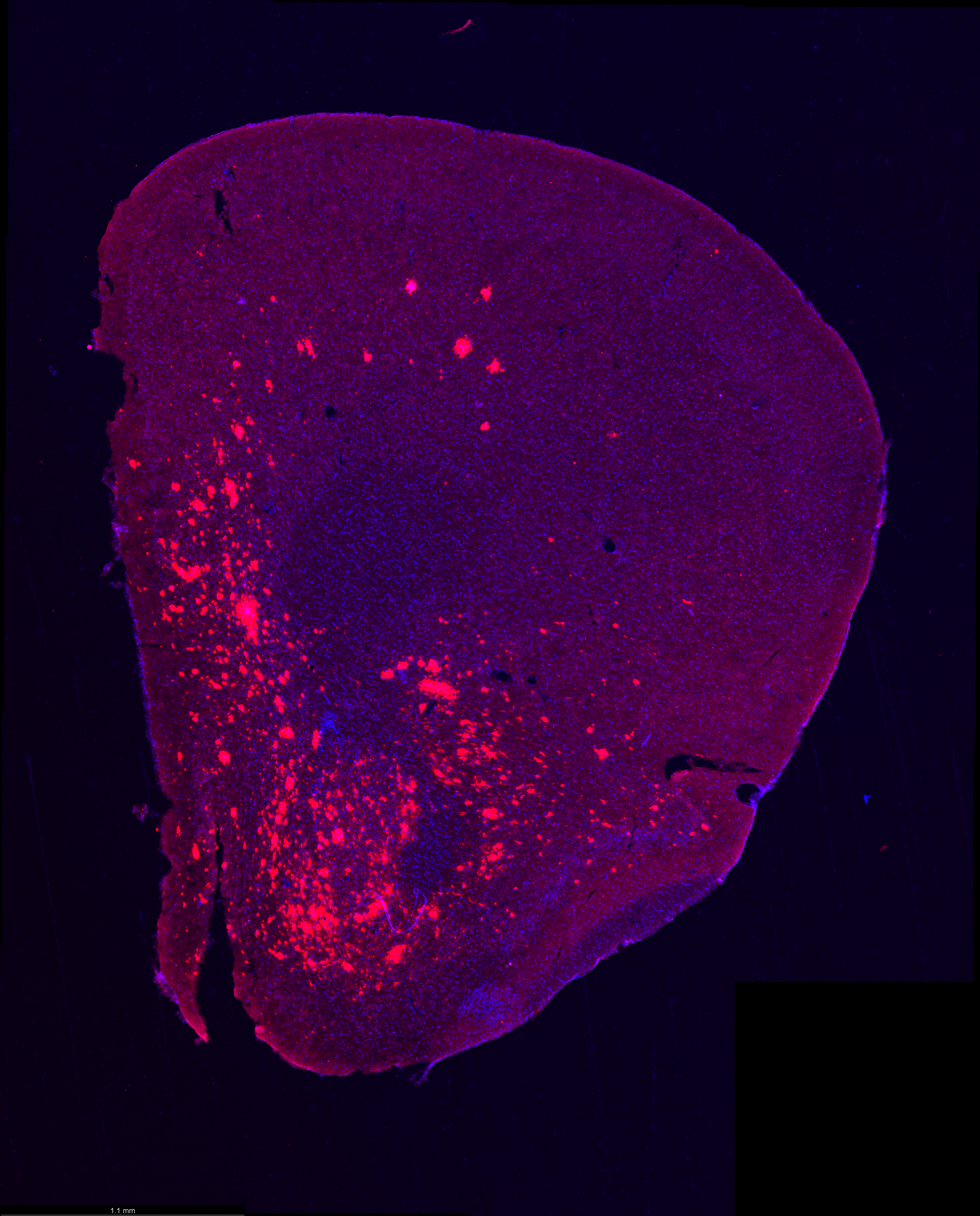

Supplement: Supplementary file 12 — Source data Fig. 7 [file 44318_2024_270_MOESM12_ESM.zip › Fig 7/7B/IF-6E10-3xTg-PFC-AAV-control.tif]

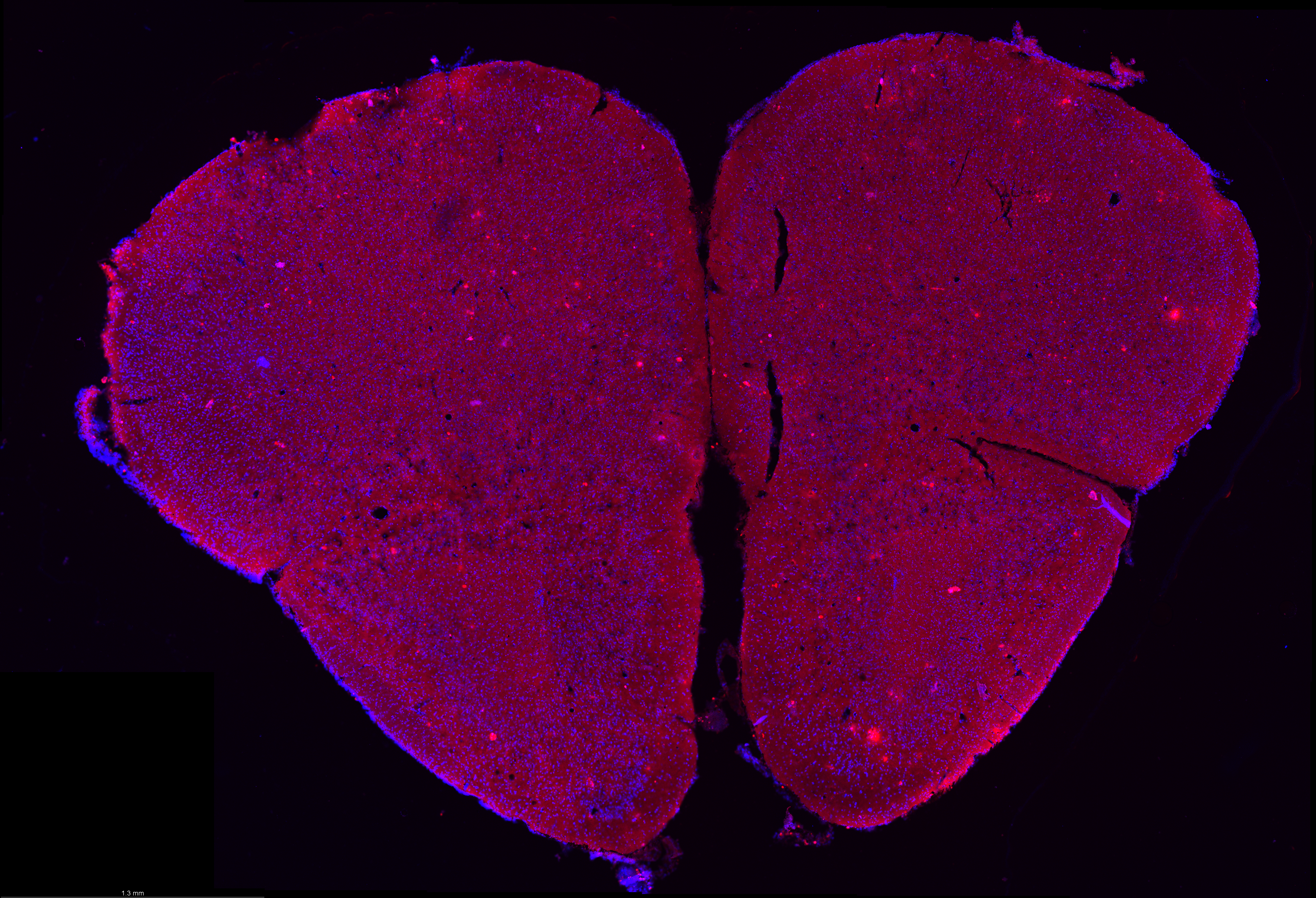

Supplement: Supplementary file 12 — Source data Fig. 7 [file 44318_2024_270_MOESM12_ESM.zip › Fig 7/7B/IF-6E10-WT-PFC-AAV-control.tif]

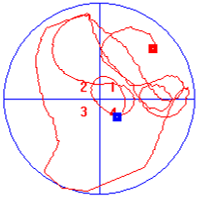

Supplement: Supplementary file 12 — Source data Fig. 7 [file 44318_2024_270_MOESM12_ESM.zip › Fig 7/7N/3xTg-AAV-control.png]

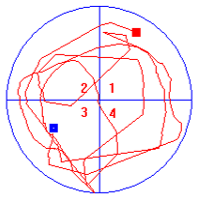

Supplement: Supplementary file 12 — Source data Fig. 7 [file 44318_2024_270_MOESM12_ESM.zip › Fig 7/7N/3xTg-AAV-lncMtDloop.png]

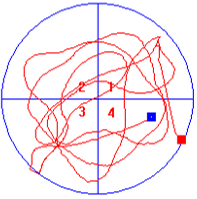

Supplement: Supplementary file 12 — Source data Fig. 7 [file 44318_2024_270_MOESM12_ESM.zip › Fig 7/7N/WT-AAV-control.png]

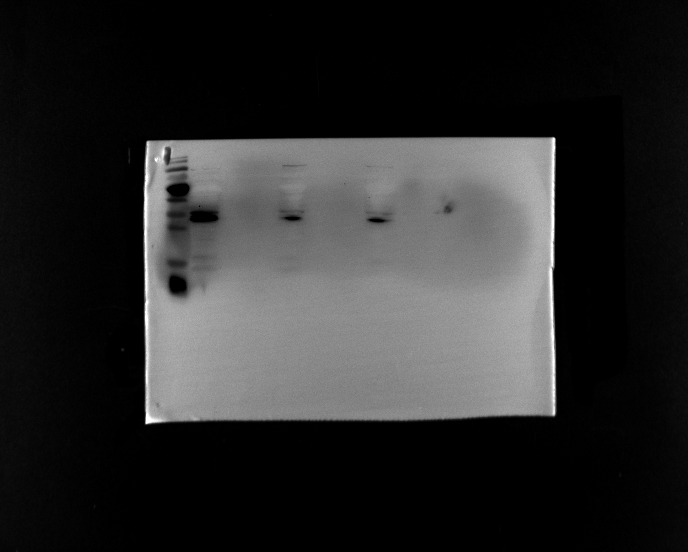

Supplement: Supplementary file 14 — Source Data Fig. EV2 [file 44318_2024_270_MOESM14_ESM.zip › EV2/Figue EV2N/RNA pull-down merge.tif]

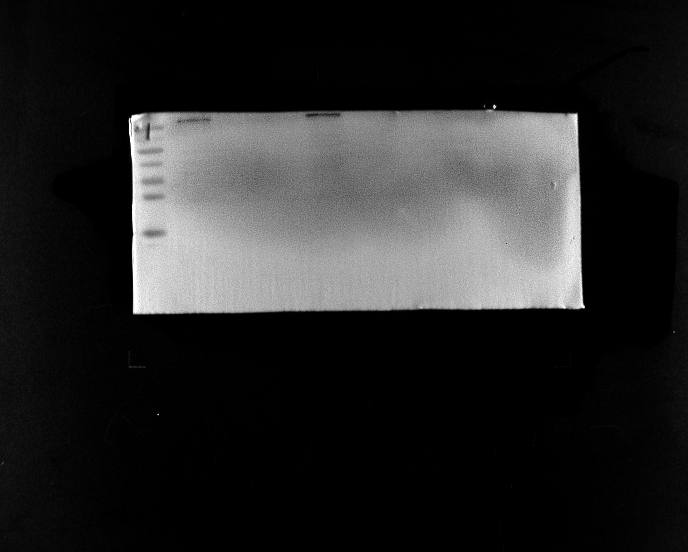

Supplement: Supplementary file 14 — Source Data Fig. EV2 [file 44318_2024_270_MOESM14_ESM.zip › EV2/Figure EV2C/RNA pull-down-actin-merge.tif]

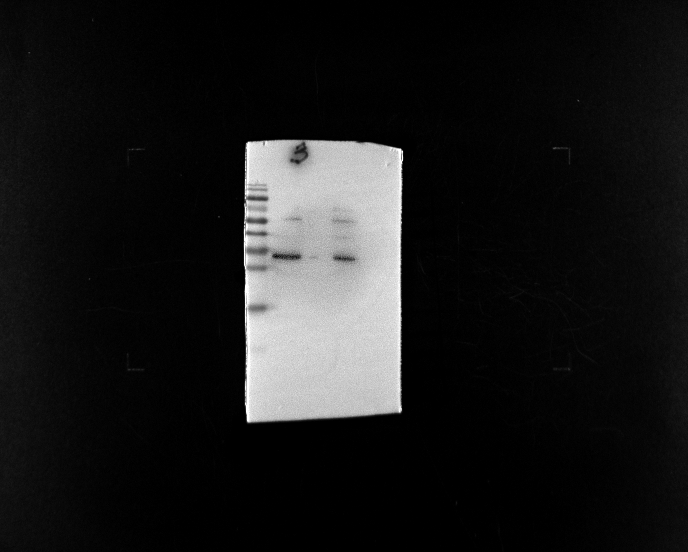

Supplement: Supplementary file 14 — Source Data Fig. EV2 [file 44318_2024_270_MOESM14_ESM.zip › EV2/Figure EV2C/RNA pull-down-alyref-merge.tif]

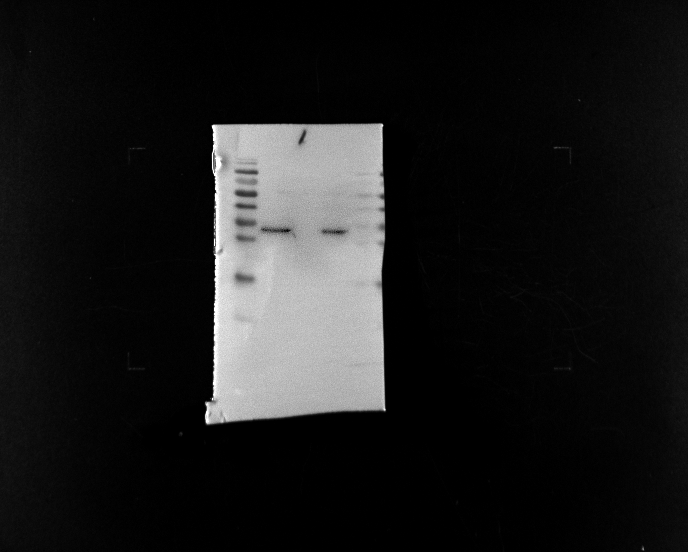

Supplement: Supplementary file 14 — Source Data Fig. EV2 [file 44318_2024_270_MOESM14_ESM.zip › EV2/Figure EV2C/RNA pull-down-p32-merge.tif]

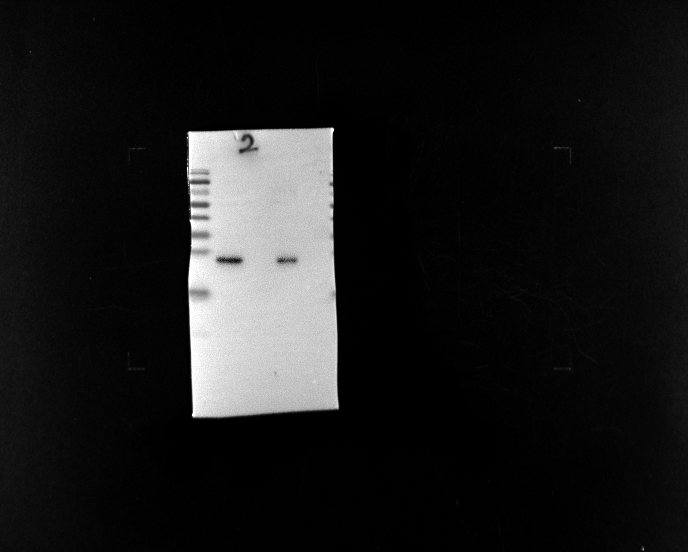

Supplement: Supplementary file 14 — Source Data Fig. EV2 [file 44318_2024_270_MOESM14_ESM.zip › EV2/Figure EV2C/RNA pull-down-tppp-merge.tif]

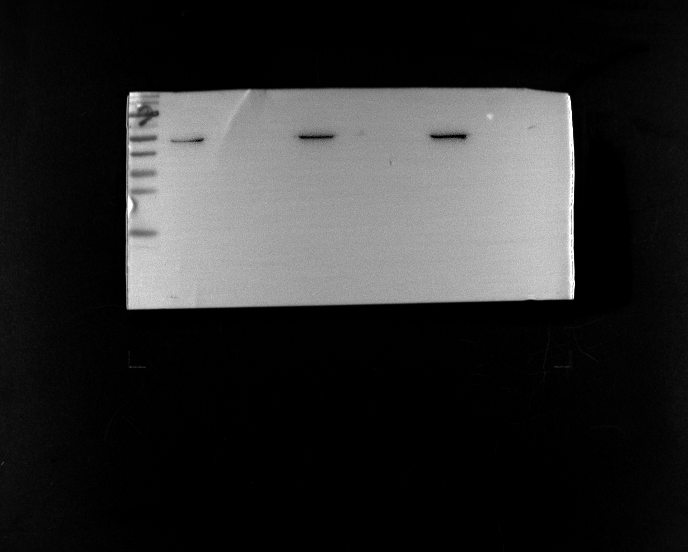

Supplement: Supplementary file 14 — Source Data Fig. EV2 [file 44318_2024_270_MOESM14_ESM.zip › EV2/Figure EV2D/RNA pull-down-ATP5a-merge.tif]

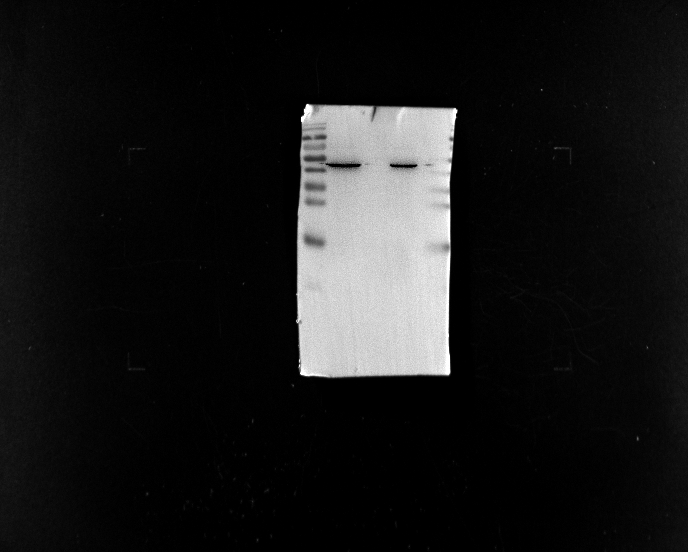

Supplement: Supplementary file 14 — Source Data Fig. EV2 [file 44318_2024_270_MOESM14_ESM.zip › EV2/Figure EV2D/RNA pull-down-ATP5d-merge.tif]

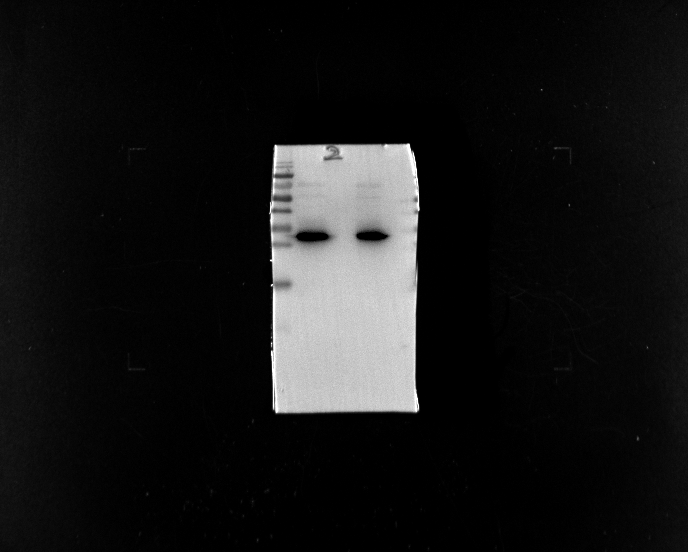

Supplement: Supplementary file 14 — Source Data Fig. EV2 [file 44318_2024_270_MOESM14_ESM.zip › EV2/Figure EV2D/RNA pull-down-p32-merge.tif]

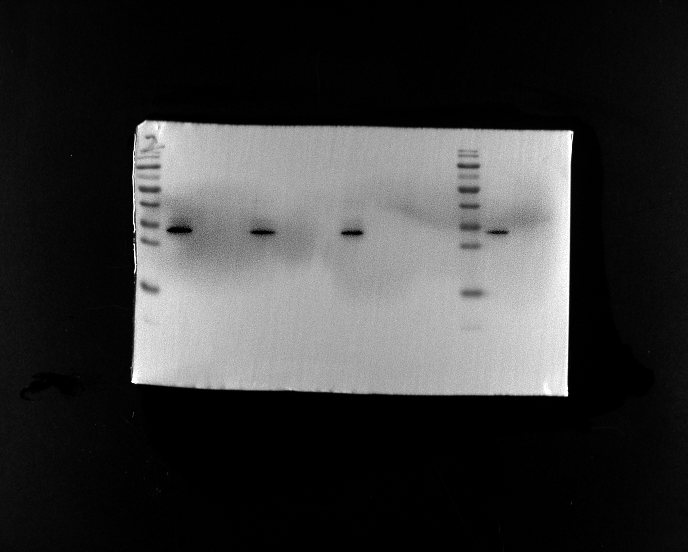

Supplement: Supplementary file 14 — Source Data Fig. EV2 [file 44318_2024_270_MOESM14_ESM.zip › EV2/Figure EV2D/RNA pull-down-Stx1b-merge.tif]

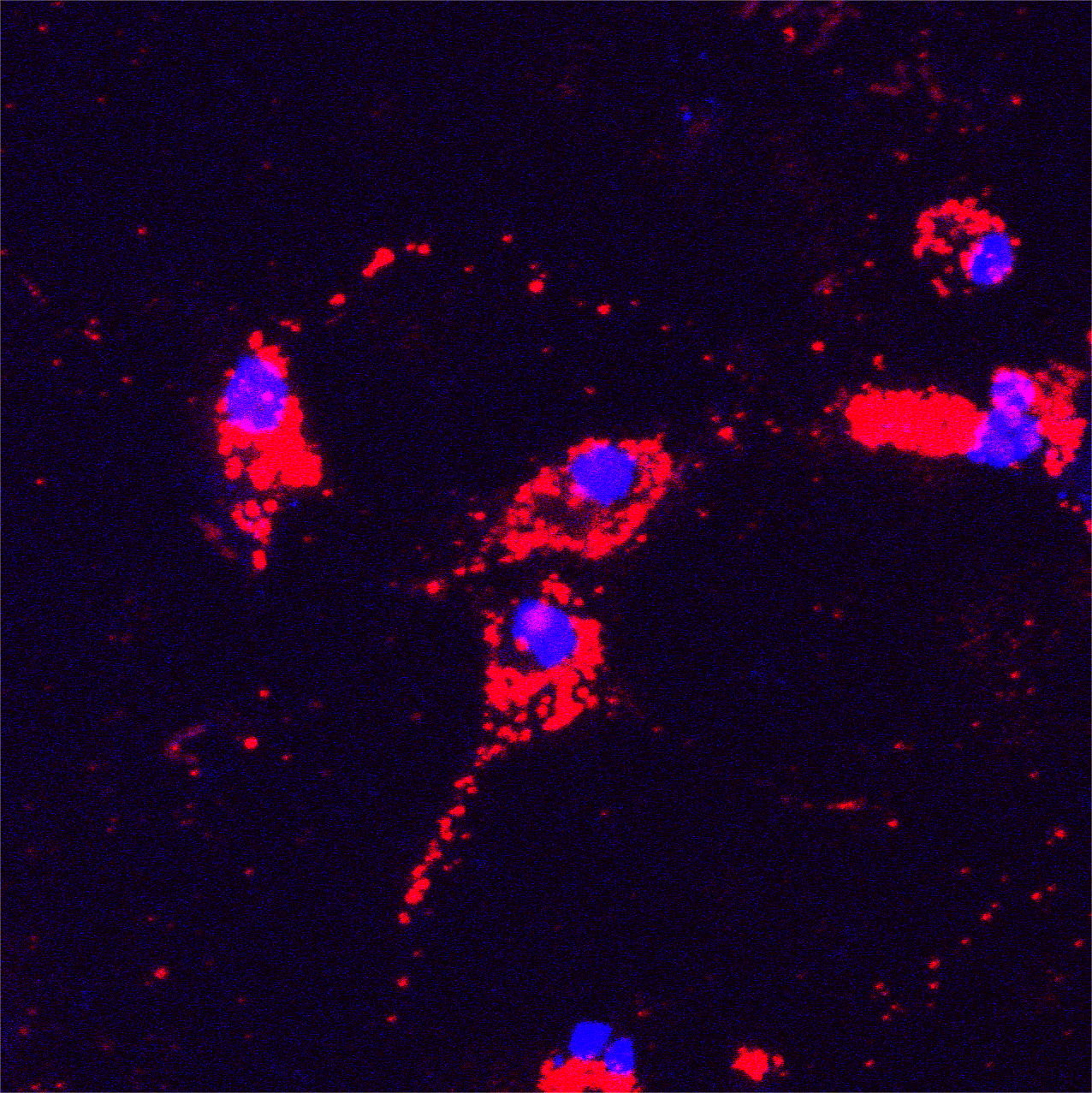

Supplement: Supplementary file 14 — Source Data Fig. EV2 [file 44318_2024_270_MOESM14_ESM.zip › EV2/Figure EV2E/IF-p32.png]

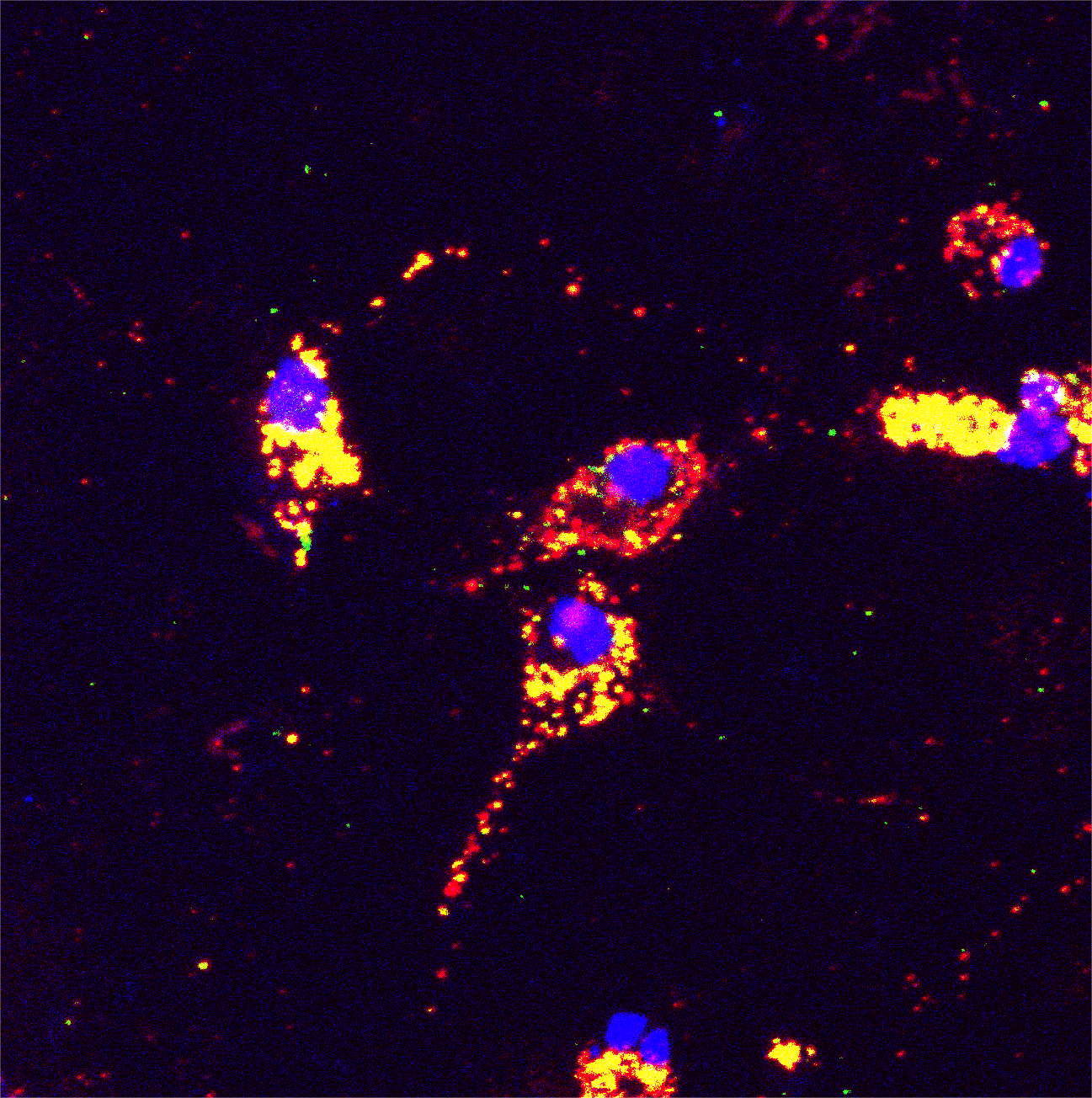

Supplement: Supplementary file 14 — Source Data Fig. EV2 [file 44318_2024_270_MOESM14_ESM.zip › EV2/Figure EV2E/Merge.png]

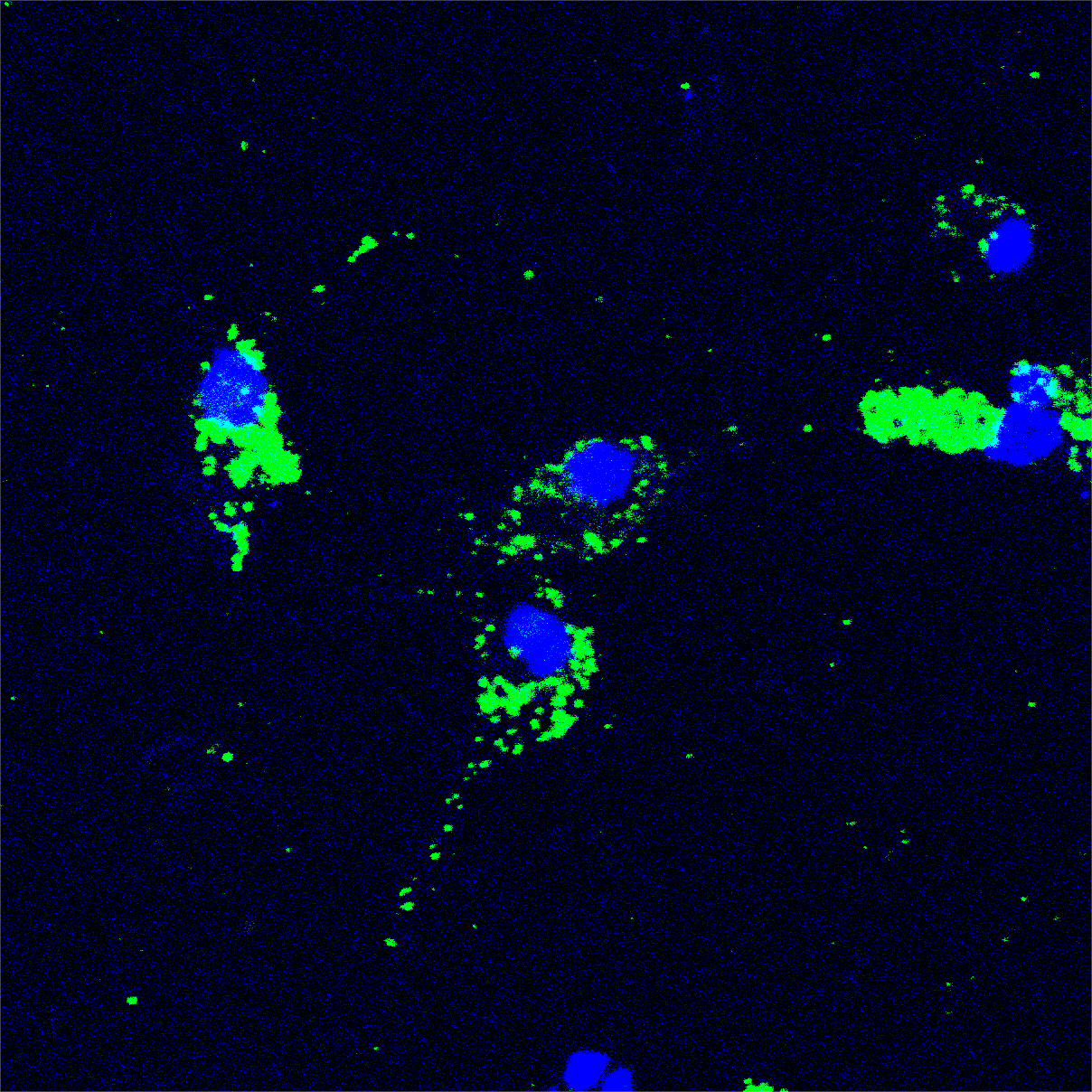

Supplement: Supplementary file 14 — Source Data Fig. EV2 [file 44318_2024_270_MOESM14_ESM.zip › EV2/Figure EV2E/RNAscope-lncMtDloop.png]

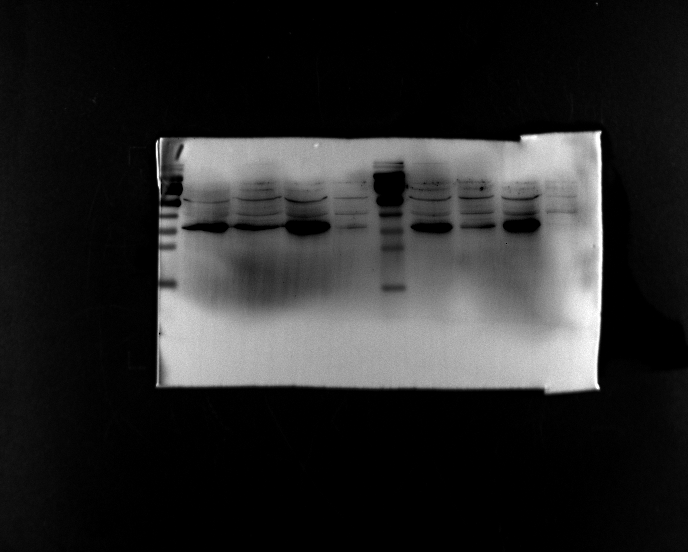

Supplement: Supplementary file 14 — Source Data Fig. EV2 [file 44318_2024_270_MOESM14_ESM.zip › EV2/Figure EV2H/siRNA-p32.tif]

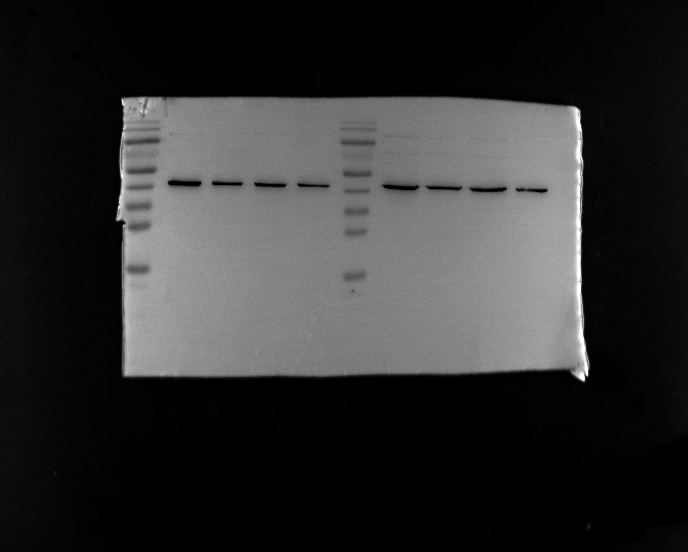

Supplement: Supplementary file 14 — Source Data Fig. EV2 [file 44318_2024_270_MOESM14_ESM.zip › EV2/Figure EV2H/a┬-actin.tif]

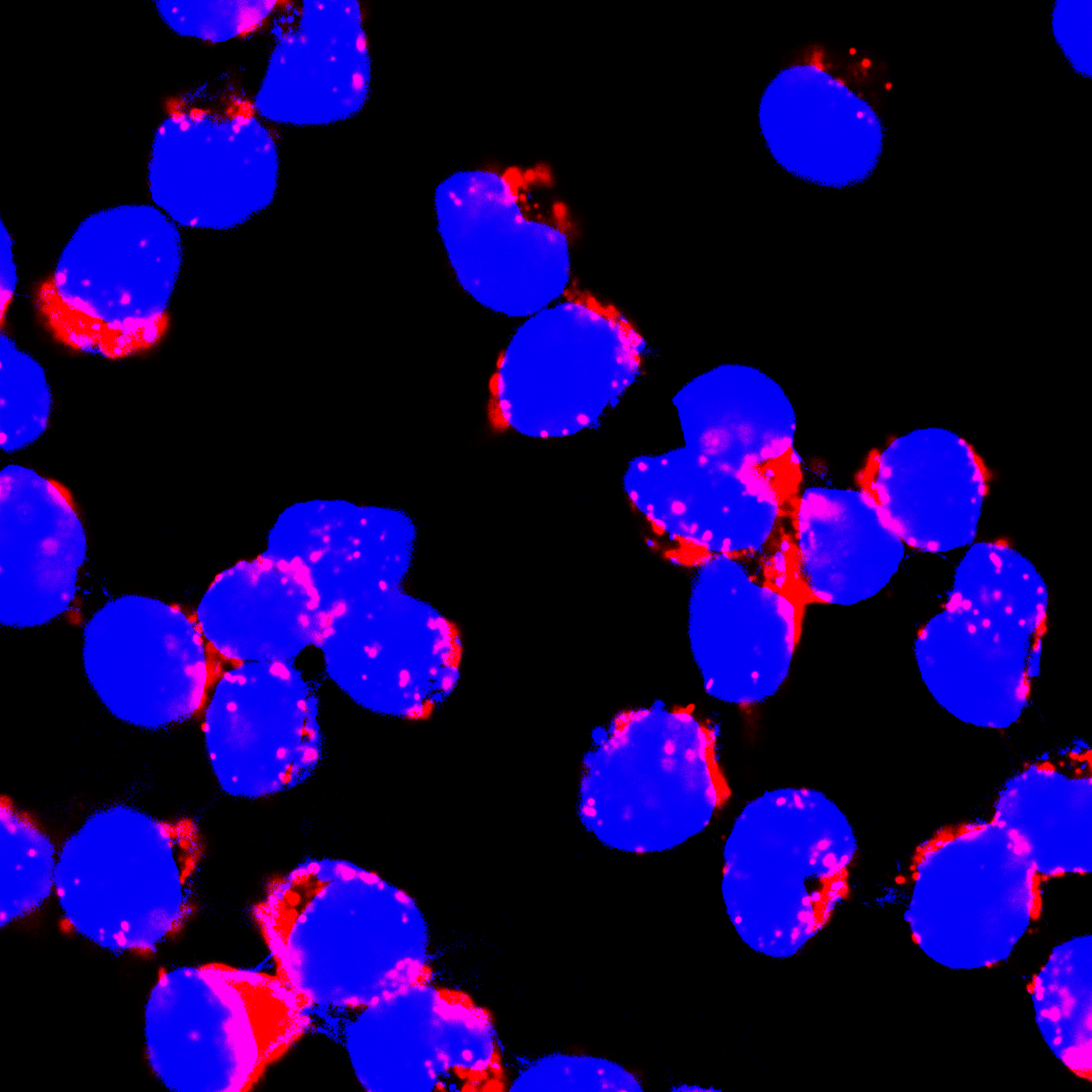

Supplement: Supplementary file 14 — Source Data Fig. EV2 [file 44318_2024_270_MOESM14_ESM.zip › EV2/Figure EV2L/siRNA-control-LV-lncMtDloop-lncMtDloop.tif]

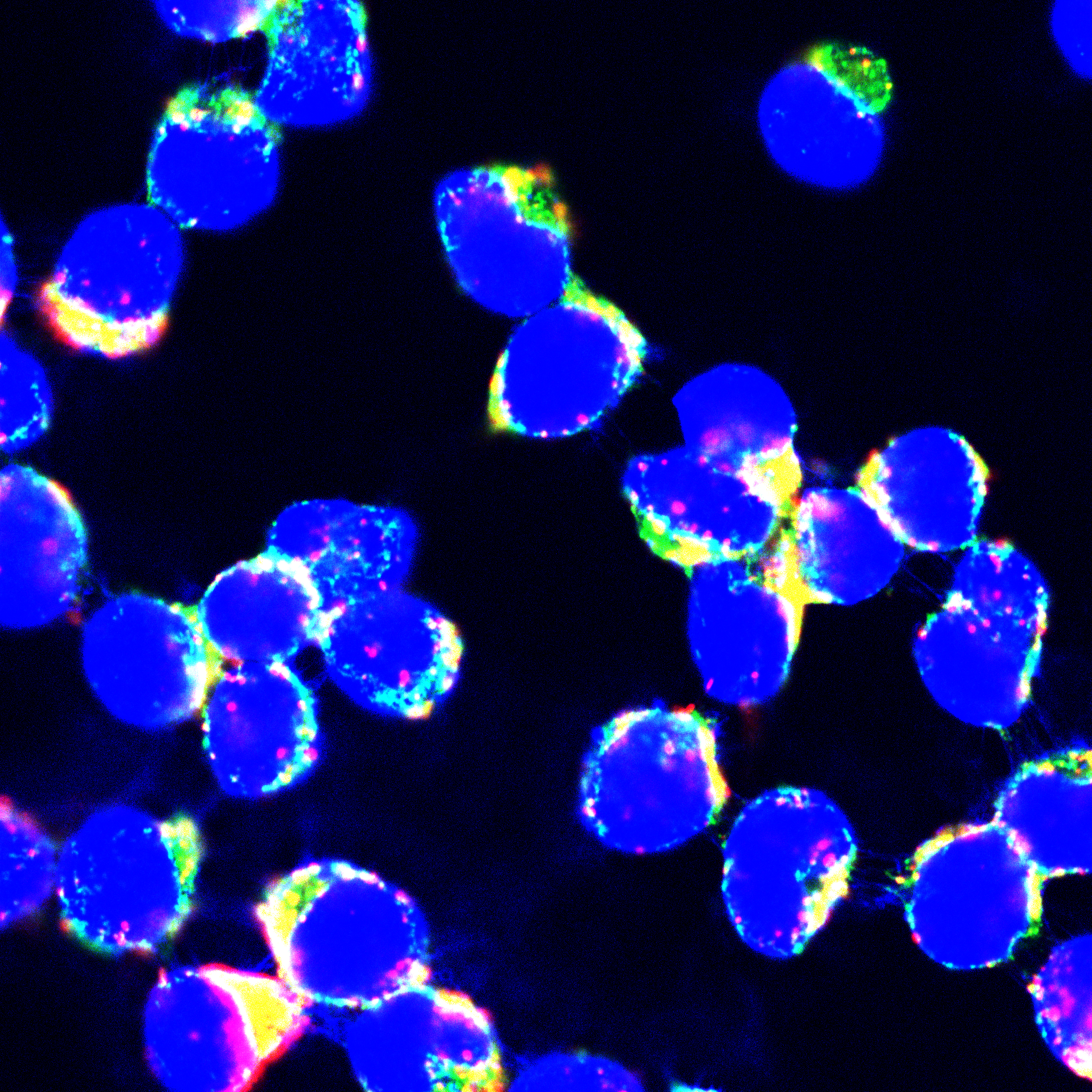

Supplement: Supplementary file 14 — Source Data Fig. EV2 [file 44318_2024_270_MOESM14_ESM.zip › EV2/Figure EV2L/siRNA-control-LV-lncMtDloop-merge.tif]

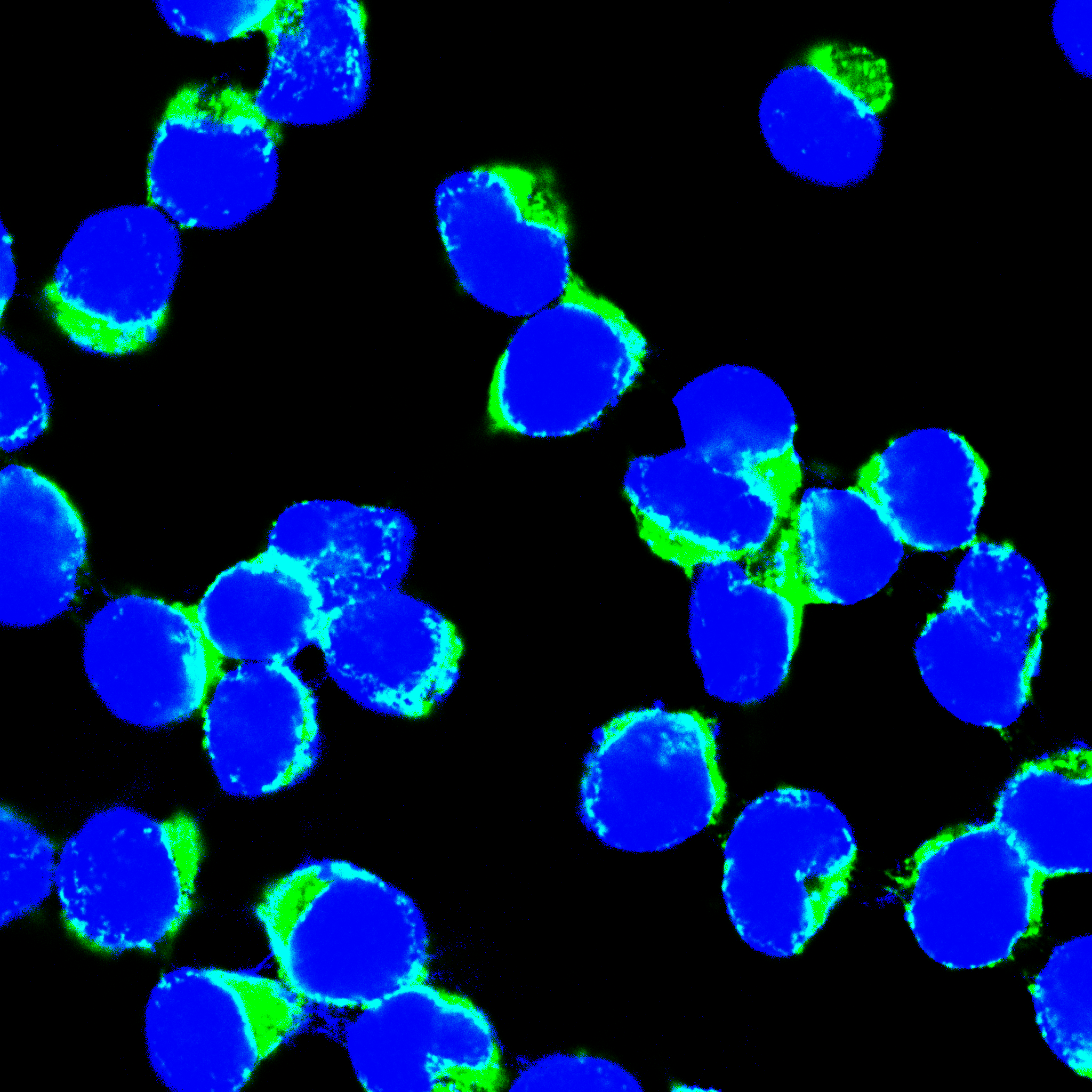

Supplement: Supplementary file 14 — Source Data Fig. EV2 [file 44318_2024_270_MOESM14_ESM.zip › EV2/Figure EV2L/siRNA-control-LV-lncMtDloop-TOMM20.tif]

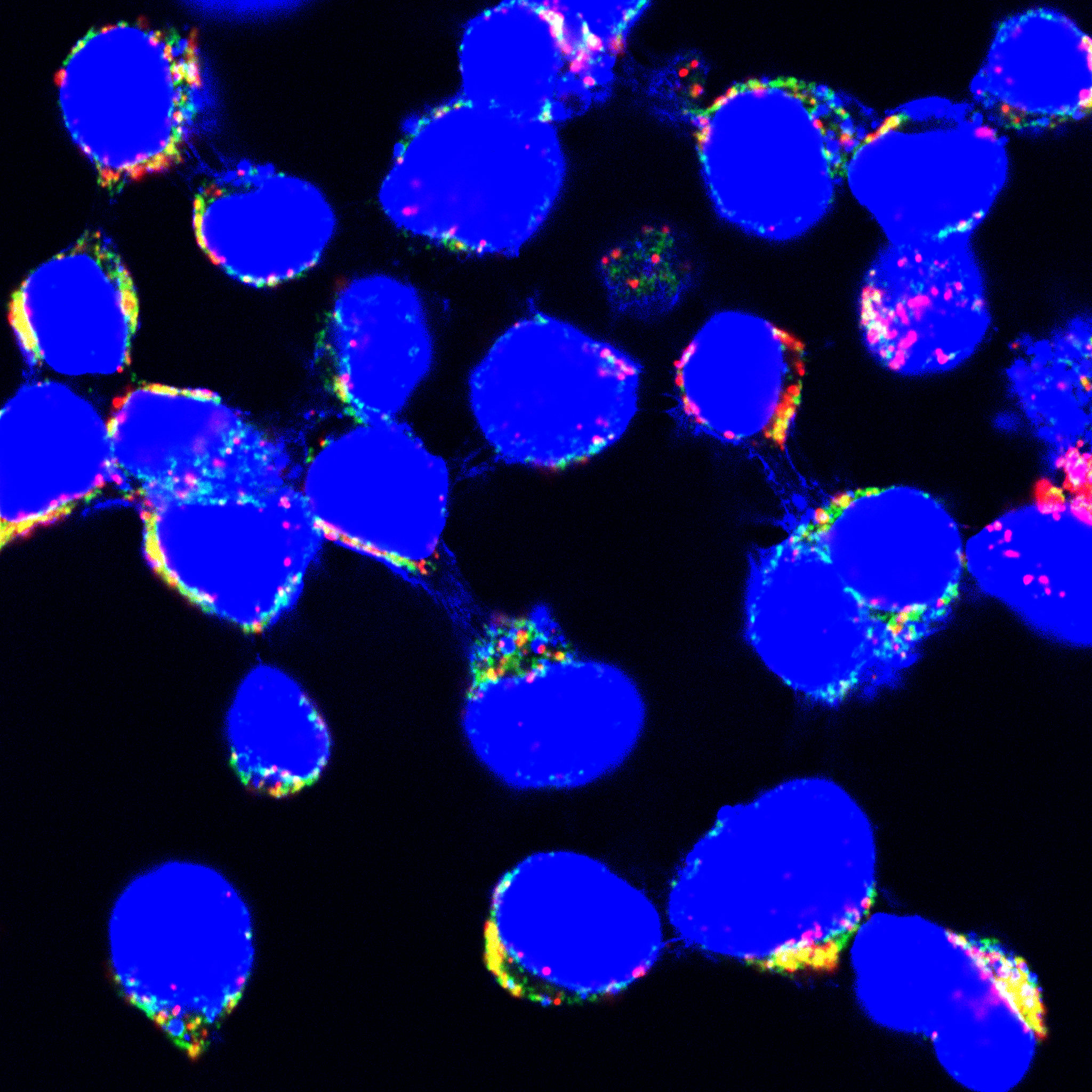

Supplement: Supplementary file 14 — Source Data Fig. EV2 [file 44318_2024_270_MOESM14_ESM.zip › EV2/Figure EV2L/siRNA-p32+LV-lncMtDloop-merge.tif]

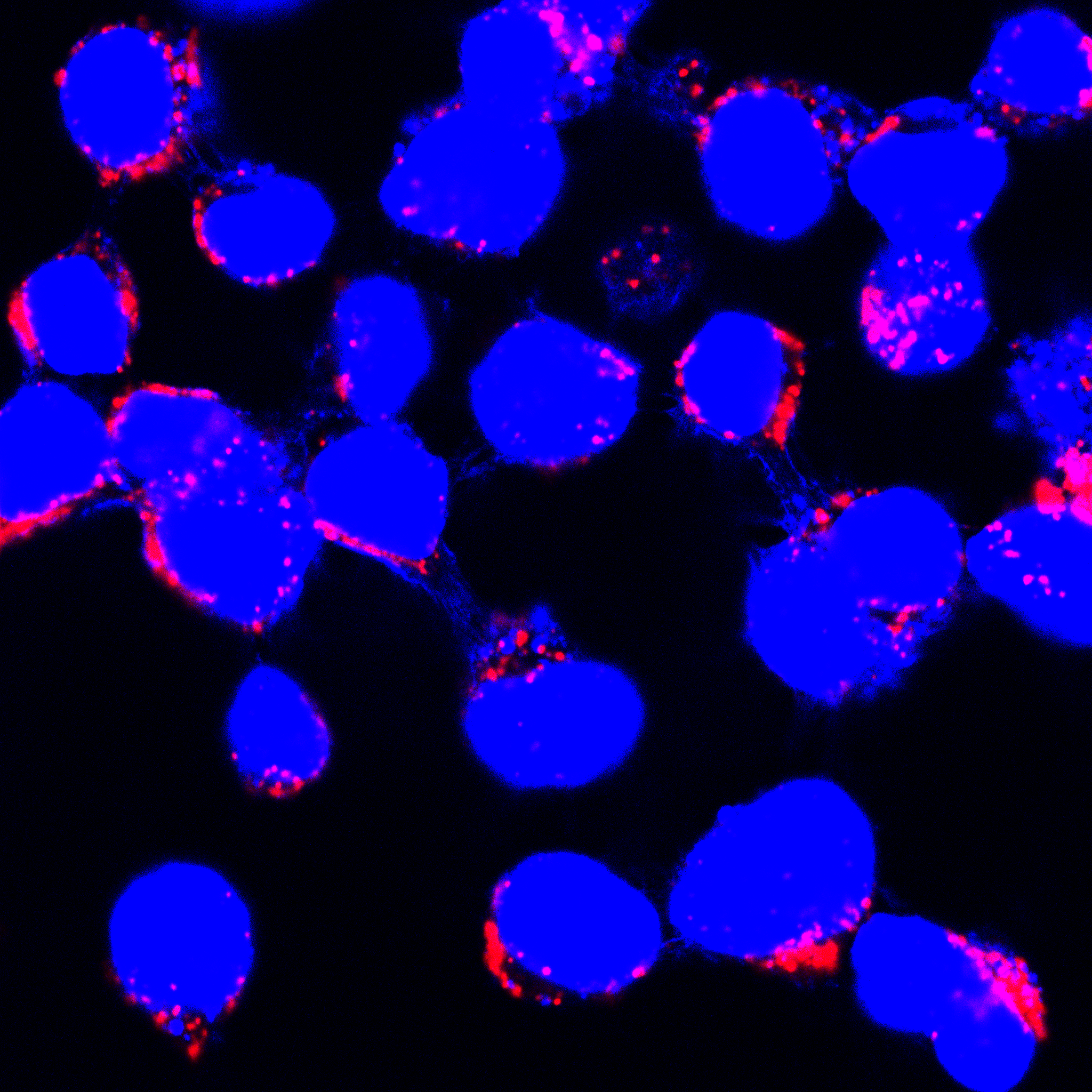

Supplement: Supplementary file 14 — Source Data Fig. EV2 [file 44318_2024_270_MOESM14_ESM.zip › EV2/Figure EV2L/siRNA-p32-LV-lncMtDloop-lncMtDloop.tif]

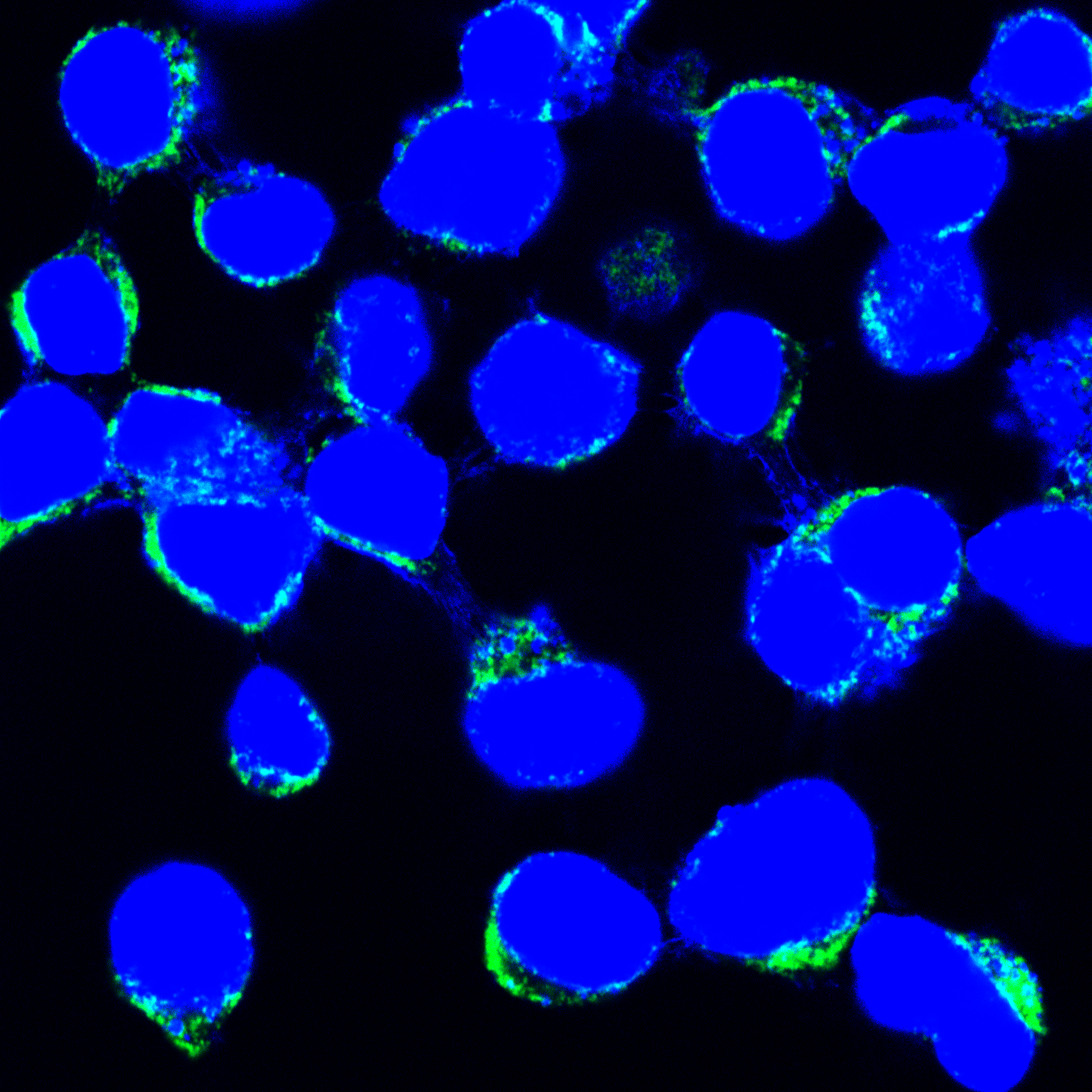

Supplement: Supplementary file 14 — Source Data Fig. EV2 [file 44318_2024_270_MOESM14_ESM.zip › EV2/Figure EV2L/siRNA-p32-LV-lncMtDloop-TOMM20.tif]

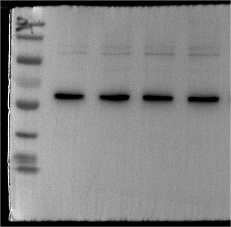

Supplement: Supplementary file 15 — Source Data Fig. EV3 [file 44318_2024_270_MOESM15_ESM.zip › EV3/Figure EV3A/PNPASE (within 4 lanes from left).png]

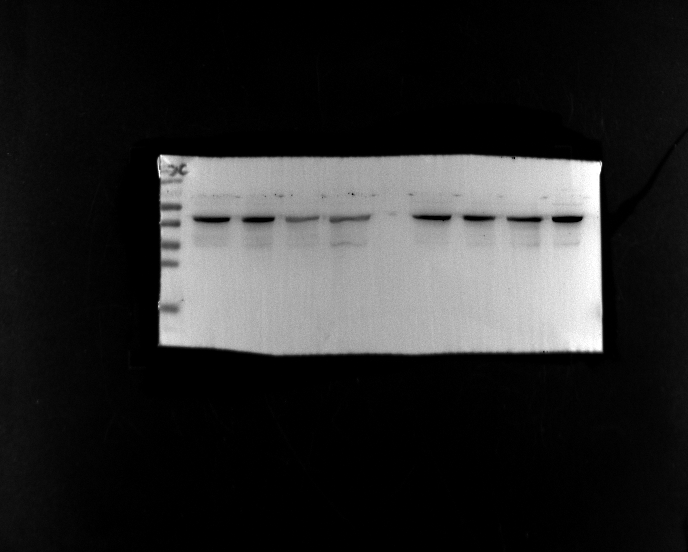

Supplement: Supplementary file 15 — Source Data Fig. EV3 [file 44318_2024_270_MOESM15_ESM.zip › EV3/Figure EV3A/a┬-actin (within 4 lanes from right).tif]

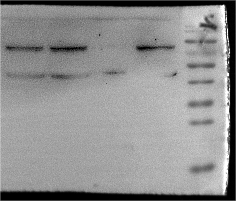

Supplement: Supplementary file 15 — Source Data Fig. EV3 [file 44318_2024_270_MOESM15_ESM.zip › EV3/Figure EV3B/PNPASE.png]

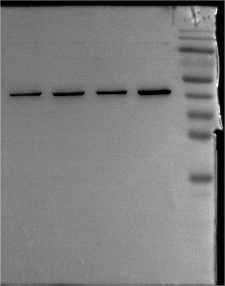

Supplement: Supplementary file 15 — Source Data Fig. EV3 [file 44318_2024_270_MOESM15_ESM.zip › EV3/Figure EV3B/a┬-actin.png]

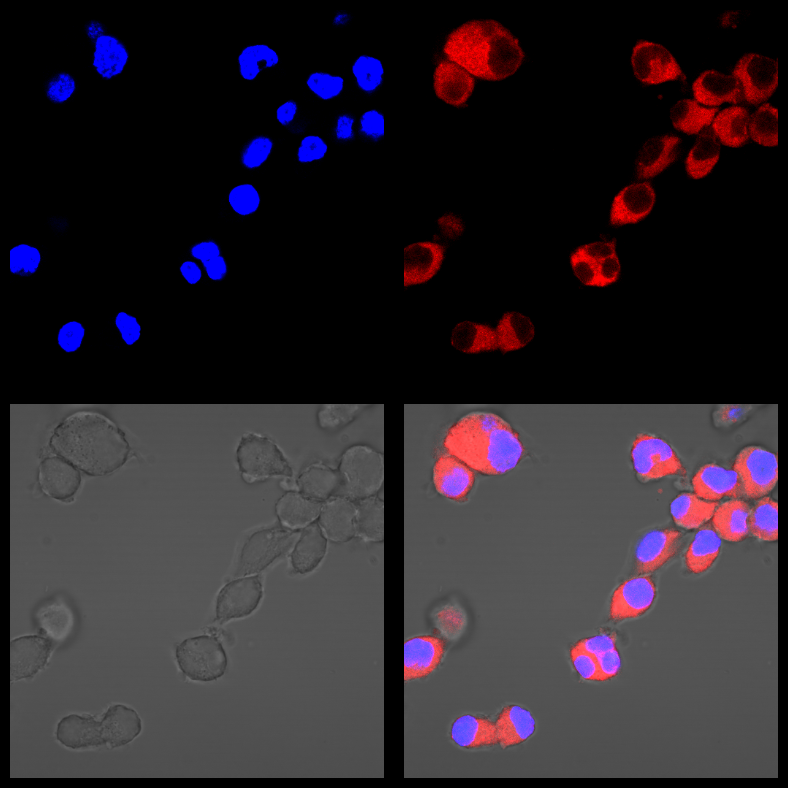

Supplement: Supplementary file 15 — Source Data Fig. EV3 [file 44318_2024_270_MOESM15_ESM.zip › EV3/Figure EV3C/LV-control +EB 0.bmp]

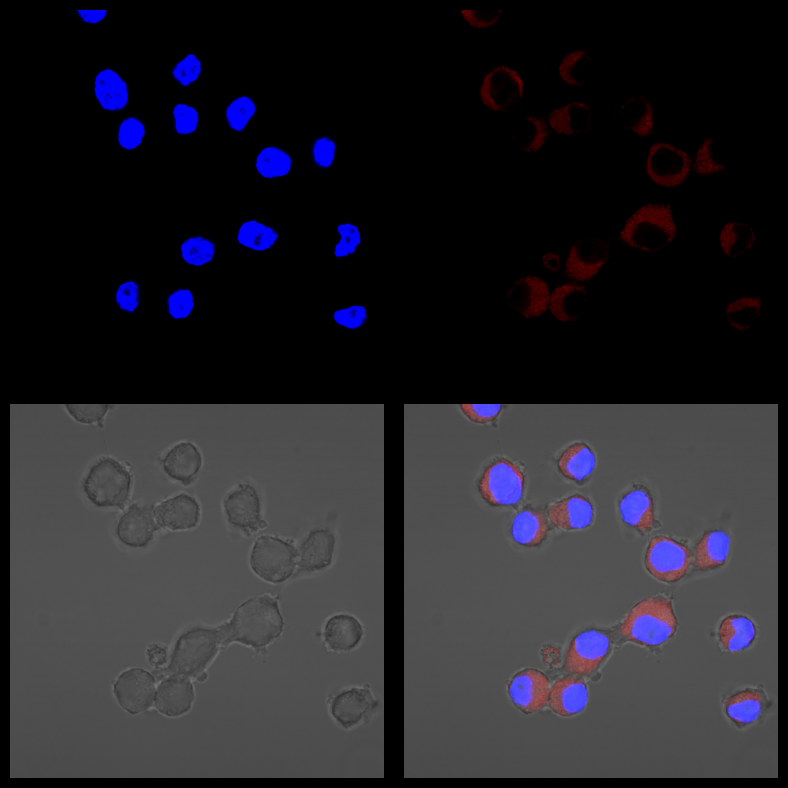

Supplement: Supplementary file 15 — Source Data Fig. EV3 [file 44318_2024_270_MOESM15_ESM.zip › EV3/Figure EV3C/LV-control+EB 100.bmp]

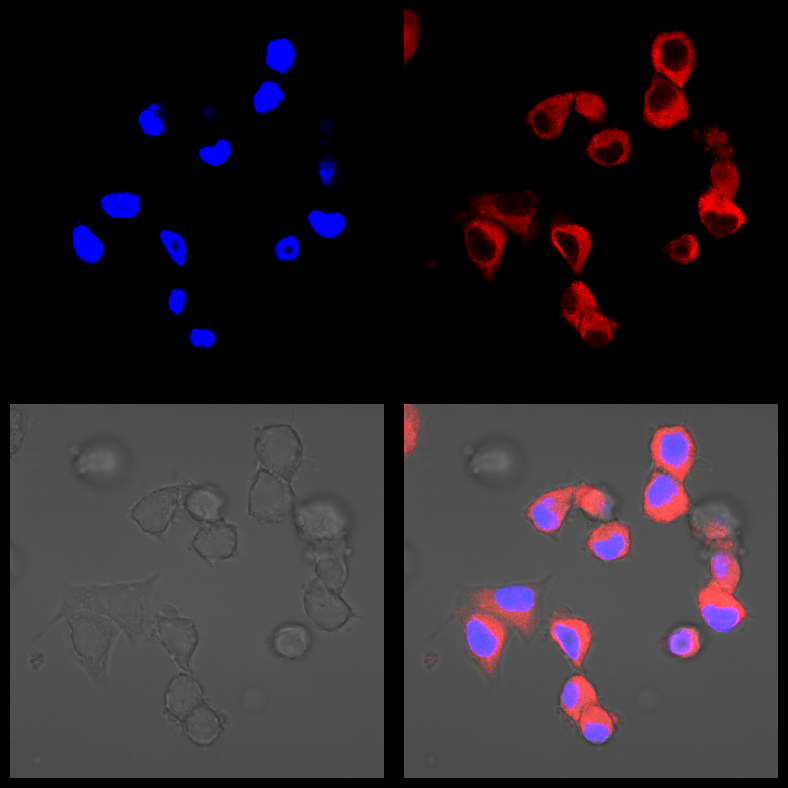

Supplement: Supplementary file 15 — Source Data Fig. EV3 [file 44318_2024_270_MOESM15_ESM.zip › EV3/Figure EV3C/LV-lncMtDloop+EB 100.bmp]
