# Supplementary figures and images for: The mitochondrial long non-coding RNA lncMtloop regulates mitochondrial transcription and suppresses Alzheimer’s disease (part 2 of 2)
Source: EMBO J. 2024 Oct 18;43(23):6001–31. doi: 10.1038/s44318-024-00270-7 (PMC11612450; doi:10.1038/s44318-024-00270-7)

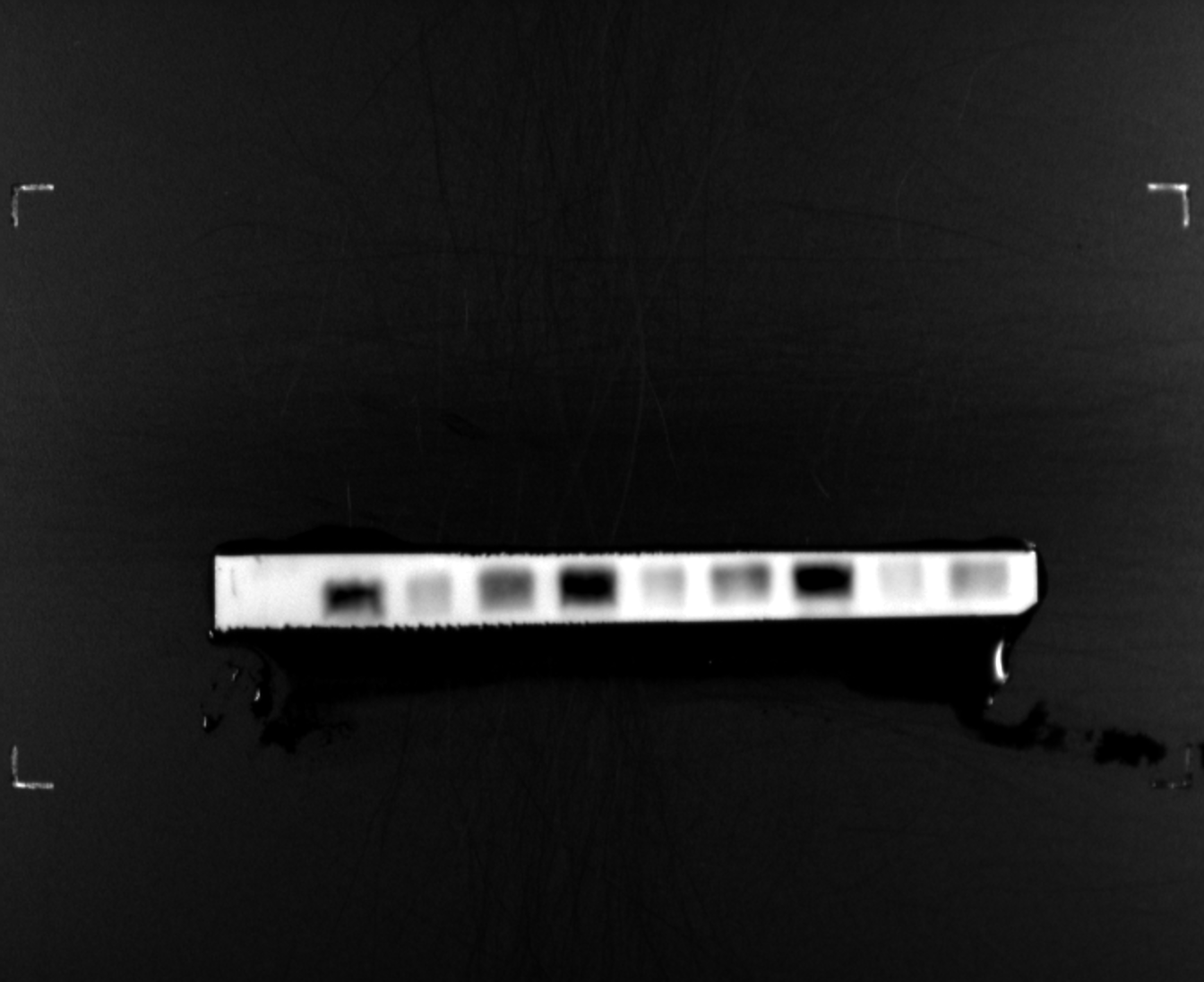

Supplement: Supplementary file 15 — Source Data Fig. EV3 [file 44318_2024_270_MOESM15_ESM.zip › EV3/Figure EV3E/Compex IV.Tif]

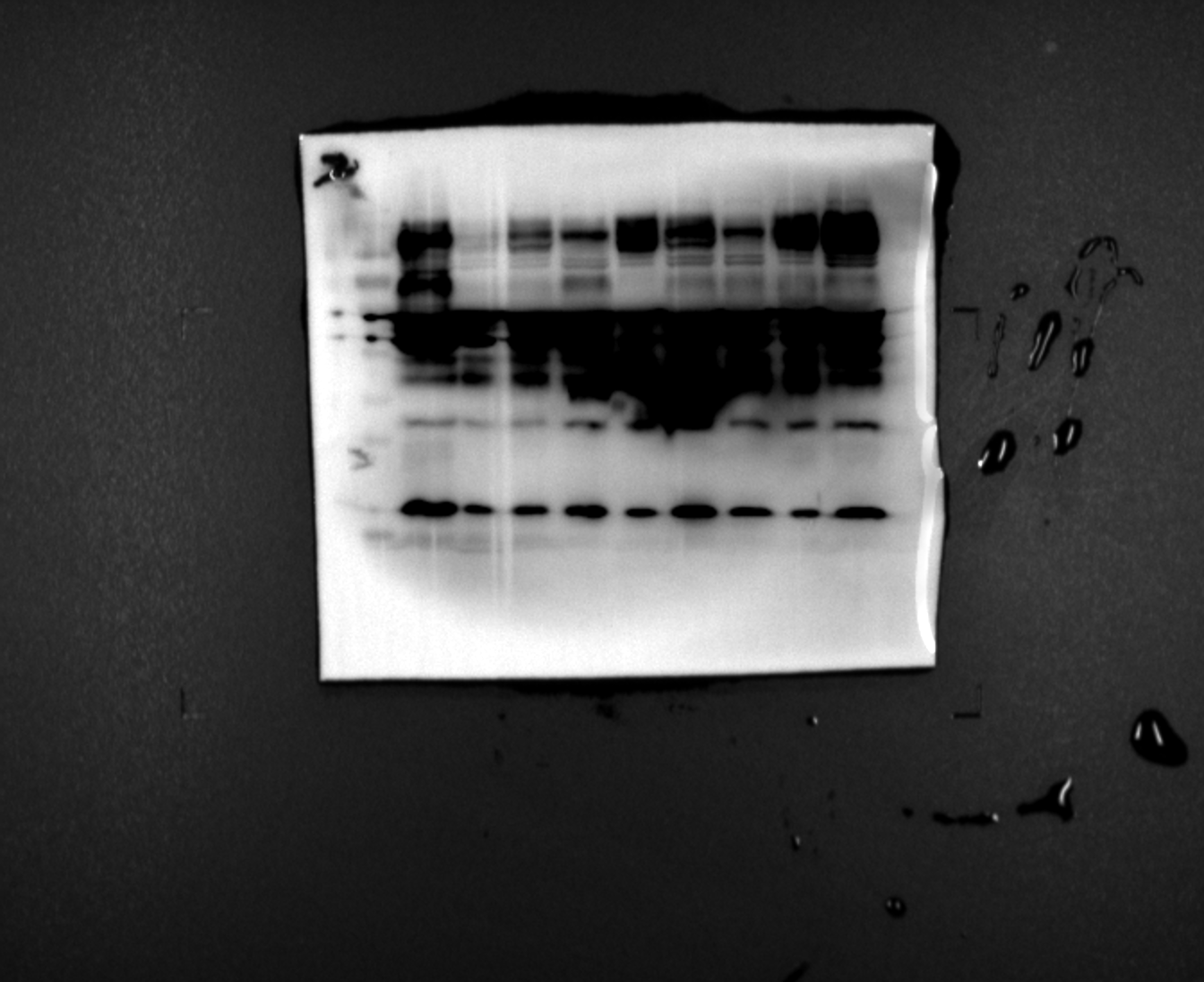

Supplement: Supplementary file 15 — Source Data Fig. EV3 [file 44318_2024_270_MOESM15_ESM.zip › EV3/Figure EV3E/Complex IV-II-I.Tif]

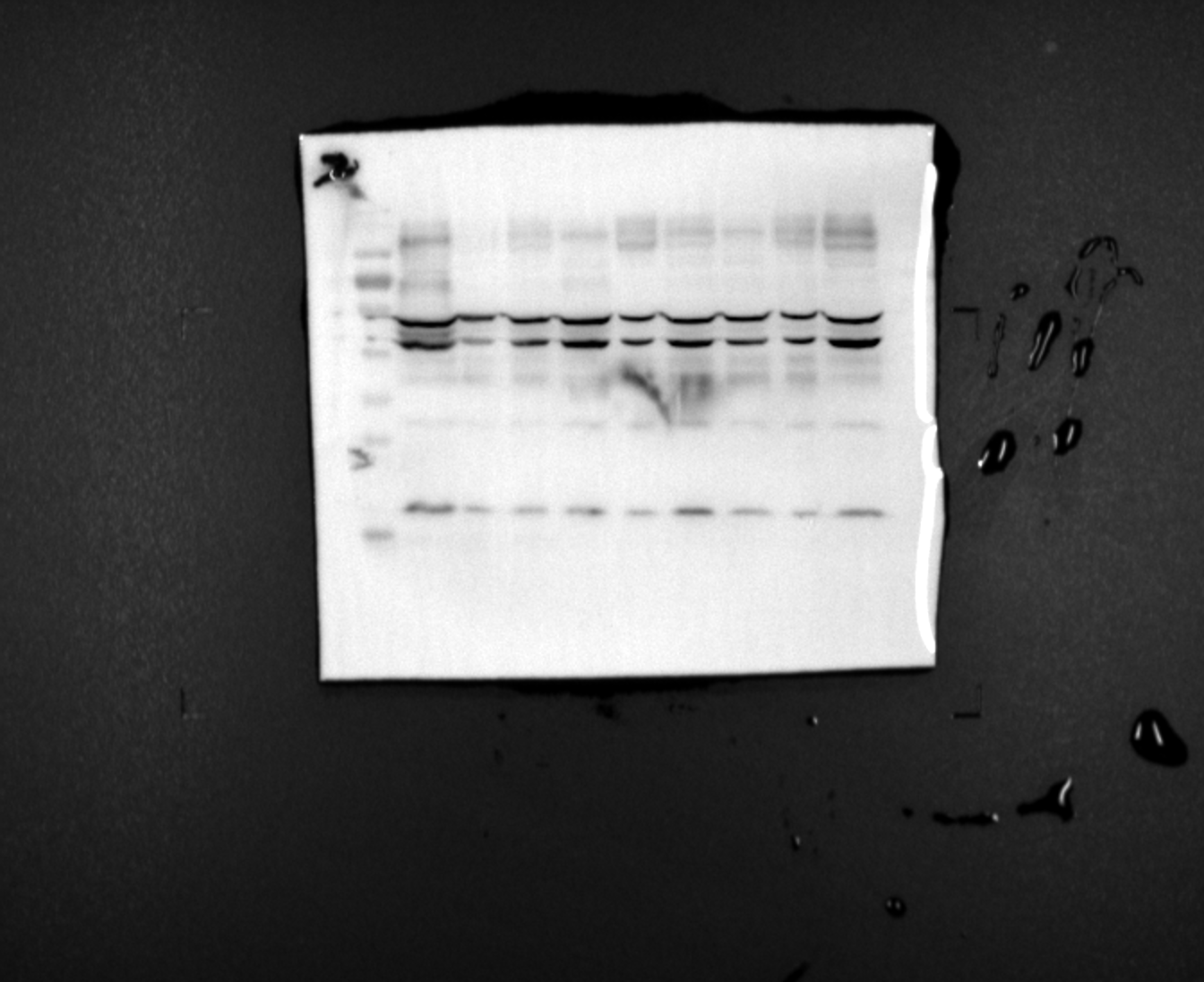

Supplement: Supplementary file 15 — Source Data Fig. EV3 [file 44318_2024_270_MOESM15_ESM.zip › EV3/Figure EV3E/Complex V-III.Tif]

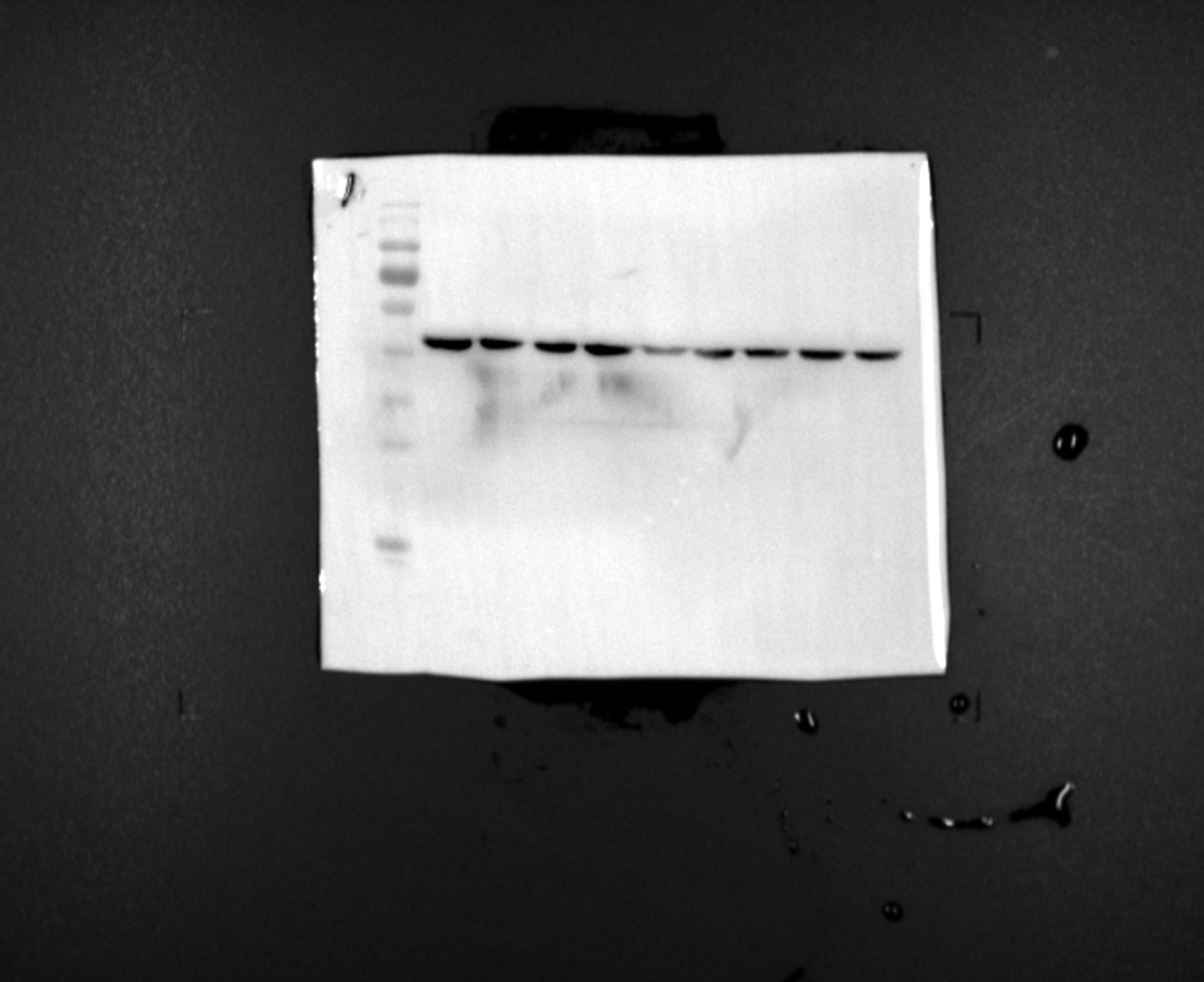

Supplement: Supplementary file 15 — Source Data Fig. EV3 [file 44318_2024_270_MOESM15_ESM.zip › EV3/Figure EV3E/a┬-actin.Tif]

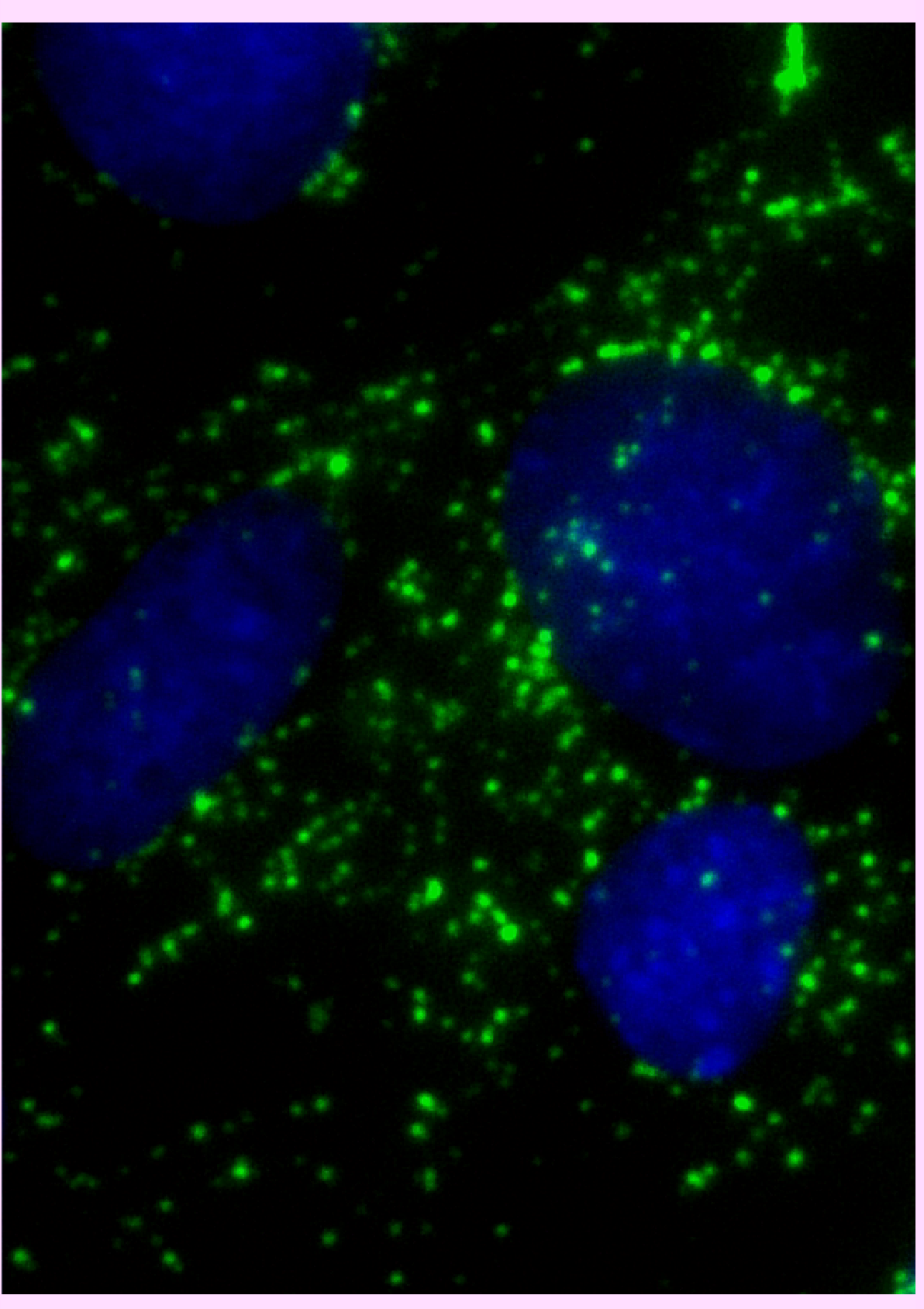

Supplement: Supplementary file 16 — Source Data Fig. EV5 [file 44318_2024_270_MOESM16_ESM.zip › EV5/Figure EV5A/lncMtDloop_3xTg_DIV14.tif]

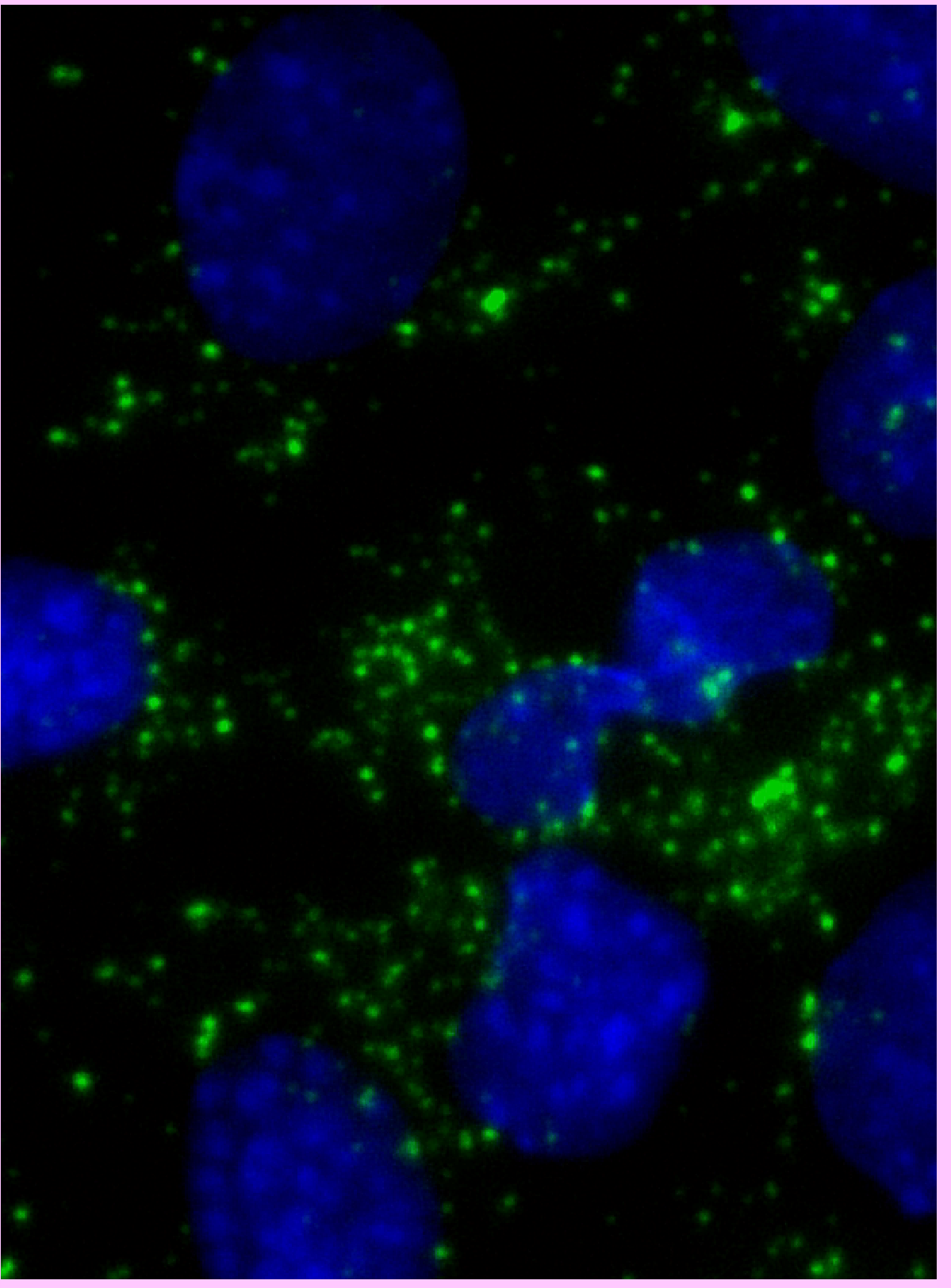

Supplement: Supplementary file 16 — Source Data Fig. EV5 [file 44318_2024_270_MOESM16_ESM.zip › EV5/Figure EV5A/lncMtDloop_3xTg_DIV21.tif]

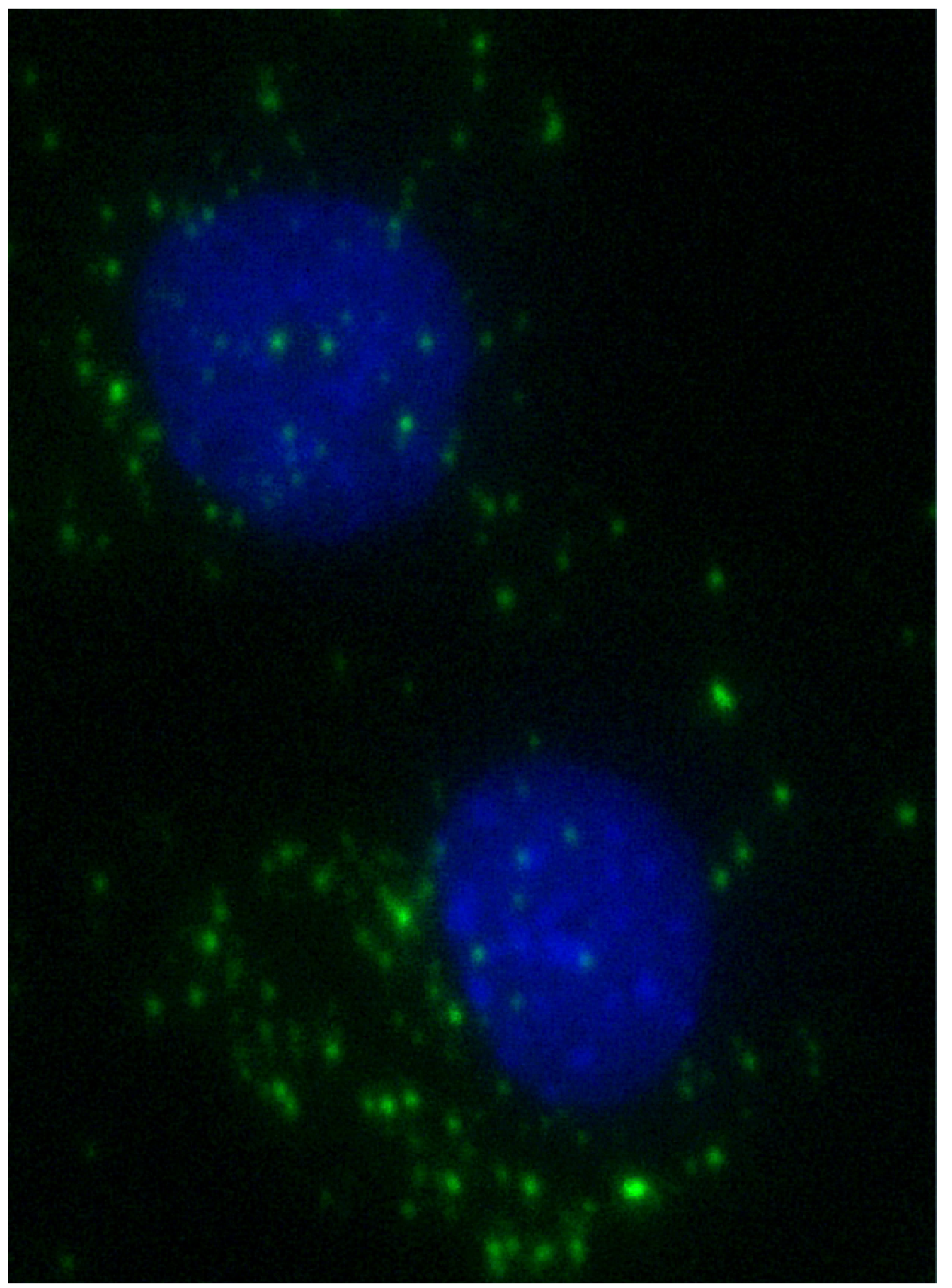

Supplement: Supplementary file 16 — Source Data Fig. EV5 [file 44318_2024_270_MOESM16_ESM.zip › EV5/Figure EV5A/lncMtDloop_WT_Abeta_DIV14.tif]

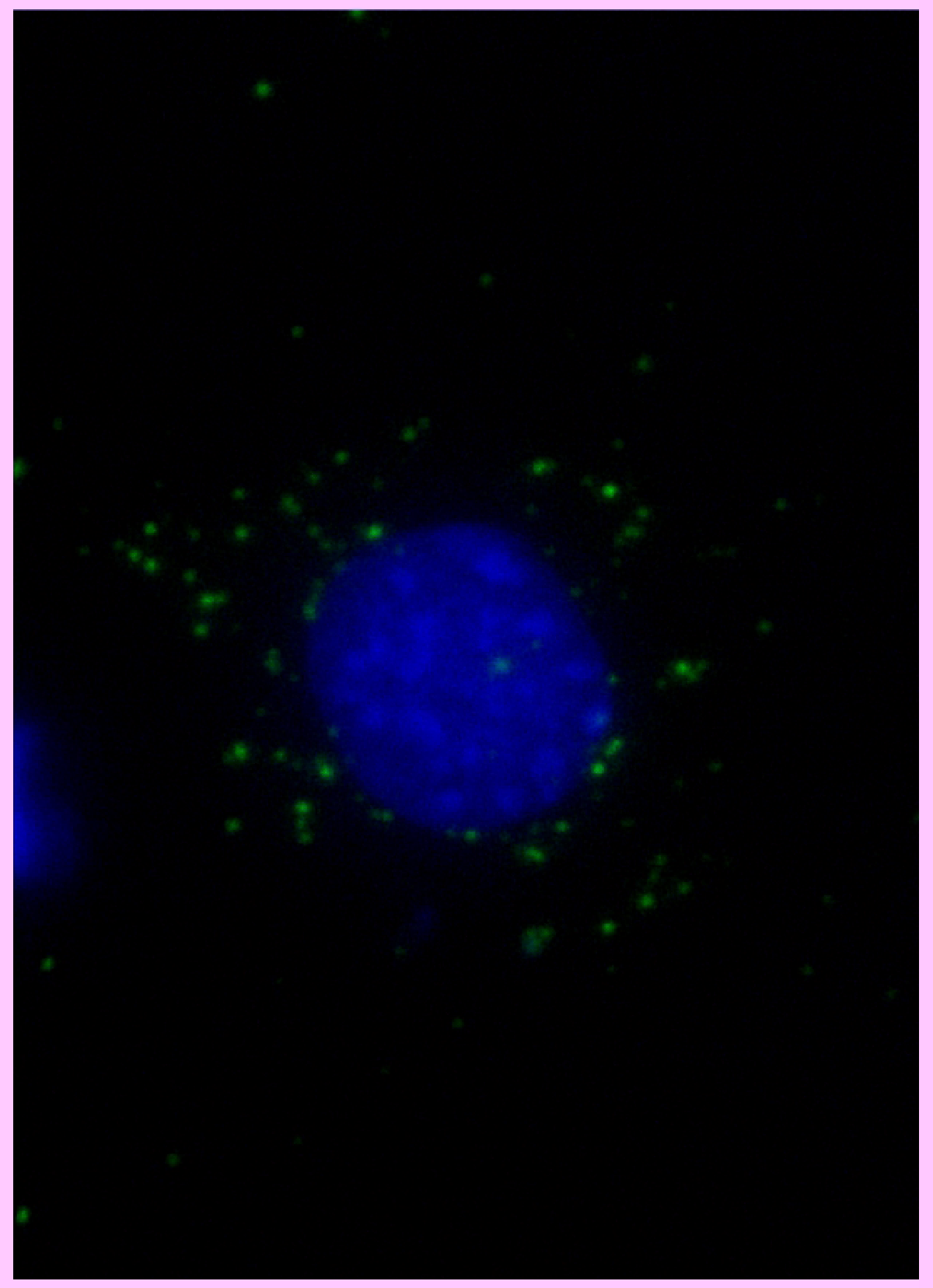

Supplement: Supplementary file 16 — Source Data Fig. EV5 [file 44318_2024_270_MOESM16_ESM.zip › EV5/Figure EV5A/lncMtDloop_WT_Abeta_DIV21.tif]

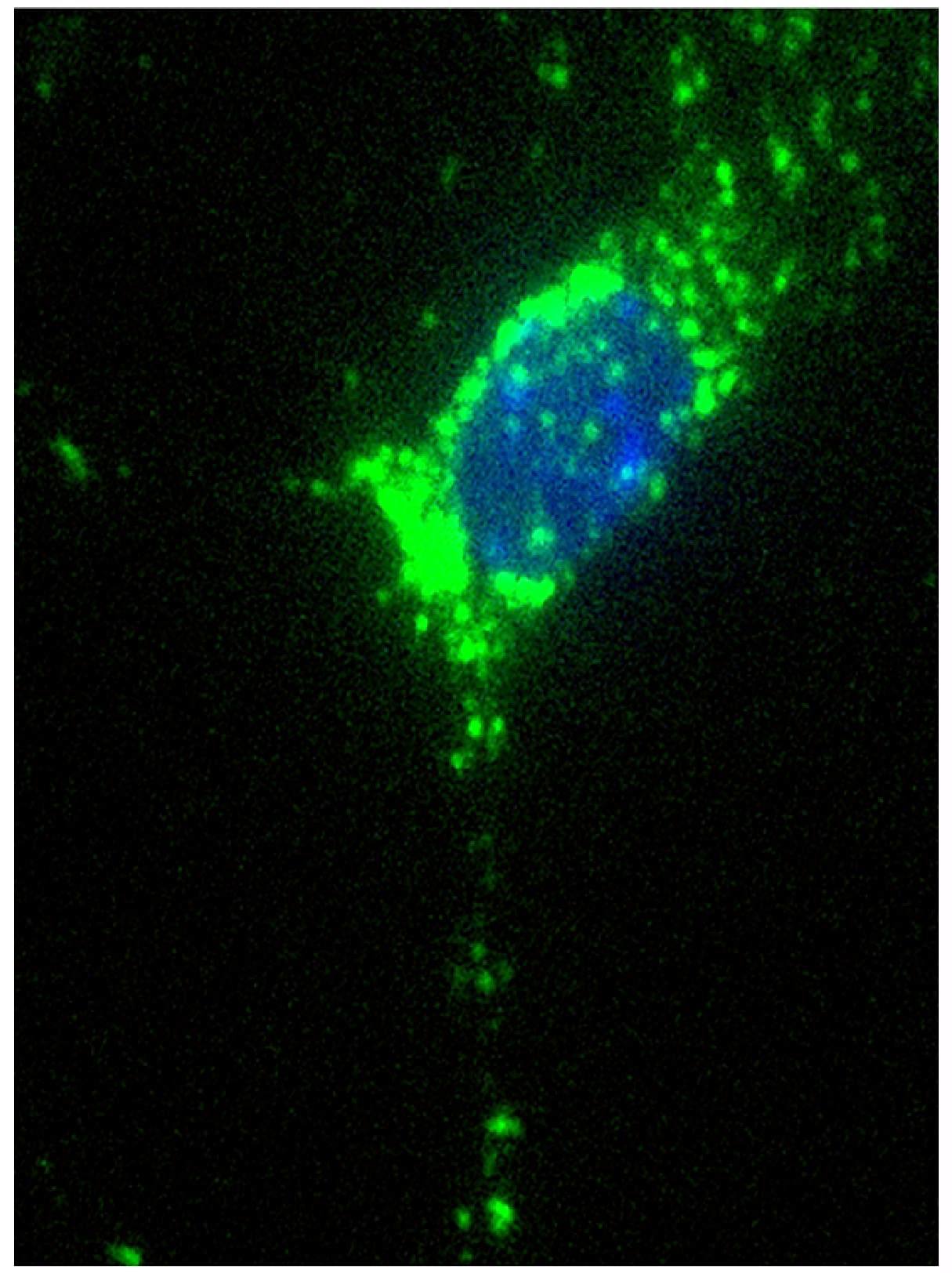

Supplement: Supplementary file 16 — Source Data Fig. EV5 [file 44318_2024_270_MOESM16_ESM.zip › EV5/Figure EV5A/lncMtDloop_WT_DIV14.tif]

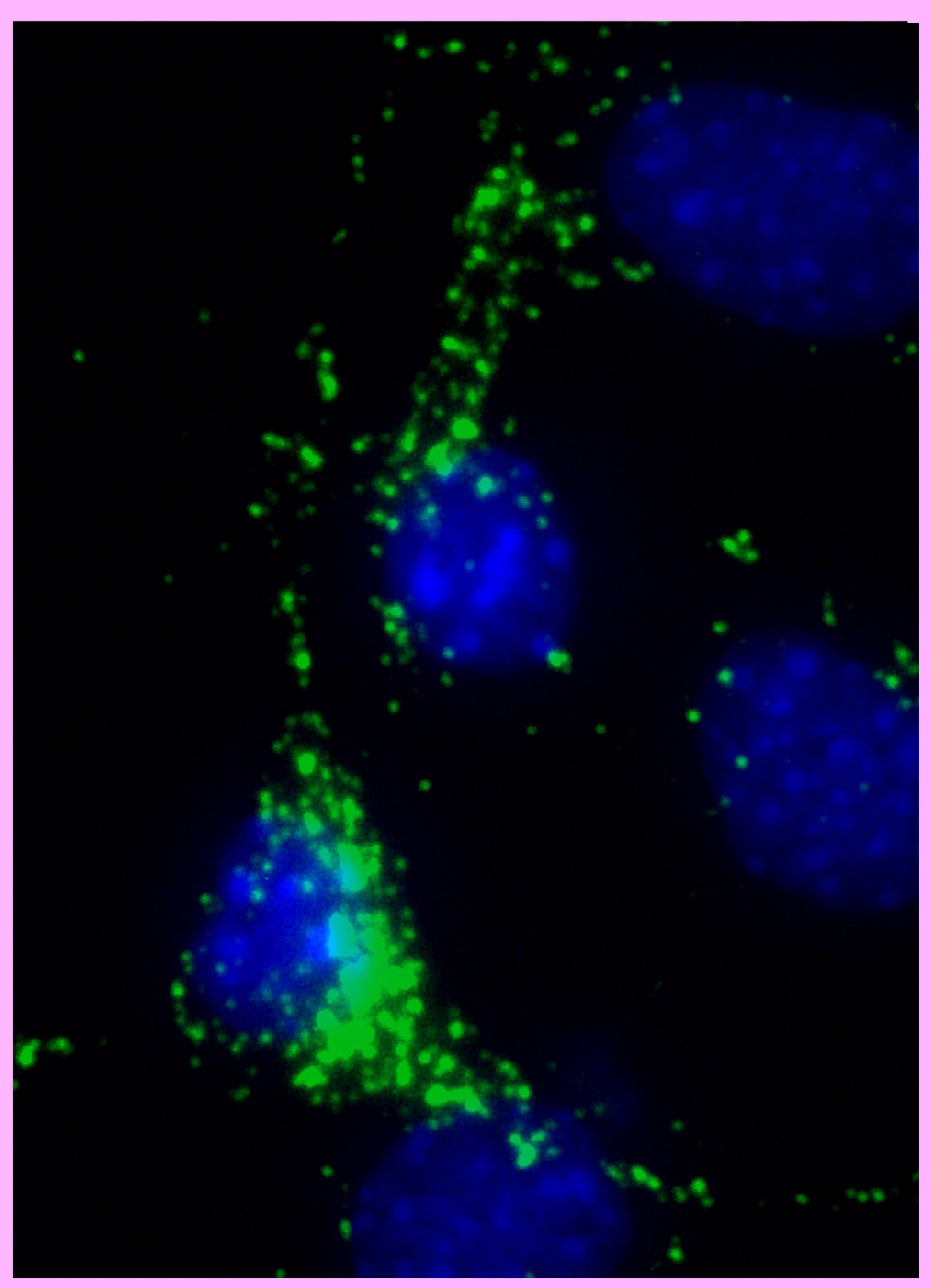

Supplement: Supplementary file 16 — Source Data Fig. EV5 [file 44318_2024_270_MOESM16_ESM.zip › EV5/Figure EV5A/lncMtDloop_WT_DIV21.tif]

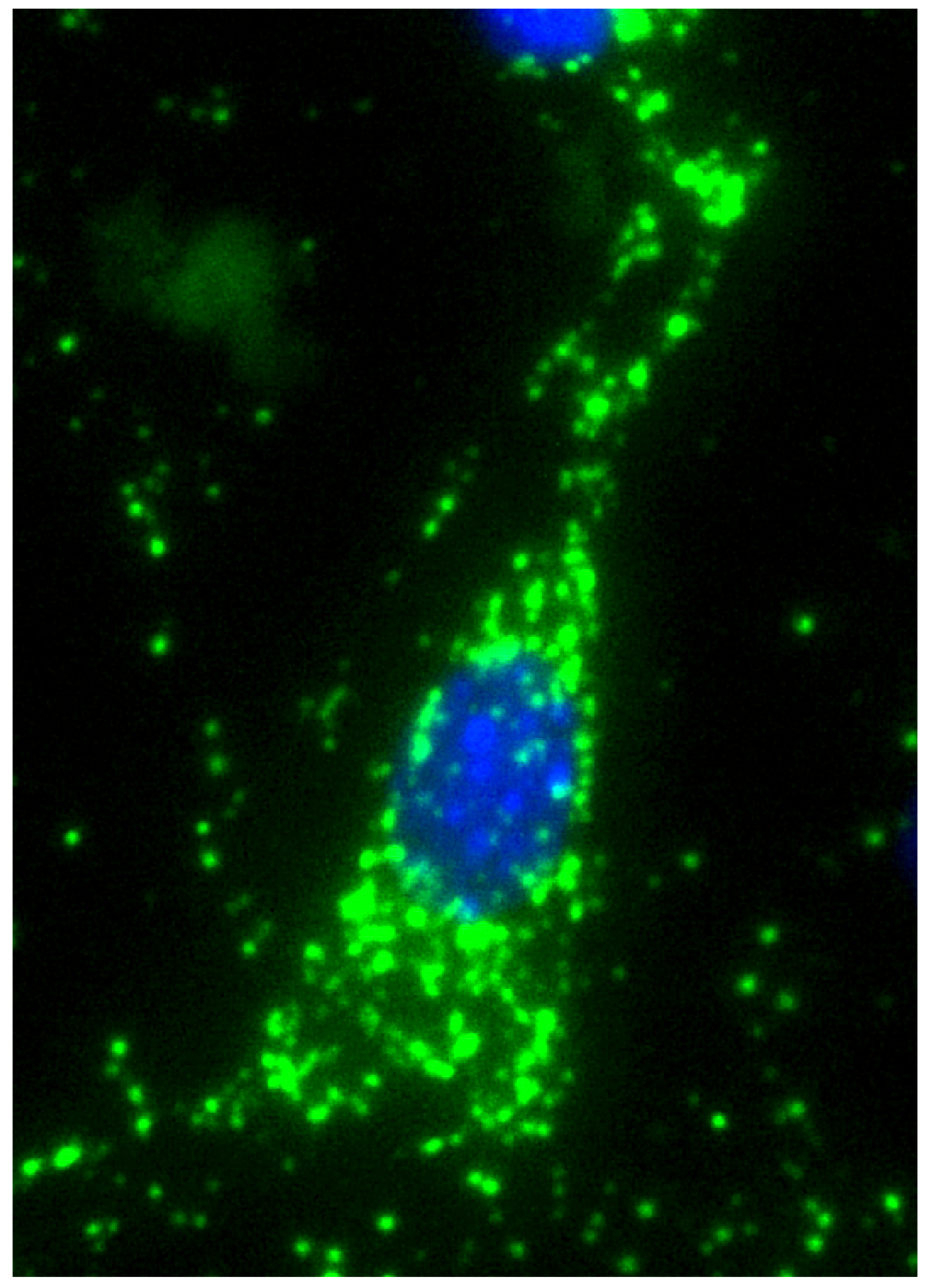

Supplement: Supplementary file 16 — Source Data Fig. EV5 [file 44318_2024_270_MOESM16_ESM.zip › EV5/Figure EV5A/lncMtDloop_WT_vehicle_DIV14.tif]

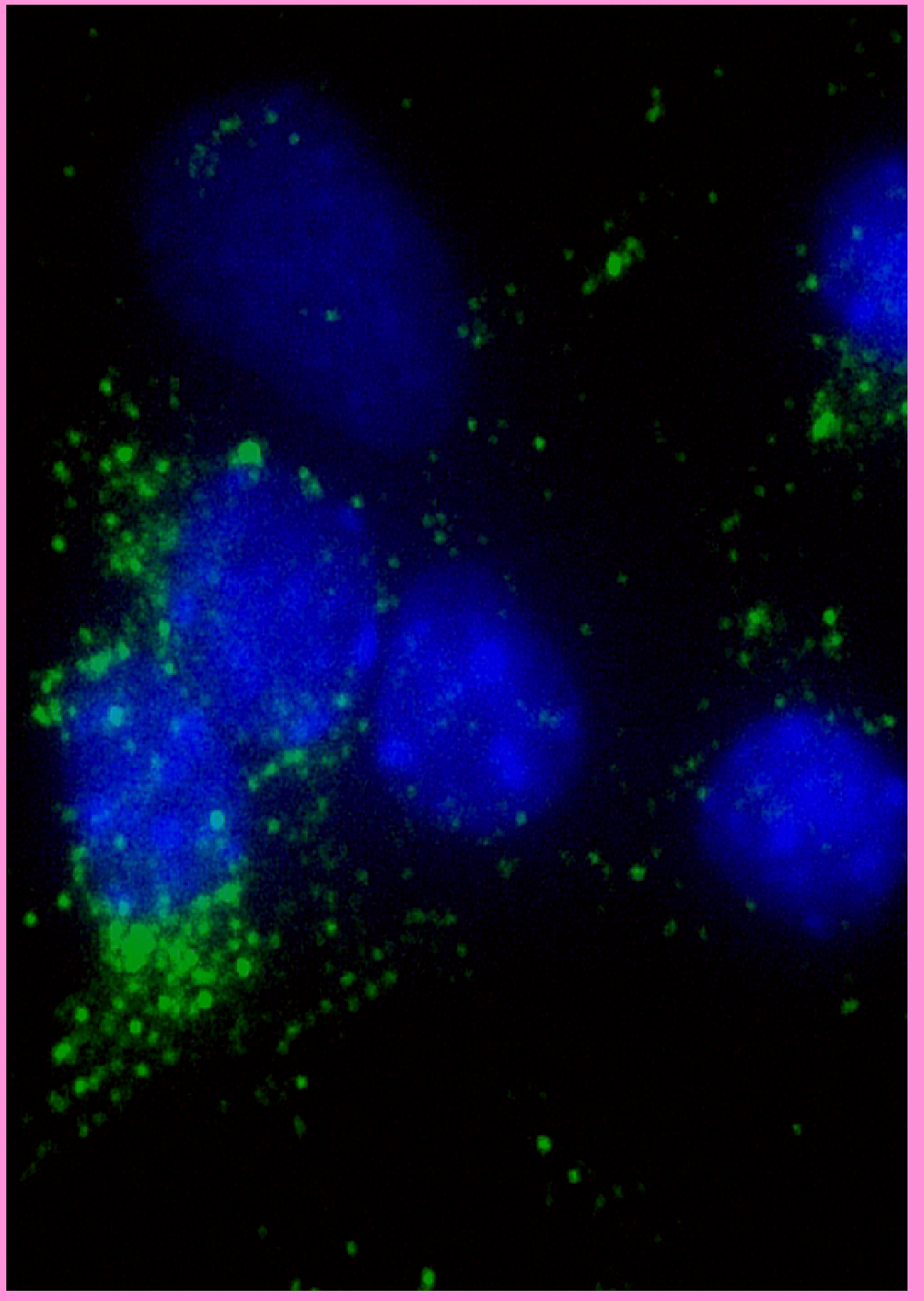

Supplement: Supplementary file 16 — Source Data Fig. EV5 [file 44318_2024_270_MOESM16_ESM.zip › EV5/Figure EV5A/lncMtDloop_WT_vehicle_DIV21.tif]

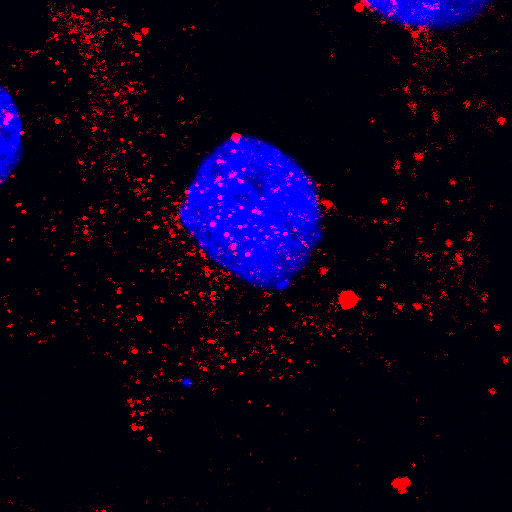

Supplement: Supplementary file 16 — Source Data Fig. EV5 [file 44318_2024_270_MOESM16_ESM.zip › EV5/Figure EV5D/2.tif]

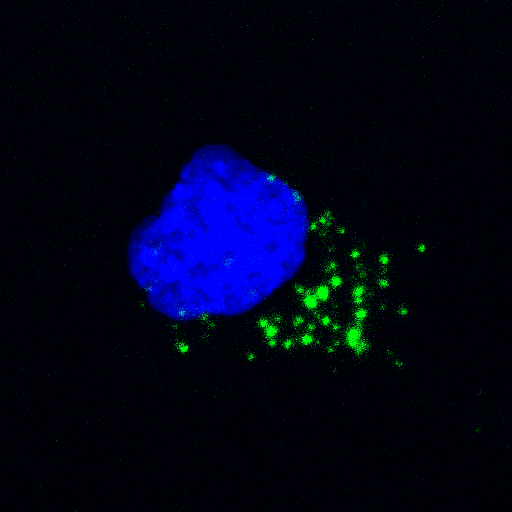

Supplement: Supplementary file 16 — Source Data Fig. EV5 [file 44318_2024_270_MOESM16_ESM.zip › EV5/Figure EV5D/lnc+ab ad(004-039)c1qbp(41-540_Image008.tif]

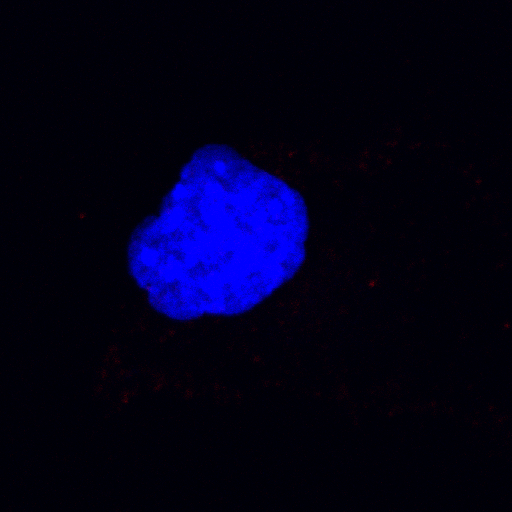

Supplement: Supplementary file 16 — Source Data Fig. EV5 [file 44318_2024_270_MOESM16_ESM.zip › EV5/Figure EV5D/lnc+ab ad(004-039)c1qbp(41-540_Image0081.tif]

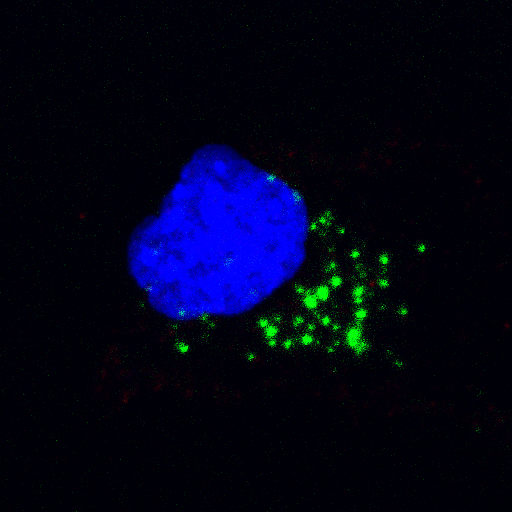

Supplement: Supplementary file 16 — Source Data Fig. EV5 [file 44318_2024_270_MOESM16_ESM.zip › EV5/Figure EV5D/lnc+ab ad(004-039)c1qbp(41-540_Image0082.tif]

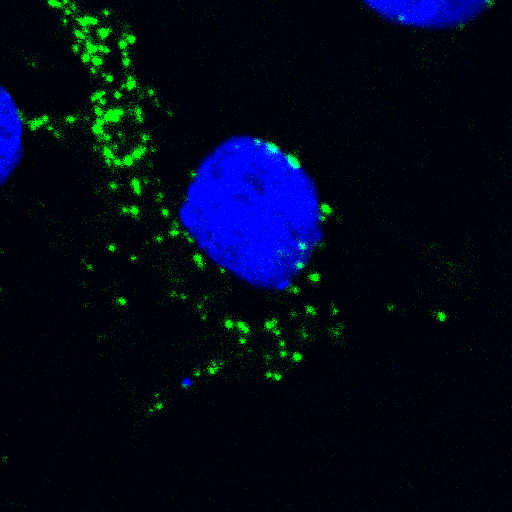

Supplement: Supplementary file 16 — Source Data Fig. EV5 [file 44318_2024_270_MOESM16_ESM.zip › EV5/Figure EV5D/lnc+ab ad(004-039)c1qbp(41-540_Image033.tif]

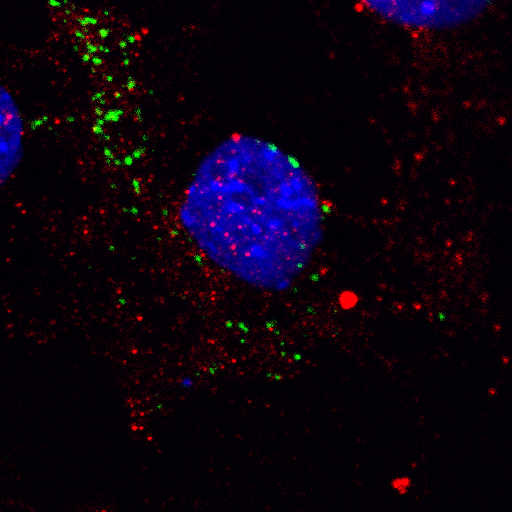

Supplement: Supplementary file 16 — Source Data Fig. EV5 [file 44318_2024_270_MOESM16_ESM.zip › EV5/Figure EV5D/lnc+ab ad(004-039)c1qbp(41-540_Image033q.tif]

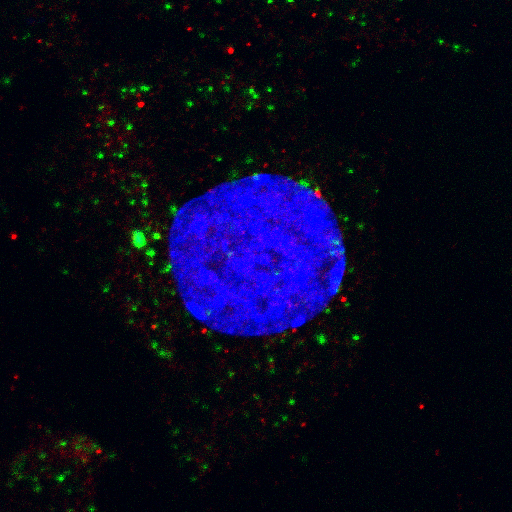

Supplement: Supplementary file 16 — Source Data Fig. EV5 [file 44318_2024_270_MOESM16_ESM.zip › EV5/Figure EV5D/lnc+ab ad(004-039)c1qbp(41-540_Image046.tif]

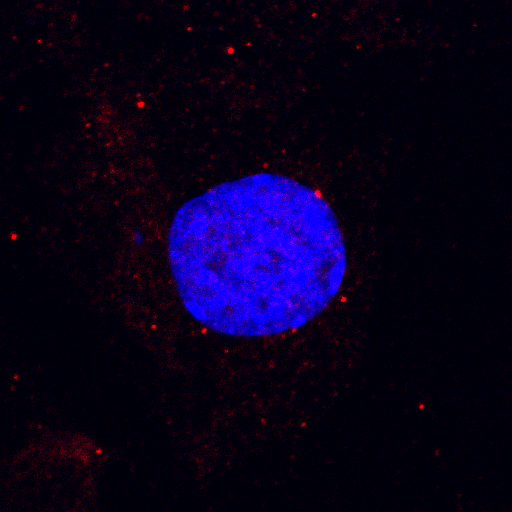

Supplement: Supplementary file 16 — Source Data Fig. EV5 [file 44318_2024_270_MOESM16_ESM.zip › EV5/Figure EV5D/lnc+ab ad(004-039)c1qbp(41-540_Image0461.tif]

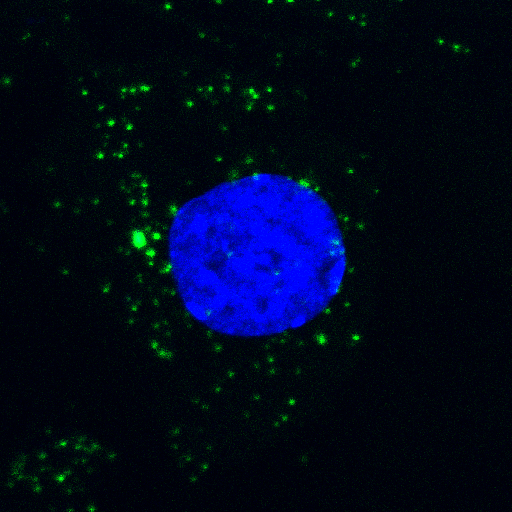

Supplement: Supplementary file 16 — Source Data Fig. EV5 [file 44318_2024_270_MOESM16_ESM.zip › EV5/Figure EV5D/lnc+ab ad(004-039)c1qbp(41-540_Image0462.tif]

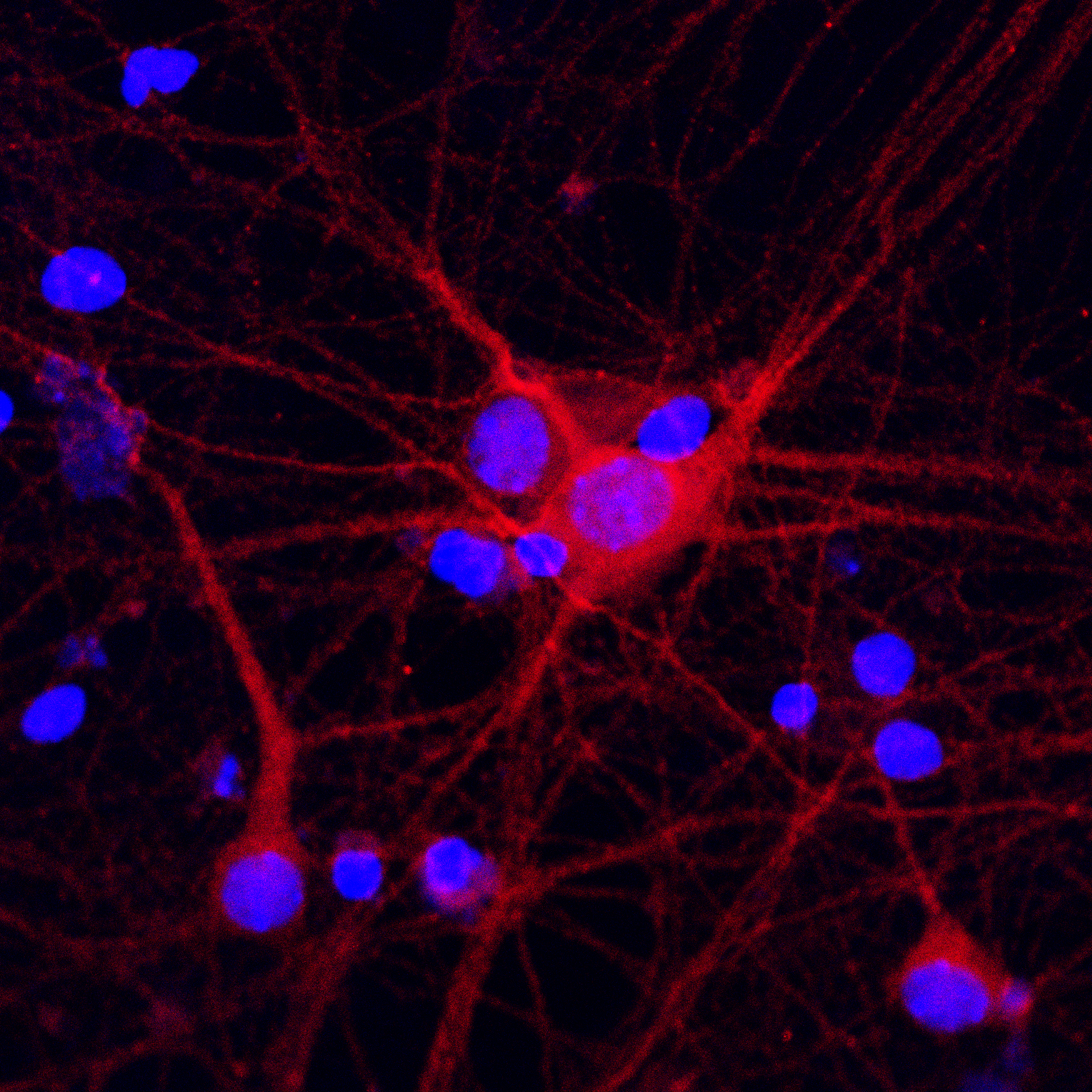

Supplement: Supplementary file 16 — Source Data Fig. EV5 [file 44318_2024_270_MOESM16_ESM.zip › EV5/Figure EV5F/LV-control-3xTg-AT8.tif]

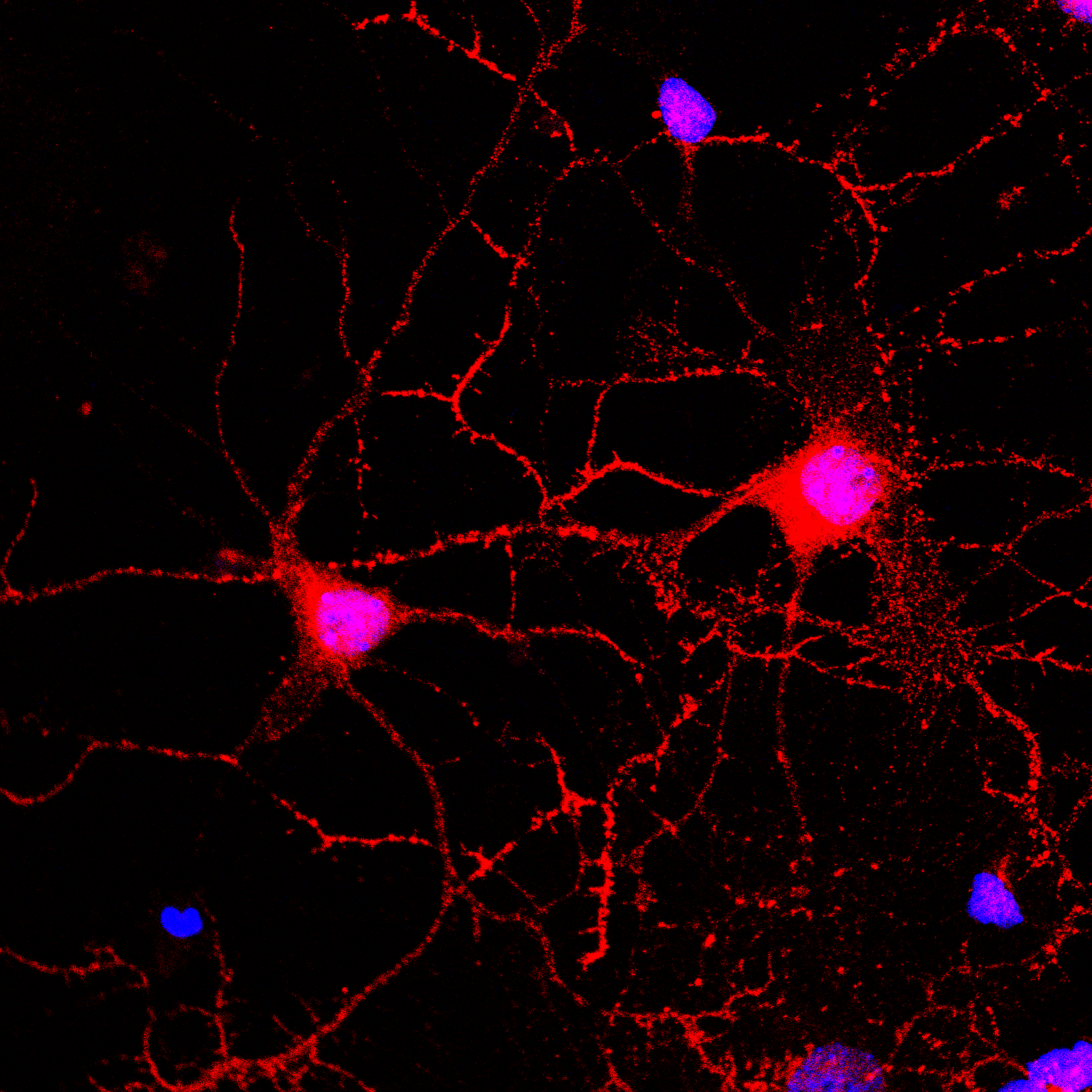

Supplement: Supplementary file 16 — Source Data Fig. EV5 [file 44318_2024_270_MOESM16_ESM.zip › EV5/Figure EV5F/LV-control-3xTg-HT7.tif]

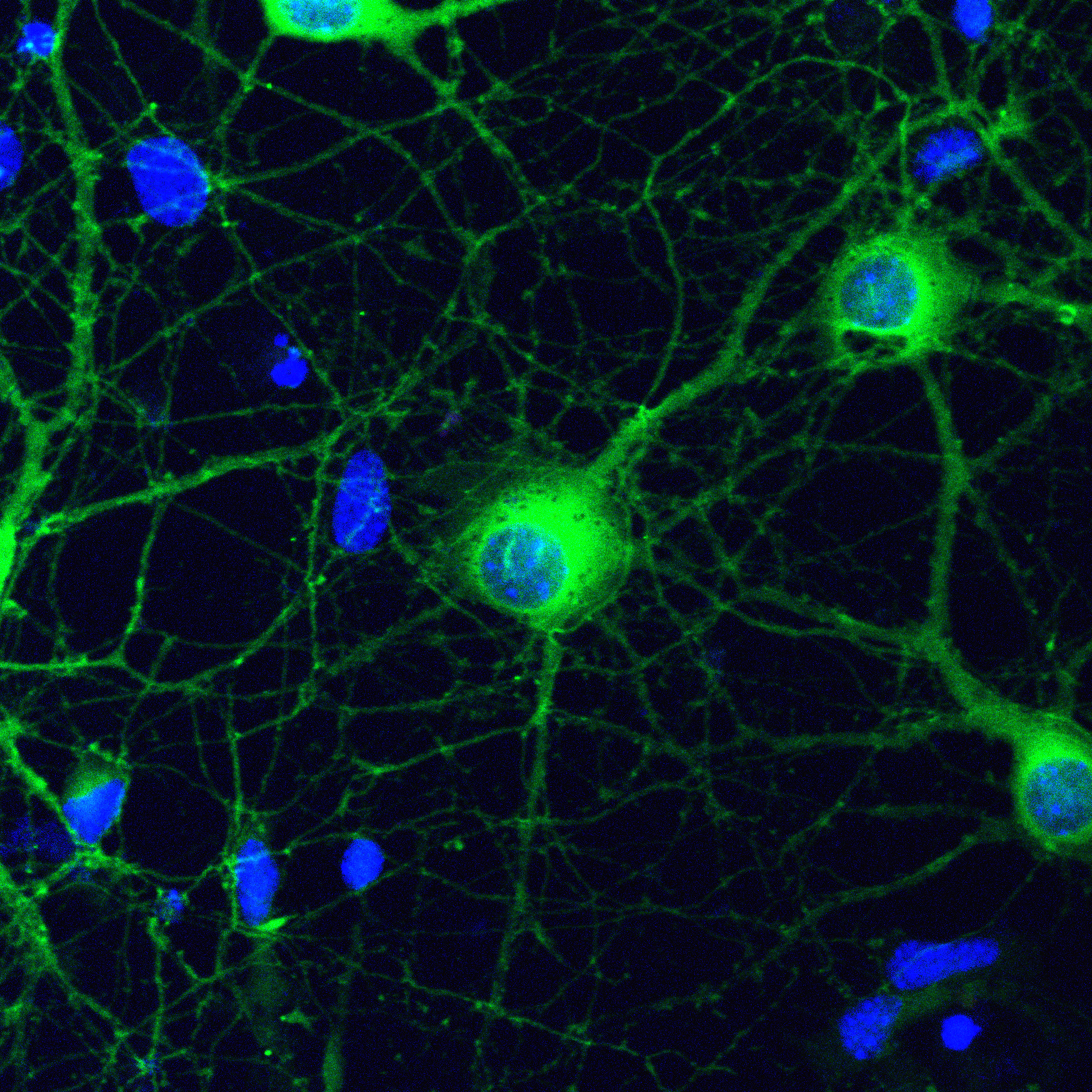

Supplement: Supplementary file 16 — Source Data Fig. EV5 [file 44318_2024_270_MOESM16_ESM.zip › EV5/Figure EV5F/LV-control-3xTg-TAU5.tif]

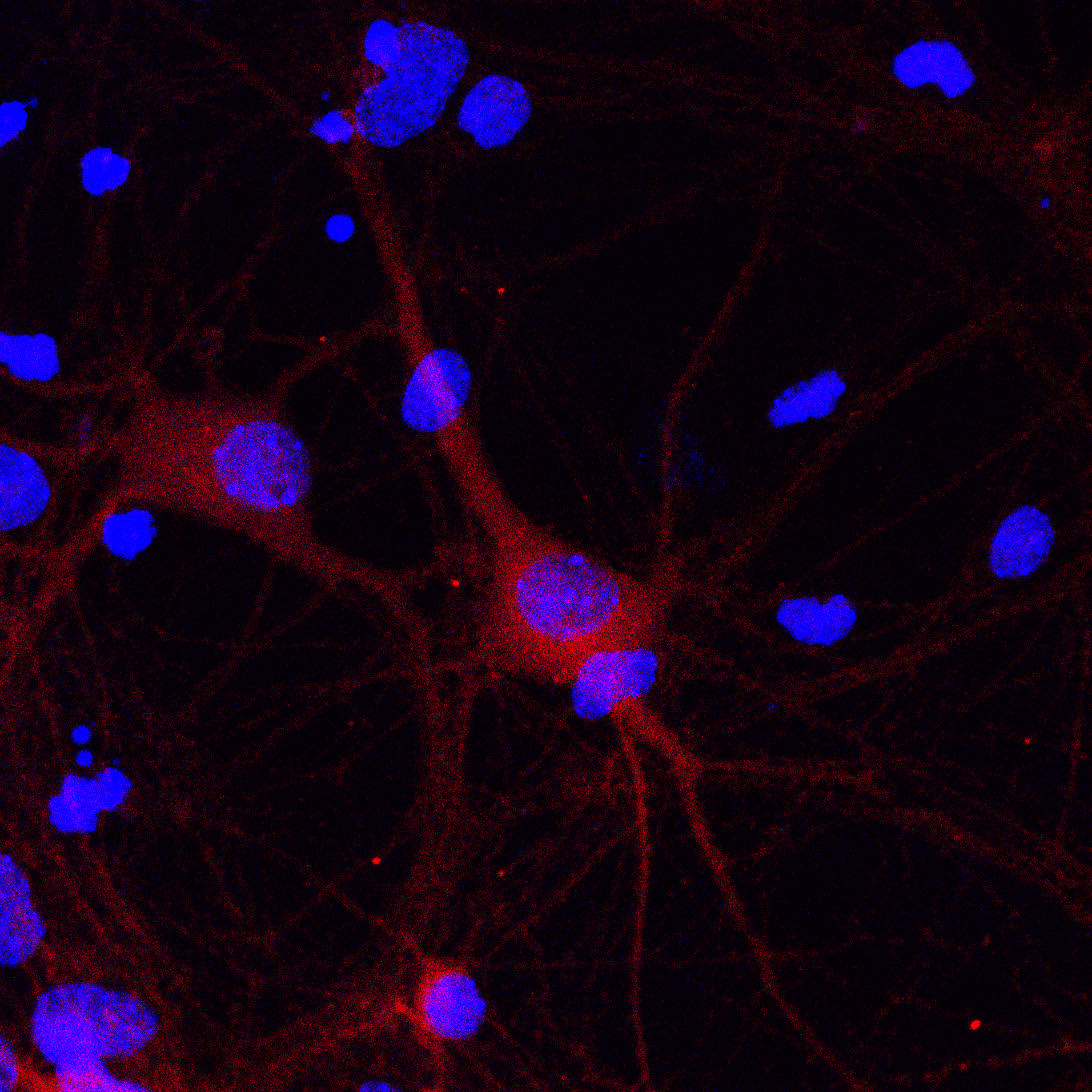

Supplement: Supplementary file 16 — Source Data Fig. EV5 [file 44318_2024_270_MOESM16_ESM.zip › EV5/Figure EV5F/LV-control-WT-AT8.tif]

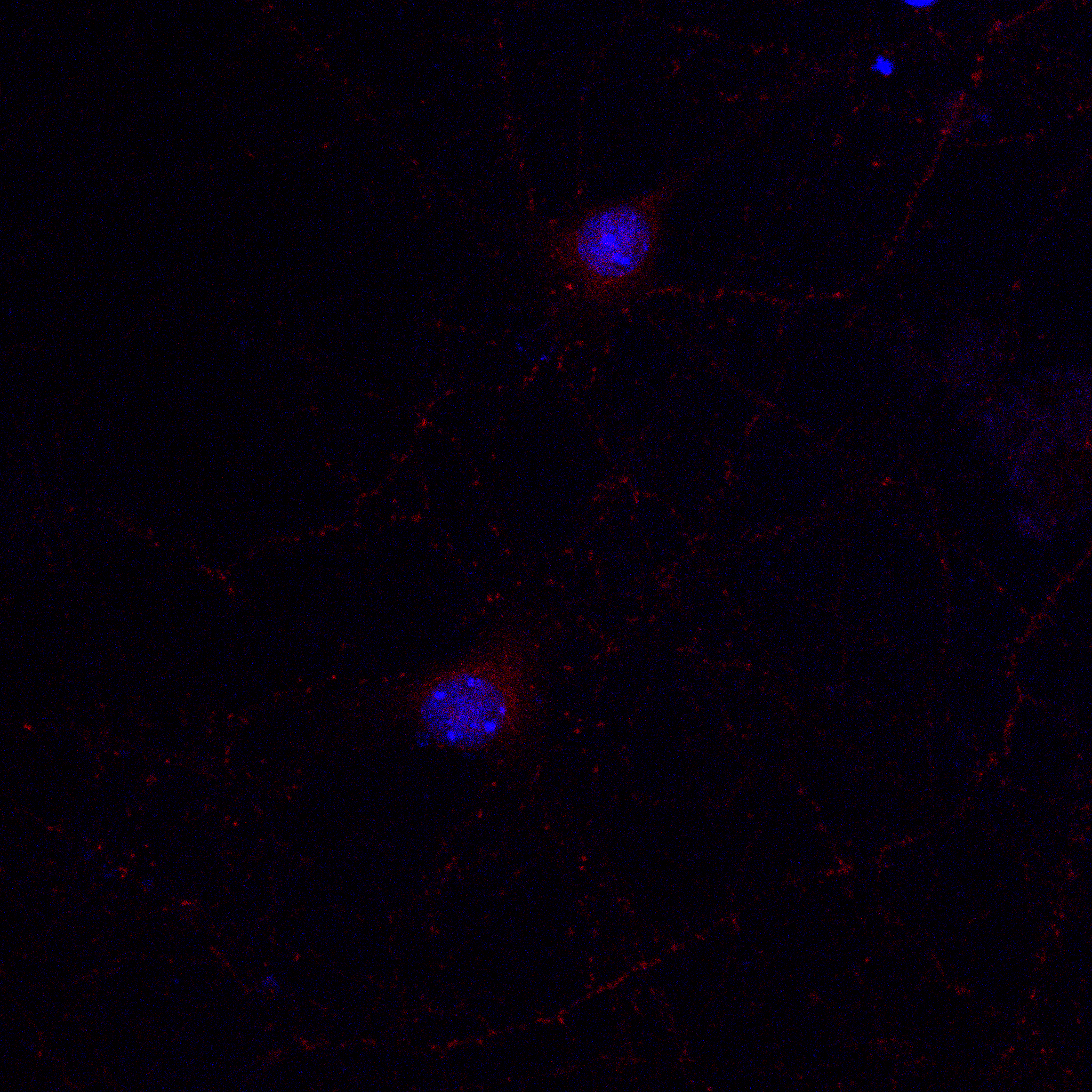

Supplement: Supplementary file 16 — Source Data Fig. EV5 [file 44318_2024_270_MOESM16_ESM.zip › EV5/Figure EV5F/LV-control-WT-HT7.tif]

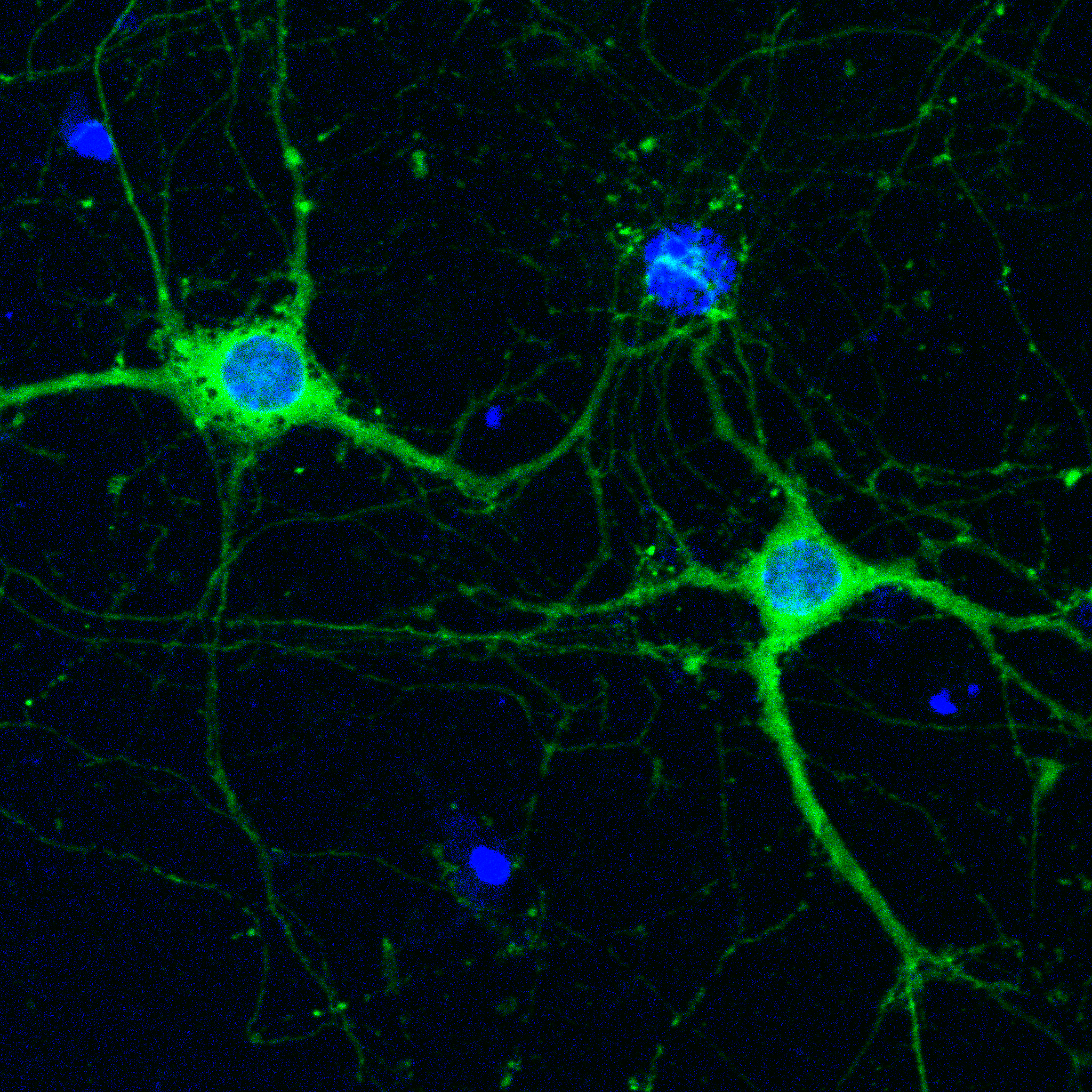

Supplement: Supplementary file 16 — Source Data Fig. EV5 [file 44318_2024_270_MOESM16_ESM.zip › EV5/Figure EV5F/LV-control-WT-TAI5.tif]

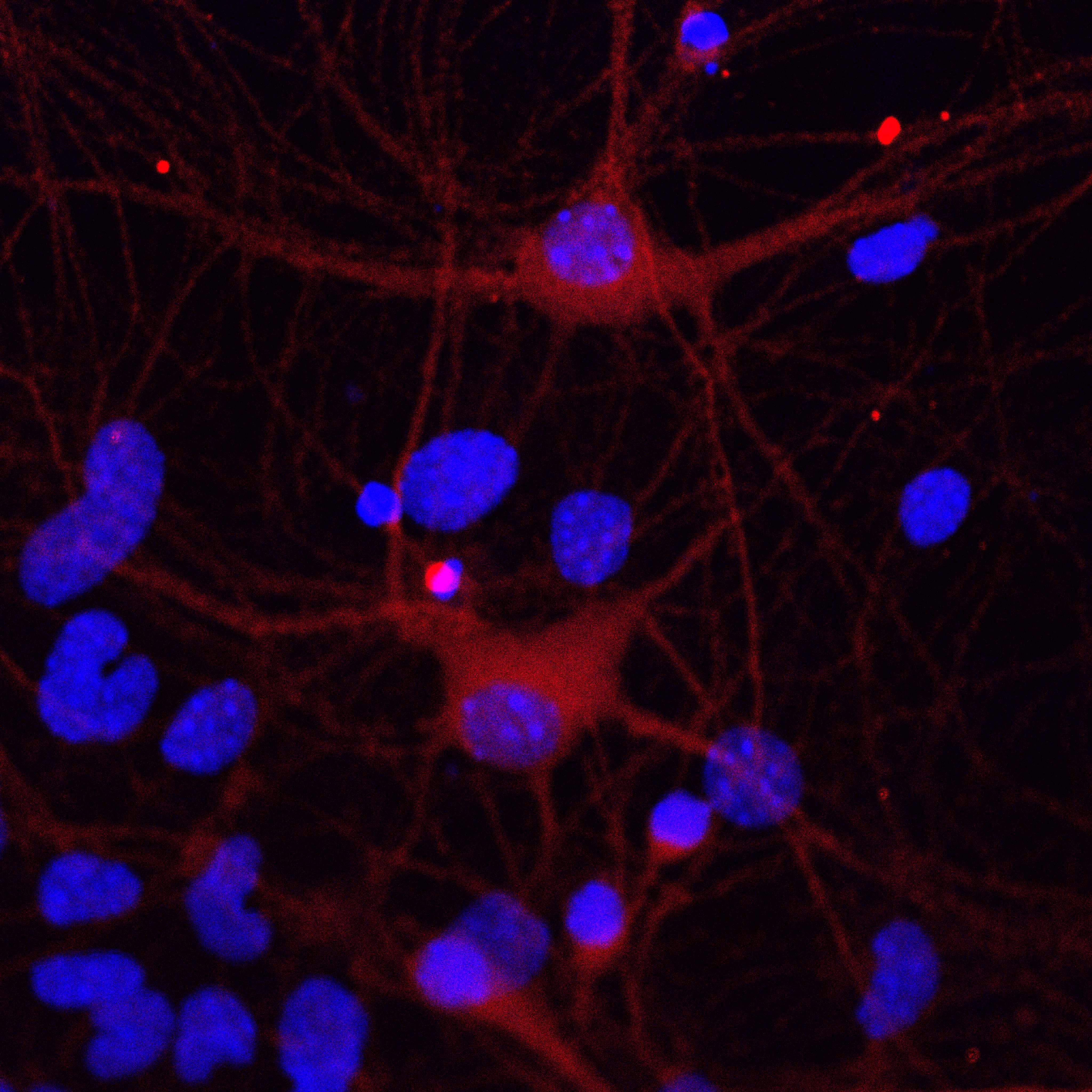

Supplement: Supplementary file 16 — Source Data Fig. EV5 [file 44318_2024_270_MOESM16_ESM.zip › EV5/Figure EV5F/LV-lncMtDloop-3xTg-AT8.tif]

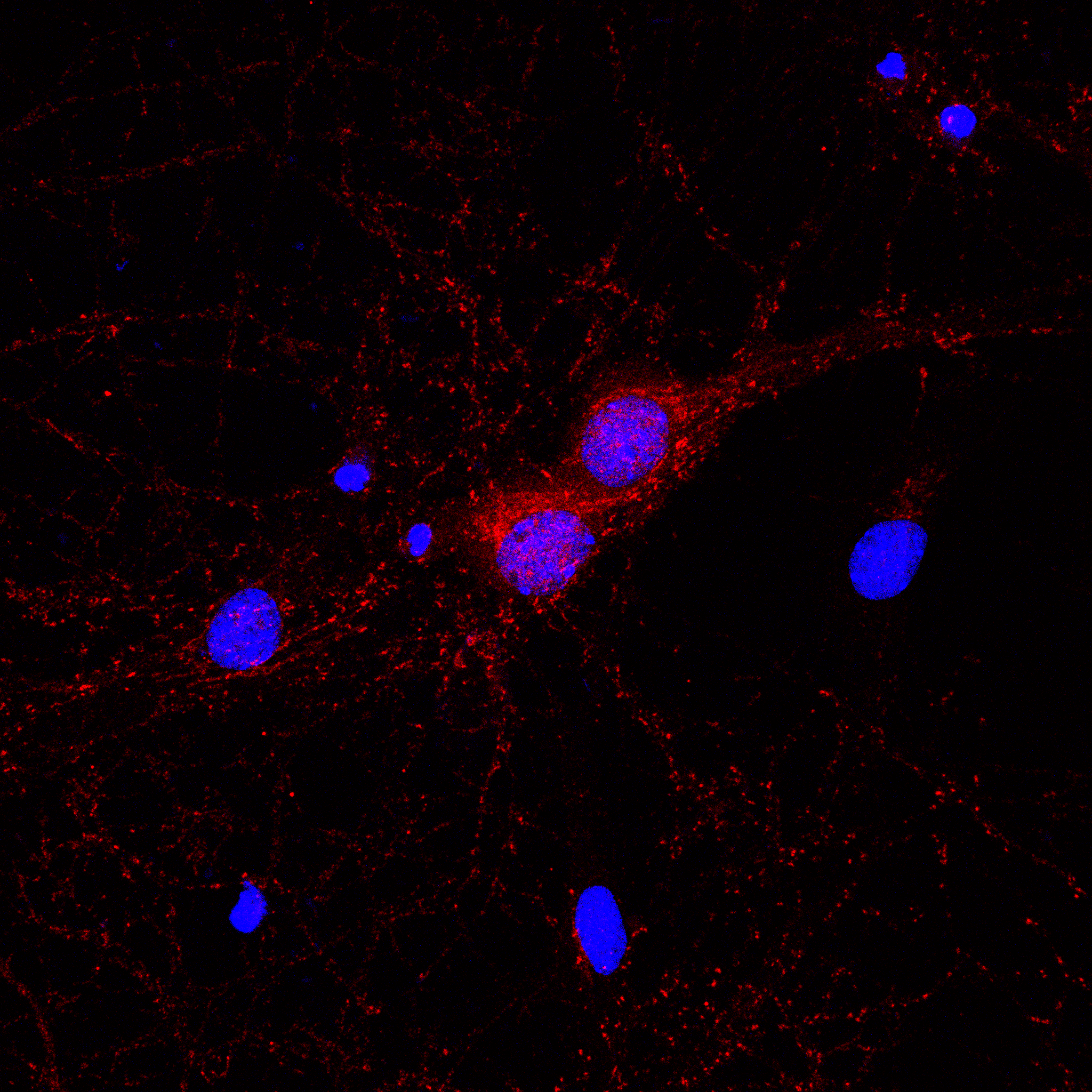

Supplement: Supplementary file 16 — Source Data Fig. EV5 [file 44318_2024_270_MOESM16_ESM.zip › EV5/Figure EV5F/LV-lncMtDloop-3xTg-HT7.tif]

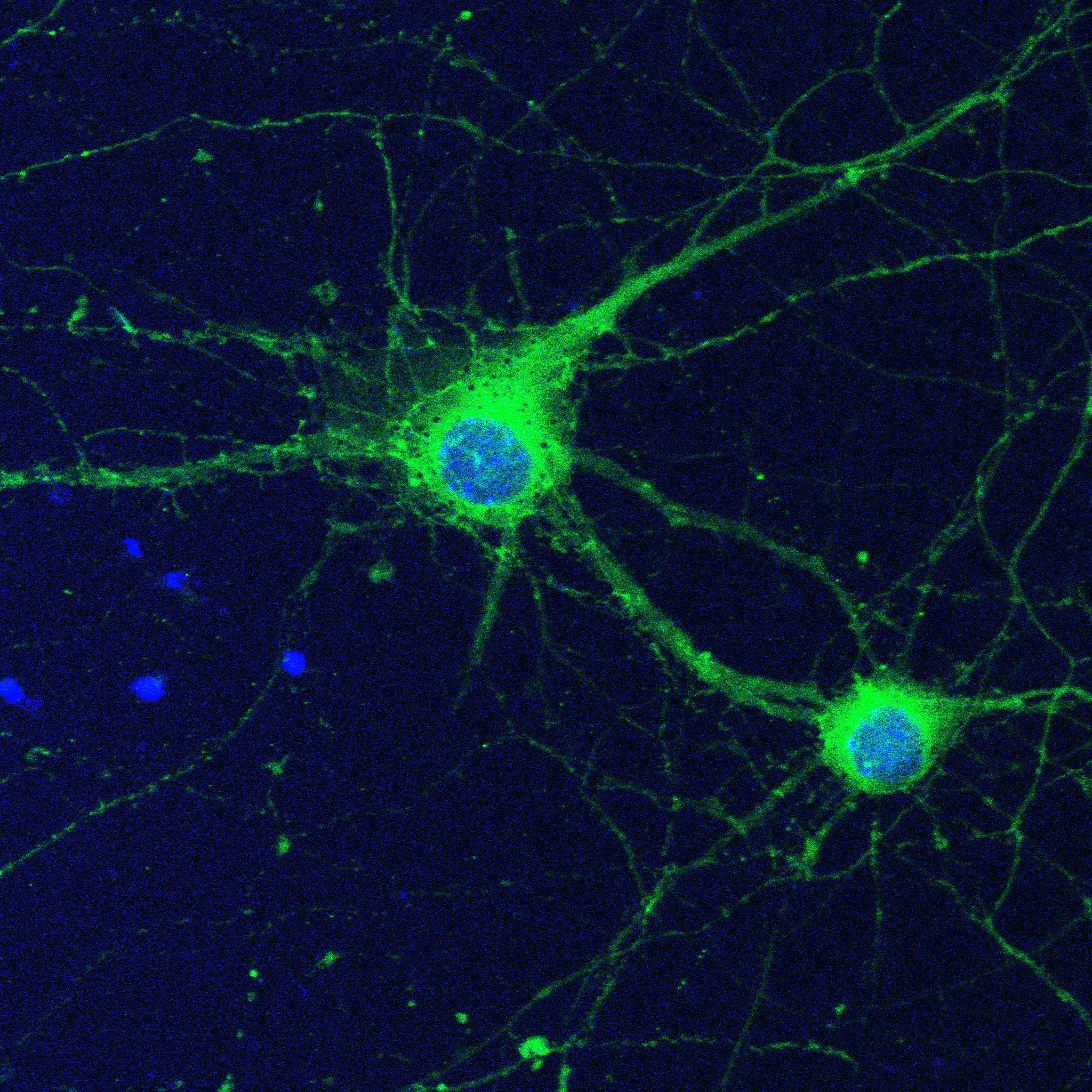

Supplement: Supplementary file 16 — Source Data Fig. EV5 [file 44318_2024_270_MOESM16_ESM.zip › EV5/Figure EV5F/LV-lncMtDloop-3xTg-TAU5.tif]
