# Supplementary material for: The mitochondrial long non-coding RNA lncMtloop regulates mitochondrial transcription and suppresses Alzheimer’s disease
Source: EMBO J. 2024 Oct 18;43(23):6001–31. doi: 10.1038/s44318-024-00270-7 (PMC11612450; doi:10.1038/s44318-024-00270-7)
Supplement: Supplementary file 17 — Expanded View Figures [file 44318_2024_270_MOESM17_ESM.pdf]

Expanded View Figures

Figure EV1. *LncMtDloop* is an evolutionally conserved mitochondrial lncRNA, related to Fig. 1.

(**A**) BLAST search and mapping of this sequence with the NONCODE and UCSC databases affirms that *lncMtDloop* (AC027613.1) originates from the mitochondrial DNA (mtDNA), specifically located at chrM: 15,356–16,294 bp. (**B**) Examination of the sequence and evolutionary conservation of *lncMtDloop* across various species at the genomic locus of the *lncMtDloop* gene. (**C**) PhyloCSF analysis confirms the non-coding nature of *lncMtDloop*. The visualization employs a color scheme where the sense strand is depicted in green, indicating possible individual open reading frames. Conversely, the antisense strand is shown in red, also with potential open reading frames. The X-axis signifies the location along the RNA nucleotide sequence, while the Y-axis represents the PhyloCSF score. (**D**) Evaluation of coding potential scores for both coding genes, such as *Actb*, *Gapdh*, *Atp5b*, *Cox IV*, and non-coding genes like *Hotair*, *Xist*, and *lncMtDloop*. This assessment encompasses human, Macaca mulatta, and mouse species. (**E**) Utilization of a coding potential assessment tool to determine the coding probability of specific genes—*Actb*, *Gapdh*, *Atp5b*, *Cox IV* as well as non-coding genes *Hotair*, *Xis*t, and *lncMtDloop*—in human, *Macaca mulatta*, and mouse species. (**F**) Prediction of the secondary structures of *lncMtDloop* based on minimum free energy (MFE). The color scale bar, characterized by shades of red, indicates predictions with a high level of confidence. Source data are available online for this figure.

© The Author(s)

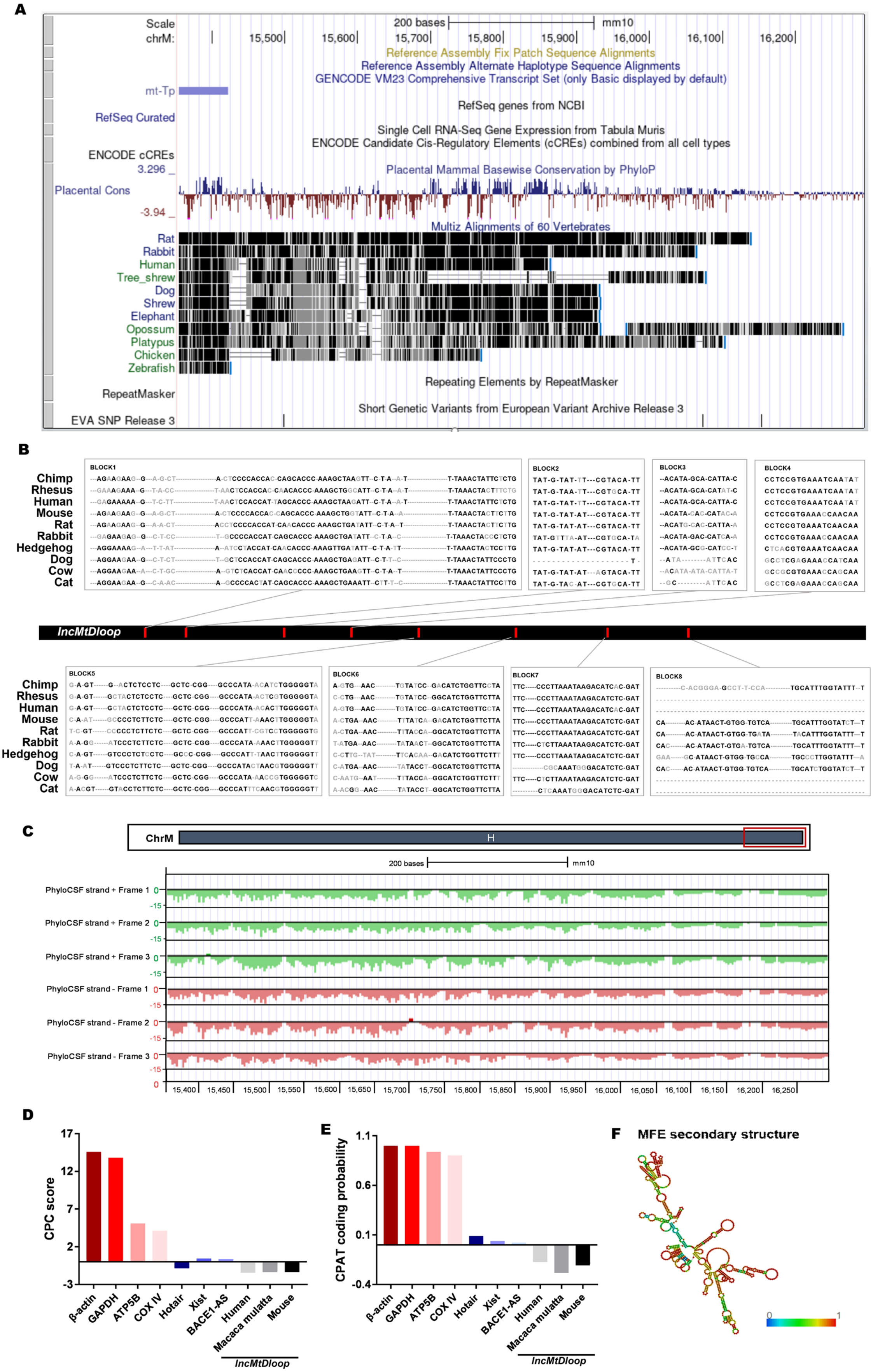
A
Scale
chrM:
200 bases
mm10
15,500
15,600
15,700
15,800
15,900
16,000
16,100
16,200
Reference Assembly Fix Patch Sequence Alignments
Reference Assembly Alternate Haplotype Sequence Alignments
GENCODE VM23 Comprehensive Transcript Set (only Basic displayed by default)
mt-Tp
RefSeq genes from NCBI
RefSeq Curated
Single Cell RNA-Seq Gene Expression from Tabula Muris
ENCODE Candidate Cis-Regulatory Elements (cCREs) combined from all cell types
ENCODE cCREs
3.296
Placental Mammal Basewise Conservation by PhyloP
Placental Cons
-3.94
Multiz Alignments of 60 Vertebrates
Rat
Rabbit
Human
Tree_shrew
Dog
Shrew
Elephant
Opossum
Platypus
Chicken
Zebrafish
Repeating Elements by RepeatMasker
RepeatMasker
Short Genetic Variants from European Variant Archive Release 3
EVA SNP Release 3
B
Chimp
Rhesus
Human
Mouse
Rat
Rabbit
Hedgehog
Dog
Cow
Cat
BLOCK1
BLOCK2
BLOCK3
BLOCK4
lncMtDloop
BLOCK5
BLOCK6
BLOCK7
BLOCK8
C
ChrM
H
200 bases
mm10
PhyloCSF strand + Frame 1
PhyloCSF strand + Frame 2
PhyloCSF strand + Frame 3
PhyloCSF strand - Frame 1
PhyloCSF strand - Frame 2
PhyloCSF strand - Frame 3
0
-15
15,400
15,450
15,500
15,550
15,600
15,650
15,700
15,750
15,800
15,850
15,900
15,950
16,000
16,050
16,100
16,150
16,200
16,250
D
CPC score
17
12
7
2
-3
β-actin
GAPDH
ATP5B
COX IV
Hotair
Xist
BACE1-AS
Human
Macaca mulatta
Mouse
lncMtDloop
E
CPAT coding probability
1.1
0.6
0.1
-0.4
β-actin
GAPDH
ATP5B
COX IV
Hotair
Xist
BACE1-AS
Human
Macaca mulatta
Mouse
lncMtDloop
F
MFE secondary structure
0
1

© The Author(s)

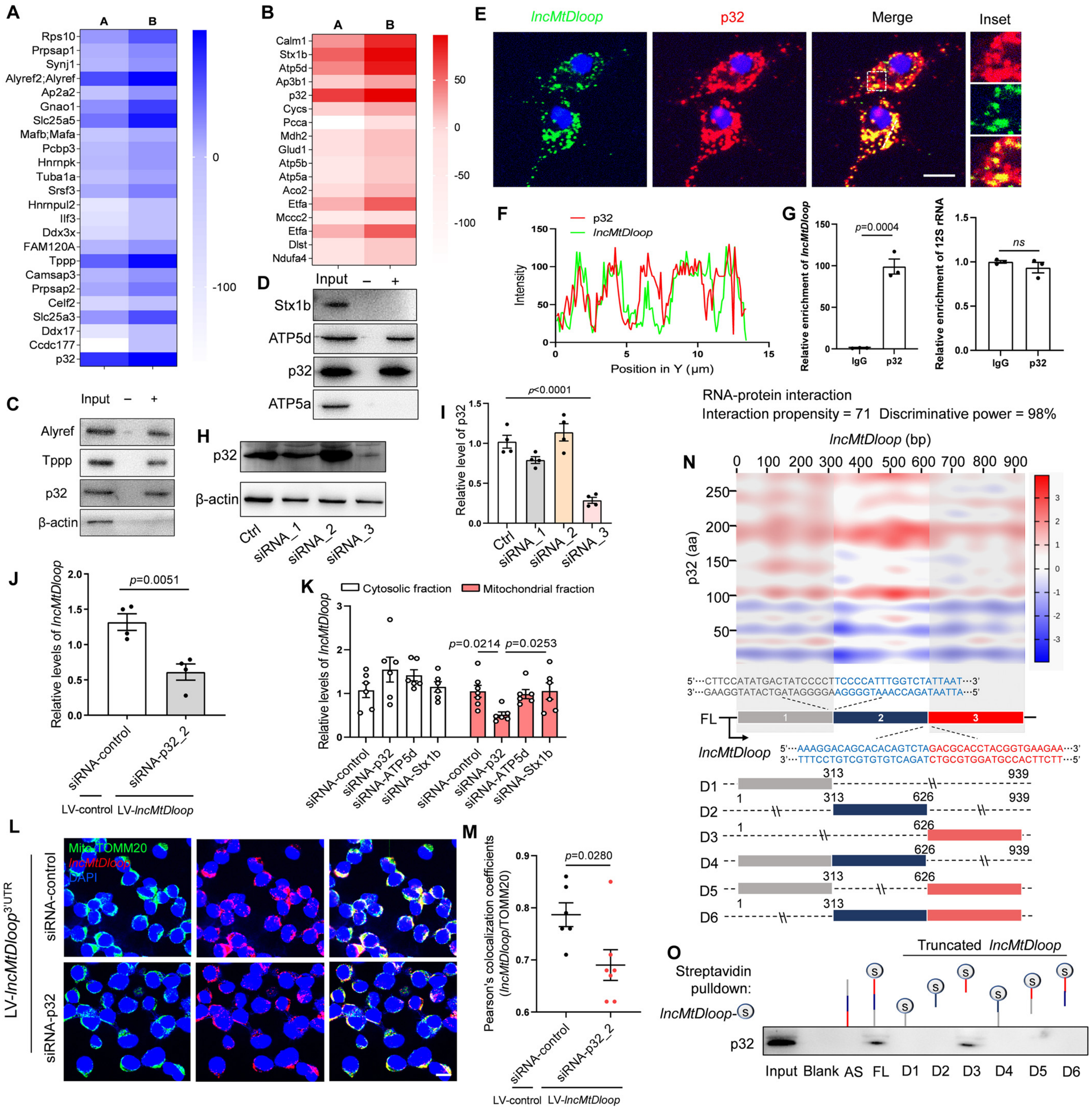
A
A B
Rps10
Prpsap1
Synj1
Alyref2;Alyref
Ap2a2
Gnao1
Slc25a5
Mafb;Mafa
Pcbp3
Hnrnpk
Tuba1a
Srsf3
Hnrnpul2
Ilf3
Ddx3x
FAM120A
Tppp
Camsap3
Prpsap2
Celf2
Slc25a3
Ddx17
Ccdc177
p32
0
-100
B
A B
Calm1
Stx1b
Atp5d
Ap3b1
p32
Cycs
Pcca
Mdh2
Glud1
Atp5b
Atp5a
Aco2
Etfa
Mccc2
Etfa
Dlst
Ndufa4
50
0
-50
-100
C
Input – +
Alyref
Tppp
p32
β-actin
D
Input – +
Stx1b
ATP5d
p32
ATP5a
E
lncMtDloop
p32
Merge
Inset
F
p32
lncMtDloop
Intensity
Position in Y (μm)
G
Relative enrichment of lncMtDloop
p=0.0004
IgG
p32
Relative enrichment of 12S rRNA
ns
H
p32
β-actin
Ctrl
siRNA_1
siRNA_2
siRNA_3
I
Relative level of p32
p<0.0001
Ctrl
siRNA_1
siRNA_2
siRNA_3
J
Relative levels of lncMtDloop
p=0.0051
siRNA-control
siRNA-p32_2
LV-control
LV-lncMtDloop
K
Cytosolic fraction
Mitochondrial fraction
Relative levels of lncMtDloop
p=0.0214
p=0.0253
siRNA-control
siRNA-p32
siRNA-ATP5d
siRNA-Stx1b
L
Mito-TOMM20
lncMtDloop
DAPI
LV-lncMtDloop3'UTR
siRNA-control
siRNA-p32
M
Pearson's colocalization coefficients (lncMtDloop/TOMM20)
p=0.0280
siRNA-control
siRNA-p32_2
LV-control
LV-lncMtDloop
N
RNA-protein interaction
Interaction propensity = 71 Discriminative power = 98%
lncMtDloop (bp)
p32 (aa)
5'···CTTCCATATGACTATCCCCTTCCCCATTTGGTCTATTAAT···3'
3'···GAAGGTATACTGATAGGGGAAGGGGTAAACCAGATAATTA···5'
FL
lncMtDloop
5'···AAAGGACAGCACACAGTCTAGACGCACCTACGGTGAAGAA···3'
3'···TTTCCTGTCGTGTGTCAGATCTGCGTGGATGCCACTTCTT···5'
D1
D2
D3
D4
D5
D6
O
Streptavidin pulldown:
lncMtDloop-S
Truncated lncMtDloop
p32
Input Blank AS FL D1 D2 D3 D4 D5 D6

 © The Author(s)

Figure EV2. Contribution of p32 to *lncMtDloop* localization within mitochondria, related to Fig. 2.

(**A**) Predicted interaction propensity of *lncMtDloop* with binding partners in cytosolic preparations based on catRAPID, corresponding to mass spectrum results. The left column displays the interaction propensity scores of proteins, while the right column represents their discriminative power in a centesimal system. (**B**) Predicted interaction propensity of *lncMtDloop* with binding partners in mitochondrial preparations based on catRAPID, corresponding to mass spectrum results. The left column displays the interaction propensity scores of proteins, while the right column represents their discriminative power in a centesimal system. (**C**, **D**) RNA pull-down assays utilizing biotinylated *lncMtDloop* and antisense-*lncMtDloop* in cytosolic preparations (**C**) and mitochondrial (**D**), followed by western blot analysis of Alyref, Tppp, Stx1b, ATP5d, and p32. "+" represents *lncMtDloop* probe, and "−" represents anti-*lncMtDloop* control. (**E**) Representative images displaying the co-localization of *lncMtDloop* (green) and p32 (red) using RNAscope ISH and immunostaining in neurons. Scale bars, 10 μm. (**F**) Quantification of fluorescence signal intensities for *lncMtDloop* and p32 along the white dotted line in panel (**E**), measured using Image J. (**G**) RT-qPCR analysis for detectable *lncMtDloop* from RIP analysis of p32 antibody. 12 s RNA was used as control. Results were normalized to the control IgG RIP group. Bars represent mean ± SEM, with $n = 3$ independent experiments per group, unpaired *t*-test. (**H**, **I**) Knockdown of p32 in N2a cells using three siRNAs. The western blot analysis of p32 was performed. Bars represent mean ± SEM, $n = 3$ repetitions per group, unpaired *t*-test. (**J**) RT-qPCR analysis demonstrating the effect of p32 knockdown on *lncMtDloop* distribution in mitochondrial fractions. Bars represent mean ± SEM, with $n = 4$ repetitions per group, unpaired *t*-test. (**K**) RT-qPCR analysis revealing the impact of p32, ATP5d, and Stx1b knockdown on *lncMtDloop* distribution in mitochondrial fractions. N2a cells were infected with LV-*lncMtDloop* followed by siRNA-p32 transfection. Bars represent mean ± SEM, with $n = 6$ repetitions per group, one-way ANOVA with Dunnett's multiple comparisons. (**L**) Representative images showing the co-localization of *lncMtDloop* (red) and ATP5a (green) in N2a cells. LV-*lncMtDloop*-infected N2a cells were transfected with siRNA against p32. Scale bars, 10 μm. (**M**) Quantification of co-localization as observed in panel K). Bars represent mean ± SEM, with $n = 6$ regions of interest (ROI) per group, unpaired *t*-test. (**N**) Heatmap illustrating the interaction propensity of *lncMtDloop* binding to p32, as predicted by catRAPID. The X-axis indicates RNA nucleotide sequence location, while the Y-axis indicates protein residue location. Red shades indicate predictions with high interaction propensity. Bottom: Schematic map depicting the construction of six truncated *lncMtDloop* mutants to identify its protein-binding motifs. (**O**) In vitro transcription of the indicated six *lncMtDloop* truncates, followed by RNA pull-down assays using streptavidin-tagged RNA. Source data are available online for this figure.

© The Author(s)

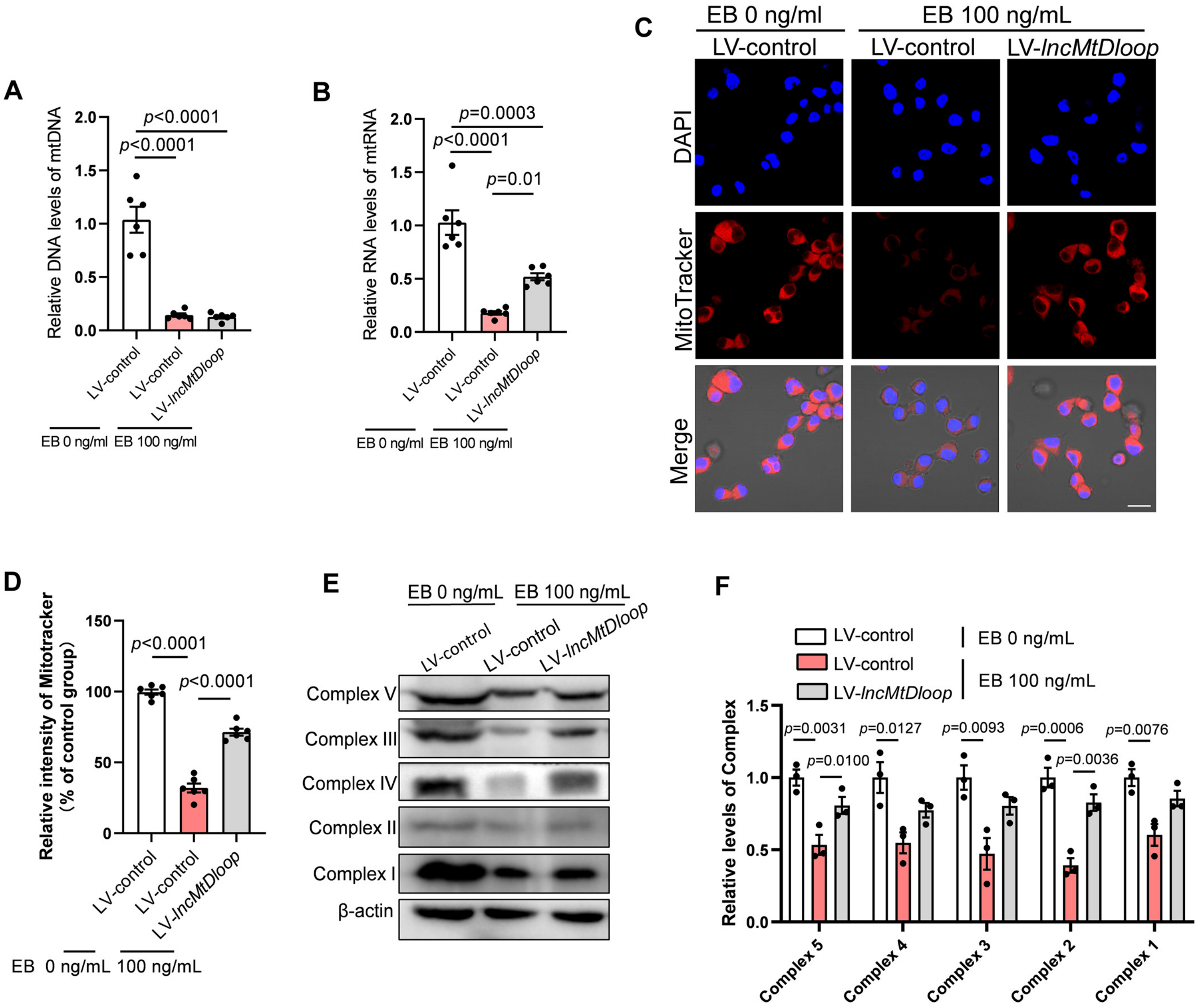

Figure EV3. *lncMtDloop* promotes mtDNA transcription in mtDNA depletion assay, related to Fig. 3.

(**A**) Determination of mtDNA copy number by qPCR. Error bars denote mean ± SEM, with $n = 6$ repetitions per group, one-way ANOVA followed by Dunnett's multiple comparisons test. (**B**) Determination of mtRNA copy number by RT-qPCR. Error bars denote mean ± SEM, with $n = 6$ repetitions per group, one-way ANOVA followed by Dunnett's multiple comparisons test. (**C**) Representative images depicting the MitoTrack signals in the N2a cell. Scale bars, 20 μm. (**D**) Quantification of fluorescence signal intensities for mitochondria. Error bars denote mean ± SEM, with $n = 6$ repetitions per group, one-way ANOVA followed by Dunnett's multiple comparisons test. (**E**) Western blot analysis depicting the levels of OXPHOS subunit proteins upon treatment with LV-*lncMtDloop* in primary cultured neurons w/o EB treatment. (**F**) Relative intensities of signals for OXPHOS subunit proteins. Error bars denote mean ± SEM, with $n = 3$ repetitions per group, one-way ANOVA followed by Dunnett's multiple comparisons test. Source data are available online for this figure.

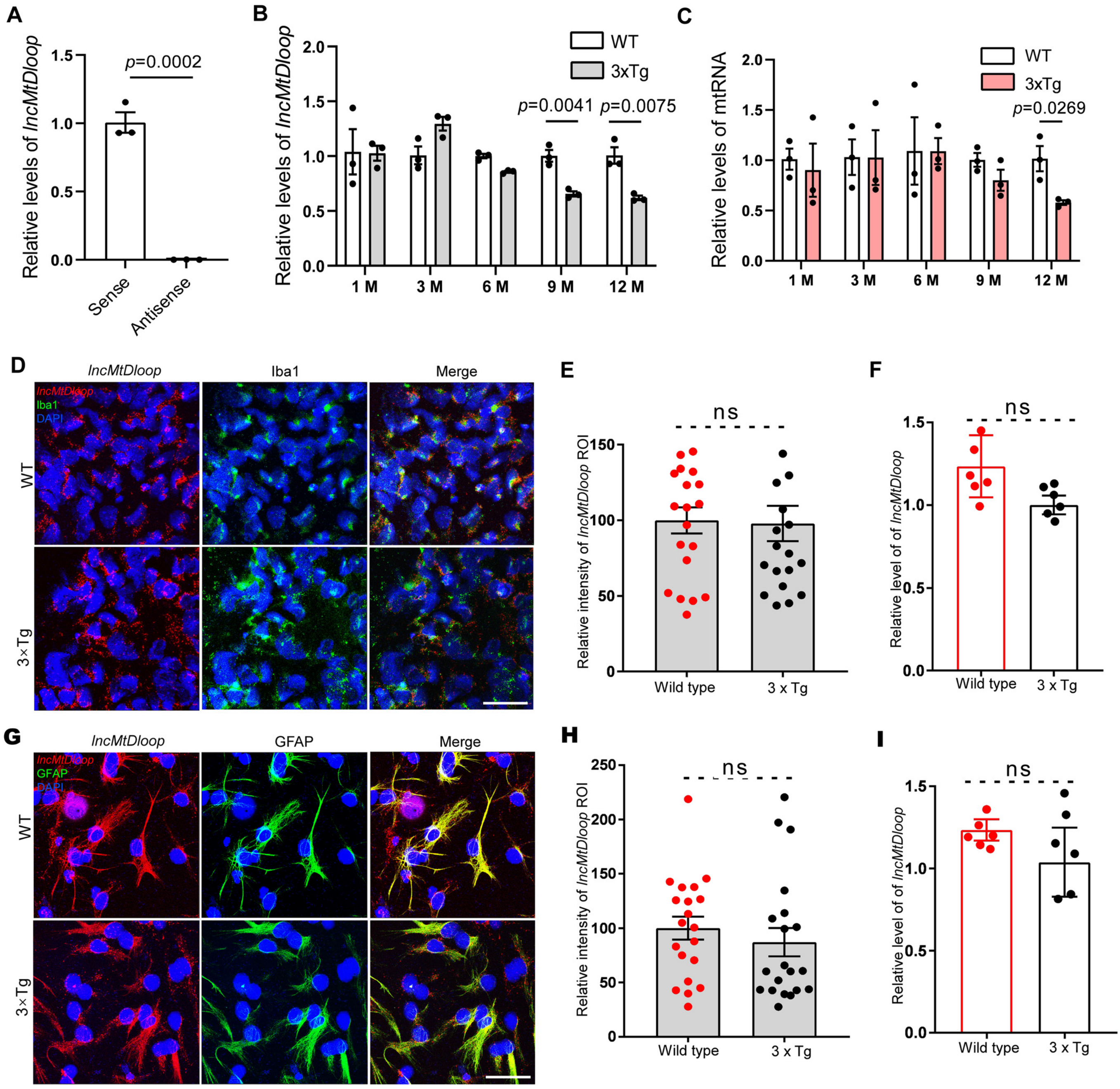

Figure EV4. Decreased expression of *lncMtDloop* differentially occurs in distinct neural cell types of AD, related to Fig. 4.

(**A**) Determination of *lncMtDloop* levels by RT-qPCR. Antisense was used as control. Error bars denote mean ± SEM, with *n* = 3 repetitions per group, unpaired *t*-test. (**B**) Determination of *lncMtDloop* levels by RT-qPCR. Error bars denote mean ± SEM, with *n* = 3 mice per group, unpaired *t*-test. (**C**) Determination of mtRNA copy number by RT-qPCR. Error bars denote mean ± SEM, with *n* = 3 mice per group, unpaired *t*-test. (**D**) Representative images of RNAscope ISH and immunostaining showing few changes in levels of *lncMtDloop* (red) expression in 3xTg hippocampal microglia (Iba1, green) cultures at DIV14. Scale bars, 50 µm. (**E**) Relative intensities of *lncMtDloop* fluorescent signals are illustrated in (**D**). Bars indicate mean ± SEM, *n* (cells) = 17–19 per group. By unpaired *t*-test. (**F**) RT-qPCR analysis of *lncMtDloop* expression in wild type and 3xTg hippocampal microglia cultures at DIV14. Bars = mean ± SEM, *n* = 6 repetitions per group, unpaired *t*-test. (**G**) Representative images of RNAscope ISH and immunostaining showing few changes in levels of *lncMtDloop* (red) expression in 3xTg hippocampal astrocyte (GFAP, green) cultures at DIV14. Scale bars, 50 µm. (**H**) Relative intensities of *lncMtDloop* fluorescent signals are illustrated in (**G**). Bars indicate mean ± SEM, *n* (cells) = 20 per group. By unpaired *t*-test. (**I**) RT-qPCR analysis of *lncMtDloop* expression in wild type and 3xTg hippocampal astrocyte cultures at DIV14. Bars = mean ± SEM, *n* = 6 repetitions per group, unpaired *t*-test.

© The Author(s)

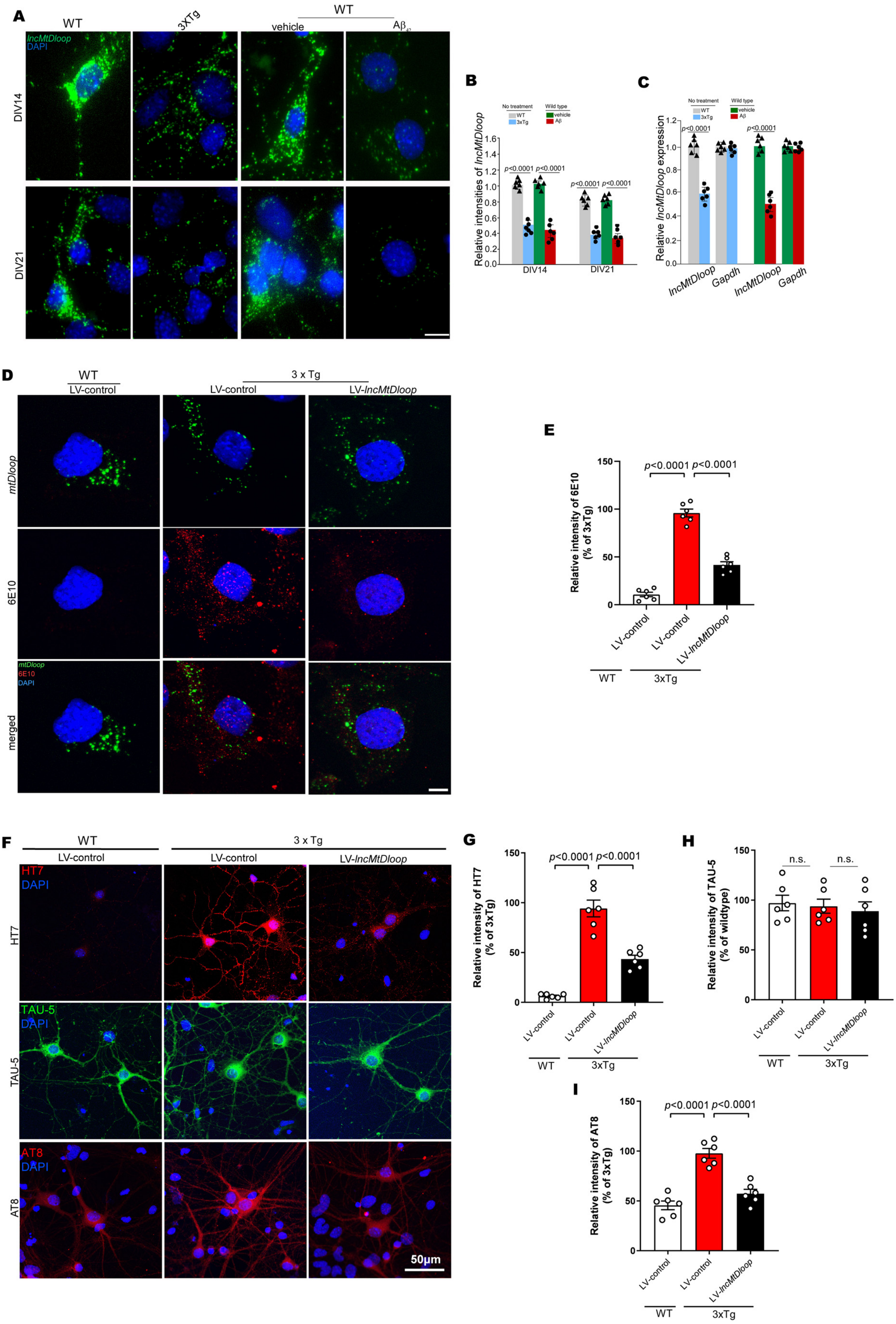
A
WT
3XTg
WT
vehicle
Aβ
lncMtDloop
DAPI
DIV14
DIV21
B
No treatment
WT
3xTg
Wild type
vehicle
Aβ
Relative intensities of lncMtDloop
p<0.0001
DIV14
DIV21
C
Relative lncMtDloop expression
lncMtDloop
Gapdh
D
WT
LV-control
3 x Tg
LV-control
LV-lncMtDloop
mtDloop
6E10
merged
E
Relative intensity of 6E10 (% of 3xTg)
p<0.0001
LV-control
LV-lncMtDloop
WT
3xTg
F
HT7
TAU-5
AT8
DAPI
50μm
G
Relative intensity of HT7 (% of 3xTg)
H
Relative intensity of TAU-5 (% of wildtype)
n.s.
I
Relative intensity of AT8 (% of 3xTg)

Figure EV5. *LncMtDloop* expression is mutually related to AD pathology, related to Fig. 5.

(**A**) Representative image of RNAscope ISH showing *lncMtDloop* expression. Wild-type (WT) and 3 × Tg primary hippocampal neurons at DIV14 and DIV21 were performed RNAscope ISH with mouse-specific probe sets against *lncMtDloop* with the treatment of $A\beta_{42}$. Scale bars, 10 µm. (**B**) Quantification of *lncMtDloop* fluorescent intensity illustrated in (**A**). Bars indicate mean ± SEM, $n = 6$ images per group, by unpaired *t*-test. (**C**) RT-qPCR analysis of *lncMtDloop* expression in hippocampal neurons of wild type and 3 × Tg at DIV14 with $A\beta_{42}$ treatment. Bars indicate mean ± SEM, $n = 6$ repetitions per group, by unpaired *t*-test. (**D**) Representative images of RNAscope ISH and immunostaining showing *lncMtDloop* expression (green) and Aβ production (red). Scale bars, 5 µm. WT and 3xTg primary hippocampal neurons were infected with LV-*lncMtDloop* or LV-control at DIV5, cells were prepared for tests at DIV14. (**E**) Quantification of fluorescence intensities of 6E10 signals illustrated in (**D**). Bars indicate mean ± SEM, $n = 6$ images per group, by one-way ANOVA with Dunnett's multiple comparisons test. (**F**) Representative IF images showing HT7, TAU-5, and AT8 in wild type and 3xTg primary hippocampal neurons. Wild type and 3xTg primary hippocampal neurons were infected with LV-*lncMtDloop* at DIV5, cells were collected and prepared for tests at DIV14. (**G–I**) Relative intensities of HT7, TAU-5, and AT8 fluorescent signals illustrated in (**F**). Bars indicate mean ± SEM, $n = 6$ images per group, one-way ANOVA with Dunnett's multiple comparisons test. Source data are available online for this figure.

© The Author(s)
